# Supplementary material for: Characterization of a novel OX40 ligand and CD40 ligand-expressing oncolytic adenovirus used in the PeptiCRAd cancer vaccine platform
Source: Mol Ther Oncolytics. 2021 Feb 10;20:459–69. doi: 10.1016/j.omto.2021.02.006 (PMC7917457; doi:10.1016/j.omto.2021.02.006)
Supplement: Document S1. Figures S1–S5 [file mmc1.pdf]

OMTO, Volume *20*

Supplemental information

Characterization of a novel OX40 ligand and CD40 ligand-expressing oncolytic adenovirus used in the PeptiCRAd cancer vaccine platform

Erkko Ylösmäki, Leena Ylösmäki, Manlio Fusciello, Beatriz Martins, Petra Ahokas, Hanne Cojoc, Arttu Uoti, Sara Feola, Anna Kreutzman, Tuuli Ranki, Julia Karbach, Tapani Viitala, Petri Priha, Elke Jäger, Sari Pesonen, and Vincenzo Cerullo

Supplemental Information

Fig. S1. Individual tumour growth curves of oncolytic potency of VALO-D102 presented in Figure 3 panel F.

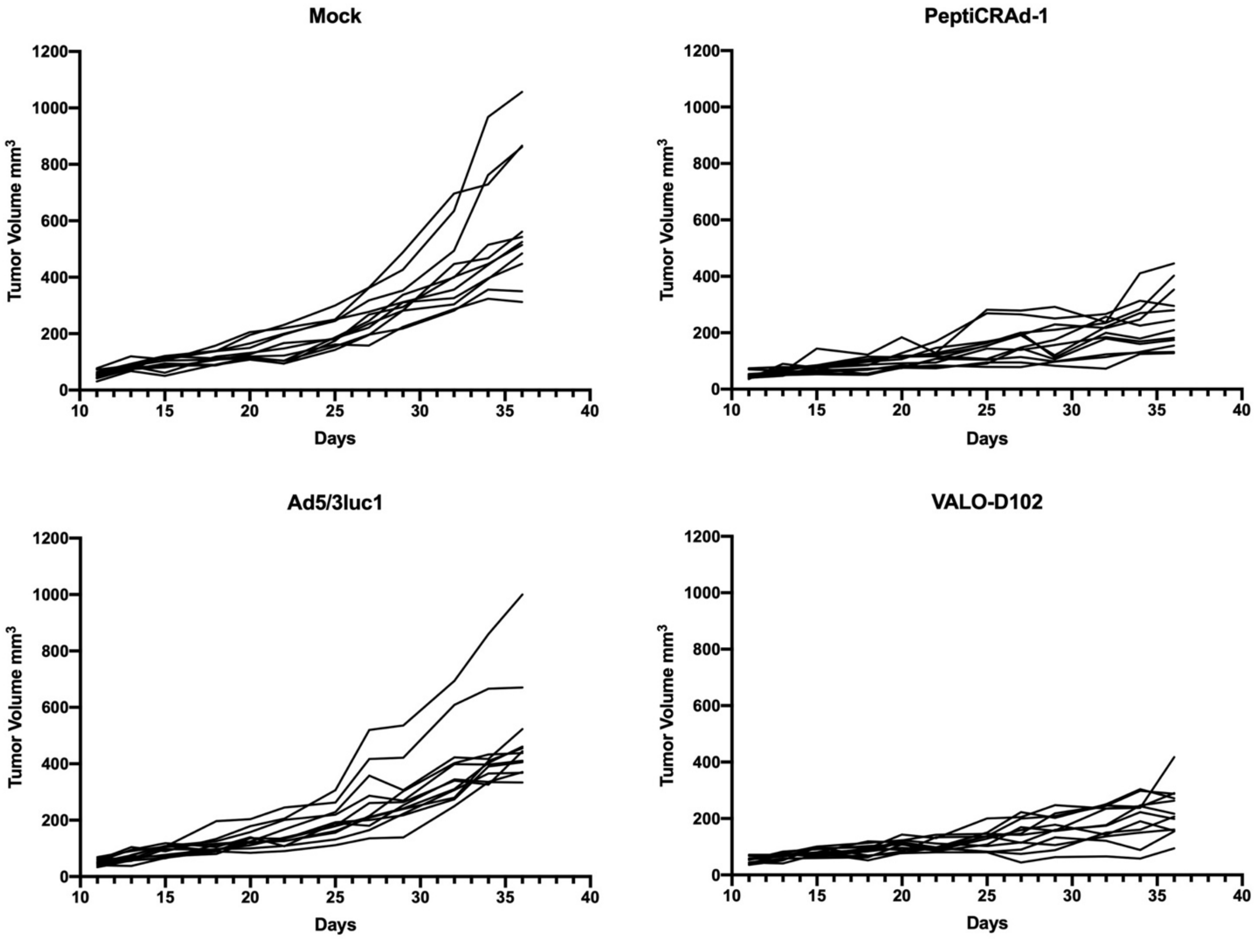

Fig. S2. Individual tumour growth curves for the experiment presented in Figure 4.

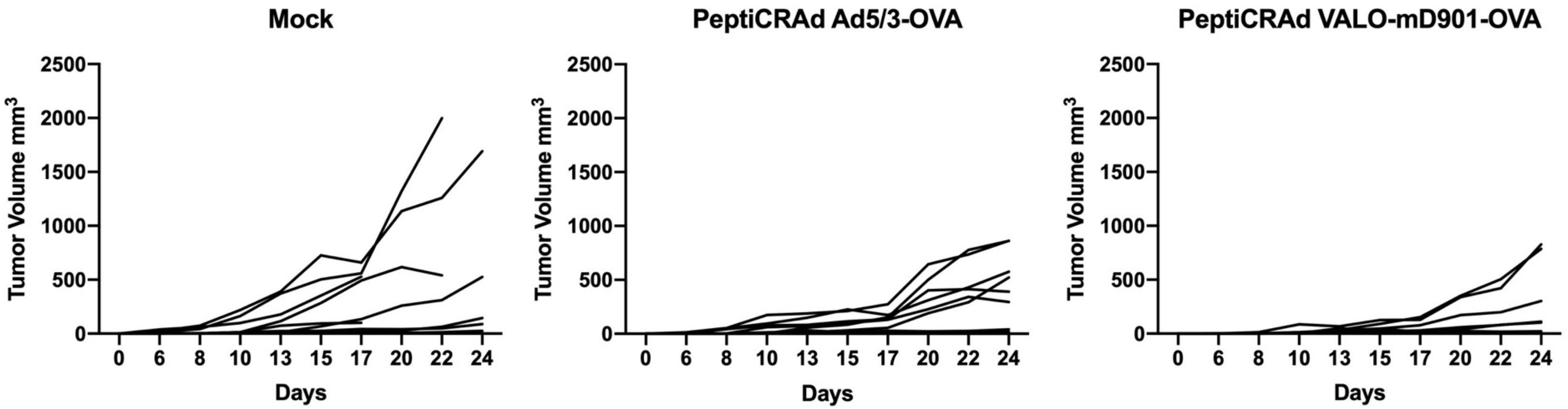

Fig. S3. Individual tumour growth curves for the experiment presented in Figure 5.

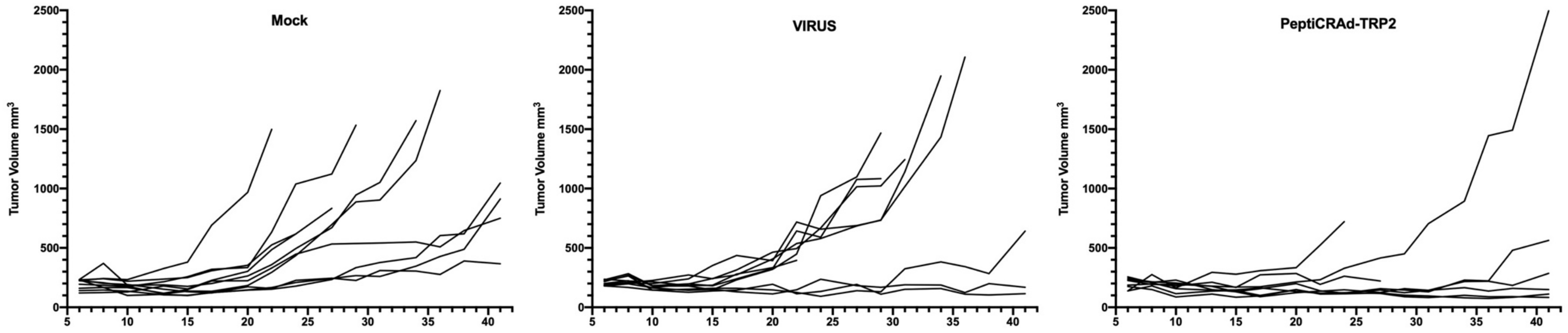

Fig. S4. Individual tumour growth curves for the experiment presented in Figure 6.

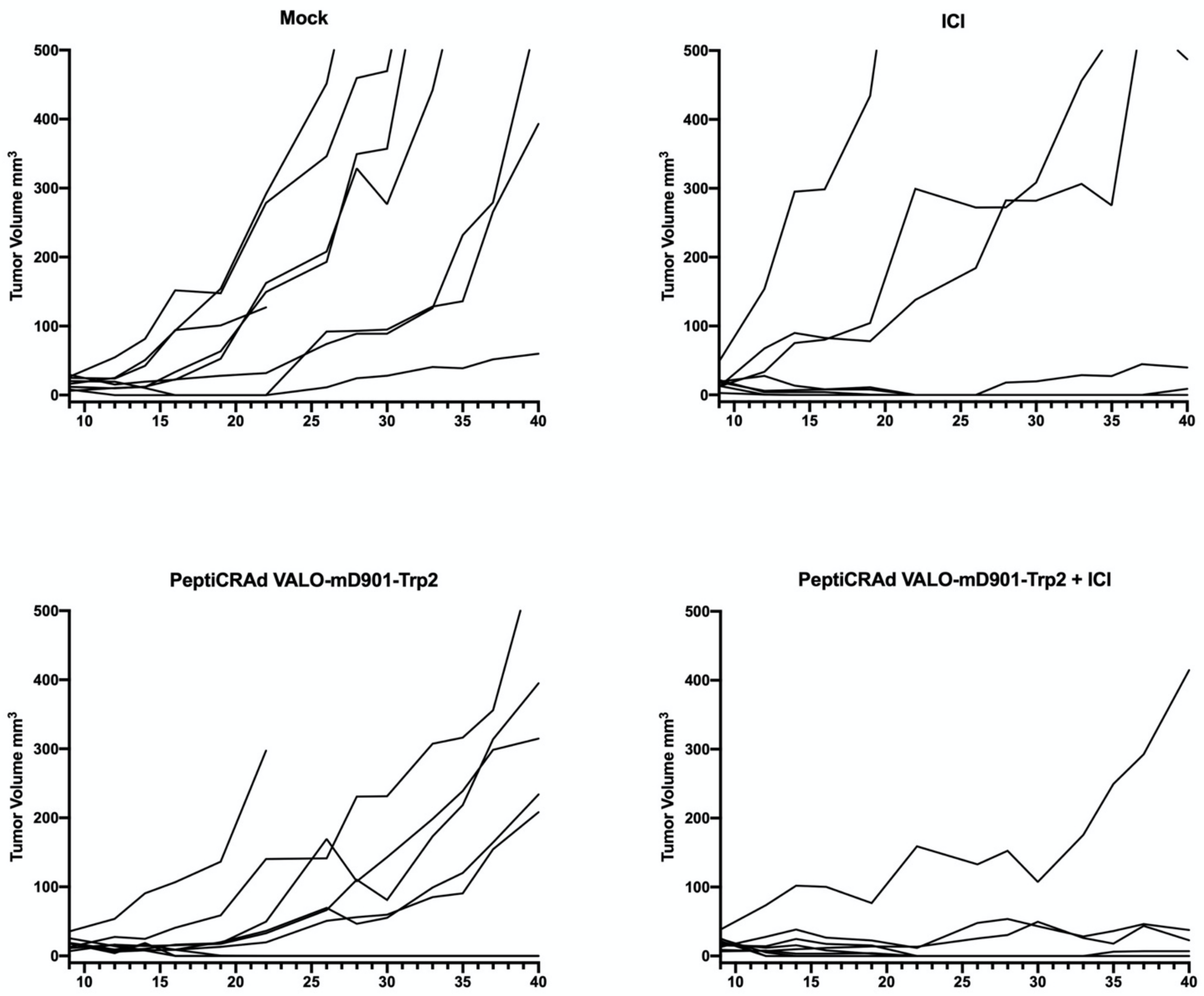

Fig. S5. Alignment of sequence analysis from directly after plaque purification and after seven rounds of amplification.
