# Supplementary material for: Characterization of a novel OX40 ligand and CD40 ligand-expressing oncolytic adenovirus used in the PeptiCRAd cancer vaccine platform
Source: Mol Ther Oncolytics. 2021 Feb 10;20:459–69. doi: 10.1016/j.omto.2021.02.006 (PMC7917457; doi:10.1016/j.omto.2021.02.006)
Supplement: Data S1. Aligned using an external algorithm [file mmc2.pdf]

# Aligned using an external algorithm

**Consensus**

1. First analysis after plaque purification
2. Analysis after seven cycles of amplification

```
1    1    CATCATCAATAATATACCTTATTTTGGATTGAAGCCAATATGATAATGAGGGGGTGGAGT  60
          ||||||||||||||||||||||||||||||||||||||||||||||||||||||||||||
2    1    CATCATCAATAATATACCTTATTTTGGATTGAAGCCAATATGATAATGAGGGGGTGGAGT  60

1    61   TTGTGACGTGGCGCGGGGCGTGGGAACGGGGCGGGTGACGTAGTAGTGTGGCGGAAGTGT  120
          ||||||||||||||||||||||||||||||||||||||||||||||||||||||||||||
2    61   TTGTGACGTGGCGCGGGGCGTGGGAACGGGGCGGGTGACGTAGTAGTGTGGCGGAAGTGT  120

1    121  GATGTTGCAAGTGTGGCGGAACACATGTAAGCGACGGATGTGGCAAAAGTGACGTTTTTG  180
          ||||||||||||||||||||||||||||||||||||||||||||||||||||||||||||
2    121  GATGTTGCAAGTGTGGCGGAACACATGTAAGCGACGGATGTGGCAAAAGTGACGTTTTTG  180

1    181  GTGTGCGCCGGTGTACACAGGAAGTGACAATTTTCGCGCGGTTTTAGGCGGATGTTGTAG  240
          ||||||||||||||||||||||||||||||||||||||||||||||||||||||||||||
2    181  GTGTGCGCCGGTGTACACAGGAAGTGACAATTTTCGCGCGGTTTTAGGCGGATGTTGTAG  240

1    241  TAAATTTGGGCGTAACCGAGTAAGATTTGGCCATTTTCGCGGGAAAACTGAATAAGAGGA  300
          ||||||||||||||||||||||||||||||||||||||||||||||||||||||||||||
2    241  TAAATTTGGGCGTAACCGAGTAAGATTTGGCCATTTTCGCGGGAAAACTGAATAAGAGGA  300

1    301  AGTGAAATCTGAATAATTTTGTGTTACTCATAGCGCGTAATATTTGTCTAGGGCCGCGGG  360
          ||||||||||||||||||||||||||||||||||||||||||||||||||||||||||||
2    301  AGTGAAATCTGAATAATTTTGTGTTACTCATAGCGCGTAATATTTGTCTAGGGCCGCGGG  360

1    361  GACTTTGACCGTTTACGTGGAGACTCGCCCAGGTGTTTTTCTCAGGTGTTTTCCGCGTTC  420
          ||||||||||||||||||||||||||||||||||||||||||||||||||||||||||||
2    361  GACTTTGACCGTTTACGTGGAGACTCGCCCAGGTGTTTTTCTCAGGTGTTTTCCGCGTTC  420

1    421  CGGGTCAAAGTTGGCGTTTTATTATTATAGTCAGCTGACGTGTAGTGTATTTATACCCGG  480
          ||||||||||||||||||||||||||||||||||||||||||||||||||||||||||||
2    421  CGGGTCAAAGTTGGCGTTTTATTATTATAGTCAGCTGACGTGTAGTGTATTTATACCCGG  480

1    481  TGAGTTCCTCAAGAGGCCACTCTTGAGTGCCAGCGAGTAGAGTTTTCTCCTCCGAGCCGC  540
          ||||||||||||||||||||||||||||||||||||||||||||||||||||||||||||
2    481  TGAGTTCCTCAAGAGGCCACTCTTGAGTGCCAGCGAGTAGAGTTTTCTCCTCCGAGCCGC  540
```

```
1   541   TCCGACACCGGGACTGAAAATGAGACATATTATCTGCCACGGAGGTGTTATTACCGAAGA  600
          ||||||||||||||||||||||||||||||||||||||||||||||||||||||||||||
2   541   TCCGACACCGGGACTGAAAATGAGACATATTATCTGCCACGGAGGTGTTATTACCGAAGA  600

1   601   AATGGCCGCCAGTCTTTTGGACCAGCTGATCGAAGAGGTACTGGCTGATAATCTTCCACC  660
          ||||||||||||||||||||||||||||||||||||||||||||||||||||||||||||
2   601   AATGGCCGCCAGTCTTTTGGACCAGCTGATCGAAGAGGTACTGGCTGATAATCTTCCACC  660

1   661   TCCTAGCCATTTTGAACCACCTACCCTTCACGAACTGTATGATTTAGACGTGACGGCCCC  720
          ||||||||||||||||||||||||||||||||||||||||||||||||||||||||||||
2   661   TCCTAGCCATTTTGAACCACCTACCCTTCACGAACTGTATGATTTAGACGTGACGGCCCC  720

1   721   CGAAGATCCCAACGAGGAGGCGGTTTCGCAGATTTTTCCCGACTCTGTAATGTTGGCGGT  780
          ||||||||||||||||||||||||||||||||||||||||||||||||||||||||||||
2   721   CGAAGATCCCAACGAGGAGGCGGTTTCGCAGATTTTTCCCGACTCTGTAATGTTGGCGGT  780

1   781   GCAGGAAGGGATTGACTTACTCACTTTTCCGCCGGCGCCCGGTTCTCCGGAGCCGCCTCA  840
          ||||||||||||||||||||||||||||||||||||||||||||||||||||||||||||
2   781   GCAGGAAGGGATTGACTTACTCACTTTTCCGCCGGCGCCCGGTTCTCCGGAGCCGCCTCA  840

1   841   CCTTTCCCGGCAGCCCGAGCAGCCGGAGCAGAGAGCCTTGGGTCCGGTTTCTATGCCAAA  900
          ||||||||||||||||||||||||||||||||||||||||||||||||||||||||||||
2   841   CCTTTCCCGGCAGCCCGAGCAGCCGGAGCAGAGAGCCTTGGGTCCGGTTTCTATGCCAAA  900

1   901   CCTTGTACCGGAGGTGATCGATCCACCCAGTGACGACGAGGATGAAGAGGGTGAGGAGTT  960
          ||||||||||||||||||||||||||||||||||||||||||||||||||||||||||||
2   901   CCTTGTACCGGAGGTGATCGATCCACCCAGTGACGACGAGGATGAAGAGGGTGAGGAGTT  960

1   961   TGTGTTAGATTATGTGGAGCACCCCGGGCACGGTTGCAGGTCTTGTCATTATCACCGGAG  1020
          ||||||||||||||||||||||||||||||||||||||||||||||||||||||||||||
2   961   TGTGTTAGATTATGTGGAGCACCCCGGGCACGGTTGCAGGTCTTGTCATTATCACCGGAG  1020

1   1021  GAATACGGGGGACCCAGATATTATGTGTTCGCTTTGCTATATGAGGACCTGTGGCATGTT  1080
          ||||||||||||||||||||||||||||||||||||||||||||||||||||||||||||
2   1021  GAATACGGGGGACCCAGATATTATGTGTTCGCTTTGCTATATGAGGACCTGTGGCATGTT  1080

1   1081  TGTCTACAGTAAGTGAAAATTATGGGCAGTGGGTGATAGAGTGGTGGGTTTGGTGTGGTA  1140
          ||||||||||||||||||||||||||||||||||||||||||||||||||||||||||||
2   1081  TGTCTACAGTAAGTGAAAATTATGGGCAGTGGGTGATAGAGTGGTGGGTTTGGTGTGGTA  1140
```

```
1    1141  ATTTTTTTTTTAATTTTTACAGTTTTGTGGTTTAAAGAATTTTGTATTGTGATTTTTTTA  1200
           ||||||||||||||||||||||||||||||||||||||||||||||||||||||||||||
2    1141  ATTTTTTTTTTAATTTTTACAGTTTTGTGGTTTAAAGAATTTTGTATTGTGATTTTTTTA  1200

1    1201  AAAGGTCCTGTGTCTGAACCTGAGCCTGAGCCCGAGCCAGAACCGGAGCCTGCAAGACCT  1260
           ||||||||||||||||||||||||||||||||||||||||||||||||||||||||||||
2    1201  AAAGGTCCTGTGTCTGAACCTGAGCCTGAGCCCGAGCCAGAACCGGAGCCTGCAAGACCT  1260

1    1261  ACCCGCCGTCCTAAAATGGCGCCTGCTATCCTGAGACGCCCGACATCACCTGTGTCTAGA  1320
           ||||||||||||||||||||||||||||||||||||||||||||||||||||||||||||
2    1261  ACCCGCCGTCCTAAAATGGCGCCTGCTATCCTGAGACGCCCGACATCACCTGTGTCTAGA  1320

1    1321  GAATGCAATAGTAGTACGGATAGCTGTGACTCCGGTCCTTCTAACACACCTCCTGAGATA  1380
           ||||||||||||||||||||||||||||||||||||||||||||||||||||||||||||
2    1321  GAATGCAATAGTAGTACGGATAGCTGTGACTCCGGTCCTTCTAACACACCTCCTGAGATA  1380

1    1381  CACCCGGTGGTCCCGCTGTGCCCCATTAAACCAGTTGCCGTGAGAGTTGGTGGGCGTCGC  1440
           ||||||||||||||||||||||||||||||||||||||||||||||||||||||||||||
2    1381  CACCCGGTGGTCCCGCTGTGCCCCATTAAACCAGTTGCCGTGAGAGTTGGTGGGCGTCGC  1440

1    1441  CAGGCTGTGGAATGTATCGAGGACTTGCTTAACGAGCCTGGGCAACCTTTGGACTTGAGC  1500
           ||||||||||||||||||||||||||||||||||||||||||||||||||||||||||||
2    1441  CAGGCTGTGGAATGTATCGAGGACTTGCTTAACGAGCCTGGGCAACCTTTGGACTTGAGC  1500

1    1501  TGTAAACGCCCCAGGCCATAAGGTGTAAACCTGTGATTGCGTGTGTGGTTAACGCCTTTG  1560
           ||||||||||||||||||||||||||||||||||||||||||||||||||||||||||||
2    1501  TGTAAACGCCCCAGGCCATAAGGTGTAAACCTGTGATTGCGTGTGTGGTTAACGCCTTTG  1560

1    1561  TTTGCTGAATGAGTTGATGTAAGTTTAATAAAGGGTGAGATAATGTTTAACTTGCATGGC  1620
           ||||||||||||||||||||||||||||||||||||||||||||||||||||||||||||
2    1561  TTTGCTGAATGAGTTGATGTAAGTTTAATAAAGGGTGAGATAATGTTTAACTTGCATGGC  1620

1    1621  GTGTTAAATGGGGCGGGGCTTAAAGGGTATATAATGCGCCGTGGGCTAATCTTGGTTACA  1680
           ||||||||||||||||||||||||||||||||||||||||||||||||||||||||||||
2    1621  GTGTTAAATGGGGCGGGGCTTAAAGGGTATATAATGCGCCGTGGGCTAATCTTGGTTACA  1680

1    1681  TCTGACCTCATGGAGGCTTGGGAGTGTTTGGAAGATTTTTCTGCTGTGCGTAACTTGCTG  1740
           ||||||||||||||||||||||||||||||||||||||||||||||||||||||||||||
2    1681  TCTGACCTCATGGAGGCTTGGGAGTGTTTGGAAGATTTTTCTGCTGTGCGTAACTTGCTG  1740
```

```
1   1741  GAACAGAGCTCTAACAGTACCTCTTGGTTTTGGAGGTTTCTGTGGGGCTCATCCCAGGCA  1800
          ||||||||||||||||||||||||||||||||||||||||||||||||||||||||||||
2   1741  GAACAGAGCTCTAACAGTACCTCTTGGTTTTGGAGGTTTCTGTGGGGCTCATCCCAGGCA  1800

1   1801  AAGTTAGTCTGCAGAATTAAGGAGGATTACAAGTGGGAATTTGAAGAGCTTTTGAAATCC  1860
          ||||||||||||||||||||||||||||||||||||||||||||||||||||||||||||
2   1801  AAGTTAGTCTGCAGAATTAAGGAGGATTACAAGTGGGAATTTGAAGAGCTTTTGAAATCC  1860

1   1861  TGTGGTGAGCTGTTTGATTCTTTGAATCTGGGTCACCAGGCGCTTTTCCAAGAGAAGGTC  1920
          ||||||||||||||||||||||||||||||||||||||||||||||||||||||||||||
2   1861  TGTGGTGAGCTGTTTGATTCTTTGAATCTGGGTCACCAGGCGCTTTTCCAAGAGAAGGTC  1920

1   1921  ATCAAGACTTTGGATTTTTCCACACCGGGGCGCGCTGCGGCTGCTGTTGCTTTTTTGAGT  1980
          ||||||||||||||||||||||||||||||||||||||||||||||||||||||||||||
2   1921  ATCAAGACTTTGGATTTTTCCACACCGGGGCGCGCTGCGGCTGCTGTTGCTTTTTTGAGT  1980

1   1981  TTTATAAAGGATAAATGGAGCGAAGAAACCCATCTGAGCGGGGGGTACCTGCTGGATTTT  2040
          ||||||||||||||||||||||||||||||||||||||||||||||||||||||||||||
2   1981  TTTATAAAGGATAAATGGAGCGAAGAAACCCATCTGAGCGGGGGGTACCTGCTGGATTTT  2040

1   2041  CTGGCCATGCATCTGTGGAGAGCGGTTGTGAGACACAAGAATCGCCTGCTACTGTTGTCT  2100
          ||||||||||||||||||||||||||||||||||||||||||||||||||||||||||||
2   2041  CTGGCCATGCATCTGTGGAGAGCGGTTGTGAGACACAAGAATCGCCTGCTACTGTTGTCT  2100

1   2101  TCCGTCCGCCCGGCGATAATACCGACGGAGGAGCAGCAGCAGCAGCAGGAGGAAGCCAGG  2160
          ||||||||||||||||||||||||||||||||||||||||||||||||||||||||||||
2   2101  TCCGTCCGCCCGGCGATAATACCGACGGAGGAGCAGCAGCAGCAGCAGGAGGAAGCCAGG  2160

1   2161  CGGCGGCGGCAGGAGCAGAGCCCATGGAACCCGAGAGCCGGCCTGGACCCTCGGGAATGA  2220
          ||||||||||||||||||||||||||||||||||||||||||||||||||||||||||||
2   2161  CGGCGGCGGCAGGAGCAGAGCCCATGGAACCCGAGAGCCGGCCTGGACCCTCGGGAATGA  2220

1   2221  ATGTTGTACAGGTGGCTGAACTGTATCCAGAACTGAGACGCATTTTGACAATTACAGAGG  2280
          ||||||||||||||||||||||||||||||||||||||||||||||||||||||||||||
2   2221  ATGTTGTACAGGTGGCTGAACTGTATCCAGAACTGAGACGCATTTTGACAATTACAGAGG  2280

1   2281  ATGGGCAGGGGCTAAAGGGGGTAAAGAGGGAGCGGGGGGCTTGTGAGGCTACAGAGGAGG  2340
          ||||||||||||||||||||||||||||||||||||||||||||||||||||||||||||
2   2281  ATGGGCAGGGGCTAAAGGGGGTAAAGAGGGAGCGGGGGGCTTGTGAGGCTACAGAGGAGG  2340
```

```
1  2341  CTAGGAATCTAGCTTTTAGCTTAATGACCAGACACCGTCCTGAGTGTATTACTTTTCAAC  2400
         ||||||||||||||||||||||||||||||||||||||||||||||||||||||||||||
2  2341  CTAGGAATCTAGCTTTTAGCTTAATGACCAGACACCGTCCTGAGTGTATTACTTTTCAAC  2400

1  2401  AGATCAAGGATAATTGCGCTAATGAGCTTGATCTGCTGGCGCAGAAGTATTCCATAGAGC  2460
         ||||||||||||||||||||||||||||||||||||||||||||||||||||||||||||
2  2401  AGATCAAGGATAATTGCGCTAATGAGCTTGATCTGCTGGCGCAGAAGTATTCCATAGAGC  2460

1  2461  AGCTGACCACTTACTGGCTGCAGCCAGGGGATGATTTTGAGGAGGCTATTAGGGTATATG  2520
         ||||||||||||||||||||||||||||||||||||||||||||||||||||||||||||
2  2461  AGCTGACCACTTACTGGCTGCAGCCAGGGGATGATTTTGAGGAGGCTATTAGGGTATATG  2520

1  2521  CAAAGGTGGCACTTAGGCCAGATTGCAAGTACAAGATCAGCAAACTTGTAAATATCAGGA  2580
         ||||||||||||||||||||||||||||||||||||||||||||||||||||||||||||
2  2521  CAAAGGTGGCACTTAGGCCAGATTGCAAGTACAAGATCAGCAAACTTGTAAATATCAGGA  2580

1  2581  ATTGTTGCTACATTTCTGGGAACGGGGCCGAGGTGGAGATAGATACGGAGGATAGGGTGG  2640
         ||||||||||||||||||||||||||||||||||||||||||||||||||||||||||||
2  2581  ATTGTTGCTACATTTCTGGGAACGGGGCCGAGGTGGAGATAGATACGGAGGATAGGGTGG  2640

1  2641  CCTTTAGATGTAGCATGATAAATATGTGGCCGGGGGTGCTTGGCATGGACGGGGTGGTTA  2700
         ||||||||||||||||||||||||||||||||||||||||||||||||||||||||||||
2  2641  CCTTTAGATGTAGCATGATAAATATGTGGCCGGGGGTGCTTGGCATGGACGGGGTGGTTA  2700

1  2701  TTATGAATGTAAGGTTTACTGGCCCCAATTTTAGCGGTACGGTTTTCCTGGCCAATACCA  2760
         ||||||||||||||||||||||||||||||||||||||||||||||||||||||||||||
2  2701  TTATGAATGTAAGGTTTACTGGCCCCAATTTTAGCGGTACGGTTTTCCTGGCCAATACCA  2760

1  2761  ACCTTATCCTACACGGTGTAAGCTTCTATGGGTTTAACAATACCTGTGTGGAAGCCTGGA  2820
         ||||||||||||||||||||||||||||||||||||||||||||||||||||||||||||
2  2761  ACCTTATCCTACACGGTGTAAGCTTCTATGGGTTTAACAATACCTGTGTGGAAGCCTGGA  2820

1  2821  CCGATGTAAGGGTTCGGGGCTGTGCCTTTTACTGCTGCTGGAAGGGGGTGGTGTGTCGCC  2880
         ||||||||||||||||||||||||||||||||||||||||||||||||||||||||||||
2  2821  CCGATGTAAGGGTTCGGGGCTGTGCCTTTTACTGCTGCTGGAAGGGGGTGGTGTGTCGCC  2880

1  2881  CCAAAAGCAGGGCTTCAATTAAGAAATGCCTCTTTGAAAGGTGTACCTTGGGTATCCTGT  2940
         ||||||||||||||||||||||||||||||||||||||||||||||||||||||||||||
2  2881  CCAAAAGCAGGGCTTCAATTAAGAAATGCCTCTTTGAAAGGTGTACCTTGGGTATCCTGT  2940
```

```
1   2941   CTGAGGGTAACTCCAGGGTGCGCCACAATGTGGCCTCCGACTGTGGTTGCTTCATGCTAG   3000
           ||||||||||||||||||||||||||||||||||||||||||||||||||||||||||||
2   2941   CTGAGGGTAACTCCAGGGTGCGCCACAATGTGGCCTCCGACTGTGGTTGCTTCATGCTAG   3000

1   3001   TGAAAAGCGTGGCTGTGATTAAGCATAACATGGTATGTGGCAACTGCGAGGACAGGGCCT   3060
           ||||||||||||||||||||||||||||||||||||||||||||||||||||||||||||
2   3001   TGAAAAGCGTGGCTGTGATTAAGCATAACATGGTATGTGGCAACTGCGAGGACAGGGCCT   3060

1   3061   CTCAGATGCTGACCTGCTCGGACGGCAACTGTCACCTGCTGAAGACCATTCACGTAGCCA   3120
           ||||||||||||||||||||||||||||||||||||||||||||||||||||||||||||
2   3061   CTCAGATGCTGACCTGCTCGGACGGCAACTGTCACCTGCTGAAGACCATTCACGTAGCCA   3120

1   3121   GCCACTCTCGCAAGGCCTGGCCAGTGTTTGAGCATAACATACTGACCCGCTGTTCCTTGC   3180
           ||||||||||||||||||||||||||||||||||||||||||||||||||||||||||||
2   3121   GCCACTCTCGCAAGGCCTGGCCAGTGTTTGAGCATAACATACTGACCCGCTGTTCCTTGC   3180

1   3181   ATTTGGGTAACAGGAGGGGGGTGTTCCTACCTTACCAATGCAATTTGAGTCACACTAAGA   3240
           ||||||||||||||||||||||||||||||||||||||||||||||||||||||||||||
2   3181   ATTTGGGTAACAGGAGGGGGGTGTTCCTACCTTACCAATGCAATTTGAGTCACACTAAGA   3240

1   3241   TATTGCTTGAGCCCGAGAGCATGTCCAAGGTGAACCTGAACGGGGTGTTTGACATGACCA   3300
           ||||||||||||||||||||||||||||||||||||||||||||||||||||||||||||
2   3241   TATTGCTTGAGCCCGAGAGCATGTCCAAGGTGAACCTGAACGGGGTGTTTGACATGACCA   3300

1   3301   TGAAGATCTGGAAGGTGCTGAGGTACGATGAGACCCGCACCAGGTGCAGACCCTGCGAGT   3360
           ||||||||||||||||||||||||||||||||||||||||||||||||||||||||||||
2   3301   TGAAGATCTGGAAGGTGCTGAGGTACGATGAGACCCGCACCAGGTGCAGACCCTGCGAGT   3360

1   3361   GTGGCGGTAAACATATTAGGAACCAGCCTGTGATGCTGGATGTGACCGAGGAGCTGAGGC   3420
           ||||||||||||||||||||||||||||||||||||||||||||||||||||||||||||
2   3361   GTGGCGGTAAACATATTAGGAACCAGCCTGTGATGCTGGATGTGACCGAGGAGCTGAGGC   3420

1   3421   CCGATCACTTGGTGCTGGCCTGCACCCGCGCTGAGTTTGGCTCTAGCGATGAAGATACAG   3480
           ||||||||||||||||||||||||||||||||||||||||||||||||||||||||||||
2   3421   CCGATCACTTGGTGCTGGCCTGCACCCGCGCTGAGTTTGGCTCTAGCGATGAAGATACAG   3480

1   3481   ATTGAGGTACTGAAATGTGTGGGCGTGGCTTAAGGGTGGGAAAGAATATATAAGGTGGGG   3540
           ||||||||||||||||||||||||||||||||||||||||||||||||||||||||||||
2   3481   ATTGAGGTACTGAAATGTGTGGGCGTGGCTTAAGGGTGGGAAAGAATATATAAGGTGGGG   3540
```

```
1   3541  GTCTTATGTAGTTTTGTATCTGTTTTGCAGCAGCCGCCGCCGCCATGAGCACCAACTCGT  3600
          ||||||||||||||||||||||||||||||||||||||||||||||||||||||||||||
2   3541  GTCTTATGTAGTTTTGTATCTGTTTTGCAGCAGCCGCCGCCGCCATGAGCACCAACTCGT  3600

1   3601  TTGATGGAAGCATTGTGAGCTCATATTTGACAACGCGCATGCCCCCATGGGCCGGGGTGC  3660
          ||||||||||||||||||||||||||||||||||||||||||||||||||||||||||||
2   3601  TTGATGGAAGCATTGTGAGCTCATATTTGACAACGCGCATGCCCCCATGGGCCGGGGTGC  3660

1   3661  GTCAGAATGTGATGGGCTCCAGCATTGATGGTCGCCCCGTCCTGCCCGCAAACTCTACTA  3720
          ||||||||||||||||||||||||||||||||||||||||||||||||||||||||||||
2   3661  GTCAGAATGTGATGGGCTCCAGCATTGATGGTCGCCCCGTCCTGCCCGCAAACTCTACTA  3720

1   3721  CCTTGACCTACGAGACCGTGTCTGGAACGCCGTTGGAGACTGCAGCCTCCGCCGCCGCTT  3780
          ||||||||||||||||||||||||||||||||||||||||||||||||||||||||||||
2   3721  CCTTGACCTACGAGACCGTGTCTGGAACGCCGTTGGAGACTGCAGCCTCCGCCGCCGCTT  3780

1   3781  CAGCCGCTGCAGCCACCGCCCGCGGGATTGTGACTGACTTTGCTTTCCTGAGCCCGCTTG  3840
          ||||||||||||||||||||||||||||||||||||||||||||||||||||||||||||
2   3781  CAGCCGCTGCAGCCACCGCCCGCGGGATTGTGACTGACTTTGCTTTCCTGAGCCCGCTTG  3840

1   3841  CAAGCAGTGCAGCTTCCCGTTCATCCGCCCGCGATGACAAGTTGACGGCTCTTTTGGCAC  3900
          ||||||||||||||||||||||||||||||||||||||||||||||||||||||||||||
2   3841  CAAGCAGTGCAGCTTCCCGTTCATCCGCCCGCGATGACAAGTTGACGGCTCTTTTGGCAC  3900

1   3901  AATTGGATTCTTTGACCCGGGAACTTAATGTCGTTTCTCAGCAGCTGTTGGATCTGCGCC  3960
          ||||||||||||||||||||||||||||||||||||||||||||||||||||||||||||
2   3901  AATTGGATTCTTTGACCCGGGAACTTAATGTCGTTTCTCAGCAGCTGTTGGATCTGCGCC  3960

1   3961  AGCAGGTTTCTGCCCTGAAGGCTTCCTCCCCTCCCAATGCGGTTTAAAACATAAATAAAA  4020
          ||||||||||||||||||||||||||||||||||||||||||||||||||||||||||||
2   3961  AGCAGGTTTCTGCCCTGAAGGCTTCCTCCCCTCCCAATGCGGTTTAAAACATAAATAAAA  4020

1   4021  AACCAGACTCTGTTTGGATTTGGATCAAGCAAGTGTCTTGCTGTCTTTATTTAGGGGTTT  4080
          ||||||||||||||||||||||||||||||||||||||||||||||||||||||||||||
2   4021  AACCAGACTCTGTTTGGATTTGGATCAAGCAAGTGTCTTGCTGTCTTTATTTAGGGGTTT  4080

1   4081  TGCGCGCGCGGTAGGCCCGGGACCAGCGGTCTCGGTCGTTGAGGGTCCTGTGTATTTTTT  4140
          ||||||||||||||||||||||||||||||||||||||||||||||||||||||||||||
2   4081  TGCGCGCGCGGTAGGCCCGGGACCAGCGGTCTCGGTCGTTGAGGGTCCTGTGTATTTTTT  4140
```

```
1    4141  CCAGGACGTGGTAAAGGTGACTCTGGATGTTCAGATACATGGGCATAAGCCCGTCTCTGG  4200
           ||||||||||||||||||||||||||||||||||||||||||||||||||||||||||||
2    4141  CCAGGACGTGGTAAAGGTGACTCTGGATGTTCAGATACATGGGCATAAGCCCGTCTCTGG  4200

1    4201  GGTGGAGGTAGCACCACTGCAGAGCTTCATGCTGCGGGGTGGTGTTGTAGATGATCCAGT  4260
           ||||||||||||||||||||||||||||||||||||||||||||||||||||||||||||
2    4201  GGTGGAGGTAGCACCACTGCAGAGCTTCATGCTGCGGGGTGGTGTTGTAGATGATCCAGT  4260

1    4261  CGTAGCAGGAGCGCTGGGCGTGGTGCCTAAAAATGTCTTTCAGTAGCAAGCTGATTGCCA  4320
           ||||||||||||||||||||||||||||||||||||||||||||||||||||||||||||
2    4261  CGTAGCAGGAGCGCTGGGCGTGGTGCCTAAAAATGTCTTTCAGTAGCAAGCTGATTGCCA  4320

1    4321  GGGGCAGGCCCTTGGTGTAAGTGTTTACAAAGCGGTTAAGCTGGGATGGGTGCATACGTG  4380
           ||||||||||||||||||||||||||||||||||||||||||||||||||||||||||||
2    4321  GGGGCAGGCCCTTGGTGTAAGTGTTTACAAAGCGGTTAAGCTGGGATGGGTGCATACGTG  4380

1    4381  GGGATATGAGATGCATCTTGGACTGTATTTTTAGGTTGGCTATGTTCCCAGCCATATCCC  4440
           ||||||||||||||||||||||||||||||||||||||||||||||||||||||||||||
2    4381  GGGATATGAGATGCATCTTGGACTGTATTTTTAGGTTGGCTATGTTCCCAGCCATATCCC  4440

1    4441  TCCGGGGATTCATGTTGTGCAGAACCACCAGCACAGTGTATCCGGTGCACTTGGGAAATT  4500
           ||||||||||||||||||||||||||||||||||||||||||||||||||||||||||||
2    4441  TCCGGGGATTCATGTTGTGCAGAACCACCAGCACAGTGTATCCGGTGCACTTGGGAAATT  4500

1    4501  TGTCATGTAGCTTAGAAGGAAATGCGTGGAAGAACTTGGAGACGCCCTTGTGACCTCCAA  4560
           ||||||||||||||||||||||||||||||||||||||||||||||||||||||||||||
2    4501  TGTCATGTAGCTTAGAAGGAAATGCGTGGAAGAACTTGGAGACGCCCTTGTGACCTCCAA  4560

1    4561  GATTTTCCATGCATTCGTCCATAATGATGGCAATGGGCCCACGGGCGGCGGCCTGGGCGA  4620
           ||||||||||||||||||||||||||||||||||||||||||||||||||||||||||||
2    4561  GATTTTCCATGCATTCGTCCATAATGATGGCAATGGGCCCACGGGCGGCGGCCTGGGCGA  4620

1    4621  AGATATTTCTGGGATCACTAACGTCATAGTTGTGTTCCAGGATGAGATCGTCATAGGCCA  4680
           ||||||||||||||||||||||||||||||||||||||||||||||||||||||||||||
2    4621  AGATATTTCTGGGATCACTAACGTCATAGTTGTGTTCCAGGATGAGATCGTCATAGGCCA  4680

1    4681  TTTTTACAAAGCGCGGGCGGAGGGTGCCAGACTGCGGTATAATGGTTCCATCCGGCCCAG  4740
           ||||||||||||||||||||||||||||||||||||||||||||||||||||||||||||
2    4681  TTTTTACAAAGCGCGGGCGGAGGGTGCCAGACTGCGGTATAATGGTTCCATCCGGCCCAG  4740
```

```
1   4741  GGGCGTAGTTACCCTCACAGATTTGCATTTCCCACGCTTTGAGTTCAGATGGGGGGATCA  4800
          ||||||||||||||||||||||||||||||||||||||||||||||||||||||||||||
2   4741  GGGCGTAGTTACCCTCACAGATTTGCATTTCCCACGCTTTGAGTTCAGATGGGGGGATCA  4800

1   4801  TGTCTACCTGCGGGGCGATGAAGAAAACGGTTTCCGGGGTAGGGGAGATCAGCTGGGAAG  4860
          ||||||||||||||||||||||||||||||||||||||||||||||||||||||||||||
2   4801  TGTCTACCTGCGGGGCGATGAAGAAAACGGTTTCCGGGGTAGGGGAGATCAGCTGGGAAG  4860

1   4861  AAAGCAGGTTCCTGAGCAGCTGCGACTTACCGCAGCCGGTGGGCCCGTAAATCACACCTA  4920
          ||||||||||||||||||||||||||||||||||||||||||||||||||||||||||||
2   4861  AAAGCAGGTTCCTGAGCAGCTGCGACTTACCGCAGCCGGTGGGCCCGTAAATCACACCTA  4920

1   4921  TTACCGGCTGCAACTGGTAGTTAAGAGAGCTGCAGCTGCCGTCATCCCTGAGCAGGGGGG  4980
          ||||||||||||||||||||||||||||||||||||||||||||||||||||||||||||
2   4921  TTACCGGCTGCAACTGGTAGTTAAGAGAGCTGCAGCTGCCGTCATCCCTGAGCAGGGGGG  4980

1   4981  CCACTTCGTTAAGCATGTCCCTGACTCGCATGTTTTCCCTGACCAAATCCGCCAGAAGGC  5040
          ||||||||||||||||||||||||||||||||||||||||||||||||||||||||||||
2   4981  CCACTTCGTTAAGCATGTCCCTGACTCGCATGTTTTCCCTGACCAAATCCGCCAGAAGGC  5040

1   5041  GCTCGCCGCCCAGCGATAGCAGTTCTTGCAAGGAAGCAAAGTTTTTCAACGGTTTGAGAC  5100
          ||||||||||||||||||||||||||||||||||||||||||||||||||||||||||||
2   5041  GCTCGCCGCCCAGCGATAGCAGTTCTTGCAAGGAAGCAAAGTTTTTCAACGGTTTGAGAC  5100

1   5101  CGTCCGCCGTAGGCATGCTTTTGAGCGTTTGACCAAGCAGTTCCAGGCGGTCCCACAGCT  5160
          ||||||||||||||||||||||||||||||||||||||||||||||||||||||||||||
2   5101  CGTCCGCCGTAGGCATGCTTTTGAGCGTTTGACCAAGCAGTTCCAGGCGGTCCCACAGCT  5160

1   5161  CGGTCACCTGCTCTACGGCATCTCGATCCAGCATATCTCCTCGTTTCGCGGGTTGGGGCG  5220
          ||||||||||||||||||||||||||||||||||||||||||||||||||||||||||||
2   5161  CGGTCACCTGCTCTACGGCATCTCGATCCAGCATATCTCCTCGTTTCGCGGGTTGGGGCG  5220

1   5221  GCTTTCGCTGTACGGCAGTAGTCGGTGCTCGTCCAGACGGGCCAGGGTCATGTCTTTCCA  5280
          ||||||||||||||||||||||||||||||||||||||||||||||||||||||||||||
2   5221  GCTTTCGCTGTACGGCAGTAGTCGGTGCTCGTCCAGACGGGCCAGGGTCATGTCTTTCCA  5280

1   5281  CGGGCGCAGGGTCCTCGTCAGCGTAGTCTGGGTCACGGTGAAGGGGTGCGCTCCGGGCTG  5340
          ||||||||||||||||||||||||||||||||||||||||||||||||||||||||||||
2   5281  CGGGCGCAGGGTCCTCGTCAGCGTAGTCTGGGTCACGGTGAAGGGGTGCGCTCCGGGCTG  5340
```

```
1    5341  CGCGCTGGCCAGGGTGCGCTTGAGGCTGGTCCTGCTGGTGCTGAAGCGCTGCCGGTCTTC  5400
           ||||||||||||||||||||||||||||||||||||||||||||||||||||||||||||
2    5341  CGCGCTGGCCAGGGTGCGCTTGAGGCTGGTCCTGCTGGTGCTGAAGCGCTGCCGGTCTTC  5400

1    5401  GCCCTGCGCGTCGGCCAGGTAGCATTTGACCATGGTGTCATAGTCCAGCCCCTCCGCGGC  5460
           ||||||||||||||||||||||||||||||||||||||||||||||||||||||||||||
2    5401  GCCCTGCGCGTCGGCCAGGTAGCATTTGACCATGGTGTCATAGTCCAGCCCCTCCGCGGC  5460

1    5461  GTGGCCCTTGGCGCGCAGCTTGCCCTTGGAGGAGGCGCCGCACGAGGGGCAGTGCAGACT  5520
           ||||||||||||||||||||||||||||||||||||||||||||||||||||||||||||
2    5461  GTGGCCCTTGGCGCGCAGCTTGCCCTTGGAGGAGGCGCCGCACGAGGGGCAGTGCAGACT  5520

1    5521  TTTGAGGGCGTAGAGCTTGGGCGCGAGAAATACCGATTCCGGGGAGTAGGCATCCGCGCC  5580
           ||||||||||||||||||||||||||||||||||||||||||||||||||||||||||||
2    5521  TTTGAGGGCGTAGAGCTTGGGCGCGAGAAATACCGATTCCGGGGAGTAGGCATCCGCGCC  5580

1    5581  GCAGGCCCCGCAGACGGTCTCGCATTCCACGAGCCAGGTGAGCTCTGGCCGTTCGGGGTC  5640
           ||||||||||||||||||||||||||||||||||||||||||||||||||||||||||||
2    5581  GCAGGCCCCGCAGACGGTCTCGCATTCCACGAGCCAGGTGAGCTCTGGCCGTTCGGGGTC  5640

1    5641  AAAAACCAGGTTTCCCCCATGCTTTTTGATGCGTTTCTTACCTCTGGTTTCCATGAGCCG  5700
           ||||||||||||||||||||||||||||||||||||||||||||||||||||||||||||
2    5641  AAAAACCAGGTTTCCCCCATGCTTTTTGATGCGTTTCTTACCTCTGGTTTCCATGAGCCG  5700

1    5701  GTGTCCACGCTCGGTGACGAAAAGGCTGTCCGTGTCCCCGTATACAGACTTGAGAGGCCT  5760
           ||||||||||||||||||||||||||||||||||||||||||||||||||||||||||||
2    5701  GTGTCCACGCTCGGTGACGAAAAGGCTGTCCGTGTCCCCGTATACAGACTTGAGAGGCCT  5760

1    5761  GTCCTCGAGCGGTGTTCCGCGGTCCTCCTCGTATAGAAACTCGGACCACTCTGAGACAAA  5820
           ||||||||||||||||||||||||||||||||||||||||||||||||||||||||||||
2    5761  GTCCTCGAGCGGTGTTCCGCGGTCCTCCTCGTATAGAAACTCGGACCACTCTGAGACAAA  5820

1    5821  GGCTCGCGTCCAGGCCAGCACGAAGGAGGCTAAGTGGGAGGGGTAGCGGTCGTTGTCCAC  5880
           ||||||||||||||||||||||||||||||||||||||||||||||||||||||||||||
2    5821  GGCTCGCGTCCAGGCCAGCACGAAGGAGGCTAAGTGGGAGGGGTAGCGGTCGTTGTCCAC  5880

1    5881  TAGGGGGTCCACTCGCTCCAGGGTGTGAAGACACATGTCGCCCTCTTCGGCATCAAGGAA  5940
           ||||||||||||||||||||||||||||||||||||||||||||||||||||||||||||
2    5881  TAGGGGGTCCACTCGCTCCAGGGTGTGAAGACACATGTCGCCCTCTTCGGCATCAAGGAA  5940
```

```
1  5941  GGTGATTGGTTTGTAGGTGTAGGCCACGTGACCGGGTGTTCCTGAAGGGGGGCTATAAAA  6000
         ||||||||||||||||||||||||||||||||||||||||||||||||||||||||||||
2  5941  GGTGATTGGTTTGTAGGTGTAGGCCACGTGACCGGGTGTTCCTGAAGGGGGGCTATAAAA  6000

1  6001  GGGGGTGGGGGCGCGTTCGTCCTCACTCTCTTCCGCATCGCTGTCTGCGAGGGCCAGCTG  6060
         ||||||||||||||||||||||||||||||||||||||||||||||||||||||||||||
2  6001  GGGGGTGGGGGCGCGTTCGTCCTCACTCTCTTCCGCATCGCTGTCTGCGAGGGCCAGCTG  6060

1  6061  TTGGGGTGAGTACTCCCTCTGAAAAGCGGGCATGACTTCTGCGCTAAGATTGTCAGTTTC  6120
         ||||||||||||||||||||||||||||||||||||||||||||||||||||||||||||
2  6061  TTGGGGTGAGTACTCCCTCTGAAAAGCGGGCATGACTTCTGCGCTAAGATTGTCAGTTTC  6120

1  6121  CAAAAACGAGGAGGATTTGATATTCACCTGGCCCGCGGTGATGCCTTTGAGGGTGGCCGC  6180
         ||||||||||||||||||||||||||||||||||||||||||||||||||||||||||||
2  6121  CAAAAACGAGGAGGATTTGATATTCACCTGGCCCGCGGTGATGCCTTTGAGGGTGGCCGC  6180

1  6181  ATCCATCTGGTCAGAAAAGACAATCTTTTTGTTGTCAAGCTTGGTGGCAAACGACCCGTA  6240
         ||||||||||||||||||||||||||||||||||||||||||||||||||||||||||||
2  6181  ATCCATCTGGTCAGAAAAGACAATCTTTTTGTTGTCAAGCTTGGTGGCAAACGACCCGTA  6240

1  6241  GAGGGCGTTGGACAGCAACTTGGCGATGGAGCGCAGGGTTTGGTTTTTGTCGCGATCGGC  6300
         ||||||||||||||||||||||||||||||||||||||||||||||||||||||||||||
2  6241  GAGGGCGTTGGACAGCAACTTGGCGATGGAGCGCAGGGTTTGGTTTTTGTCGCGATCGGC  6300

1  6301  GCGCTCCTTGGCCGCGATGTTTAGCTGCACGTATTCGCGCGCAACGCACCGCCATTCGGG  6360
         ||||||||||||||||||||||||||||||||||||||||||||||||||||||||||||
2  6301  GCGCTCCTTGGCCGCGATGTTTAGCTGCACGTATTCGCGCGCAACGCACCGCCATTCGGG  6360

1  6361  AAAGACGGTGGTGCGCTCGTCGGGCACCAGGTGCACGCGCCAACCGCGGTTGTGCAGGGT  6420
         ||||||||||||||||||||||||||||||||||||||||||||||||||||||||||||
2  6361  AAAGACGGTGGTGCGCTCGTCGGGCACCAGGTGCACGCGCCAACCGCGGTTGTGCAGGGT  6420

1  6421  GACAAGGTCAACGCTGGTGGCTACCTCTCCGCGTAGGCGCTCGTTGGTCCAGCAGAGGCG  6480
         ||||||||||||||||||||||||||||||||||||||||||||||||||||||||||||
2  6421  GACAAGGTCAACGCTGGTGGCTACCTCTCCGCGTAGGCGCTCGTTGGTCCAGCAGAGGCG  6480

1  6481  GCCGCCCTTGCGCGAGCAGAATGGCGGTAGGGGGTCTAGCTGCGTCTCGTCCGGGGGGTC  6540
         ||||||||||||||||||||||||||||||||||||||||||||||||||||||||||||
2  6481  GCCGCCCTTGCGCGAGCAGAATGGCGGTAGGGGGTCTAGCTGCGTCTCGTCCGGGGGGTC  6540
```

```
1   6541  TGCGTCCACGGTAAAGACCCCGGGCAGCAGGCGCGCGTCGAAGTAGTCTATCTTGCATCC  6600
          ||||||||||||||||||||||||||||||||||||||||||||||||||||||||||||
2   6541  TGCGTCCACGGTAAAGACCCCGGGCAGCAGGCGCGCGTCGAAGTAGTCTATCTTGCATCC  6600

1   6601  TTGCAAGTCTAGCGCCTGCTGCCATGCGCGGGCGGCAAGCGCGCGCTCGTATGGGTTGAG  6660
          ||||||||||||||||||||||||||||||||||||||||||||||||||||||||||||
2   6601  TTGCAAGTCTAGCGCCTGCTGCCATGCGCGGGCGGCAAGCGCGCGCTCGTATGGGTTGAG  6660

1   6661  TGGGGGACCCCATGGCATGGGGTGGGTGAGCGCGGAGGCGTACATGCCGCAAATGTCGTA  6720
          ||||||||||||||||||||||||||||||||||||||||||||||||||||||||||||
2   6661  TGGGGGACCCCATGGCATGGGGTGGGTGAGCGCGGAGGCGTACATGCCGCAAATGTCGTA  6720

1   6721  AACGTAGAGGGGCTCTCTGAGTATTCCAAGATATGTAGGGTAGCATCTTCCACCGCGGAT  6780
          ||||||||||||||||||||||||||||||||||||||||||||||||||||||||||||
2   6721  AACGTAGAGGGGCTCTCTGAGTATTCCAAGATATGTAGGGTAGCATCTTCCACCGCGGAT  6780

1   6781  GCTGGCGCGCACGTAATCGTATAGTTCGTGCGAGGGAGCGAGGAGGTCGGGACCGAGGTT  6840
          ||||||||||||||||||||||||||||||||||||||||||||||||||||||||||||
2   6781  GCTGGCGCGCACGTAATCGTATAGTTCGTGCGAGGGAGCGAGGAGGTCGGGACCGAGGTT  6840

1   6841  GCTACGGGCGGGCTGCTCTGCTCGGAAGACTATCTGCCTGAAGATGGCATGTGAGTTGGA  6900
          ||||||||||||||||||||||||||||||||||||||||||||||||||||||||||||
2   6841  GCTACGGGCGGGCTGCTCTGCTCGGAAGACTATCTGCCTGAAGATGGCATGTGAGTTGGA  6900

1   6901  TGATATGGTTGGACGCTGGAAGACGTTGAAGCTGGCGTCTGTGAGACCTACCGCGTCACG  6960
          ||||||||||||||||||||||||||||||||||||||||||||||||||||||||||||
2   6901  TGATATGGTTGGACGCTGGAAGACGTTGAAGCTGGCGTCTGTGAGACCTACCGCGTCACG  6960

1   6961  CACGAAGGAGGCGTAGGAGTCGCGCAGCTTGTTGACCAGCTCGGCGGTGACCTGCACGTC  7020
          ||||||||||||||||||||||||||||||||||||||||||||||||||||||||||||
2   6961  CACGAAGGAGGCGTAGGAGTCGCGCAGCTTGTTGACCAGCTCGGCGGTGACCTGCACGTC  7020

1   7021  TAGGGCGCAGTAGTCCAGGGTTTCCTTGATGATGTCATACTTATCCTGTCCCTTTTTTTT  7080
          ||||||||||||||||||||||||||||||||||||||||||||||||||||||||||||
2   7021  TAGGGCGCAGTAGTCCAGGGTTTCCTTGATGATGTCATACTTATCCTGTCCCTTTTTTTT  7080

1   7081  CCACAGCTCGCGGTTGAGGACAAACTCTTCGCGGTCTTTCCAGTACTCTTGGATCGGAAA  7140
          ||||||||||||||||||||||||||||||||||||||||||||||||||||||||||||
2   7081  CCACAGCTCGCGGTTGAGGACAAACTCTTCGCGGTCTTTCCAGTACTCTTGGATCGGAAA  7140
```

```
1    7141  CCCGTCGGCCTCCGAACGGTAAGAGCCTAGCATGTAGAACTGGTTGACGGCCTGGTAGGC  7200
           ||||||||||||||||||||||||||||||||||||||||||||||||||||||||||||
2    7141  CCCGTCGGCCTCCGAACGGTAAGAGCCTAGCATGTAGAACTGGTTGACGGCCTGGTAGGC  7200

1    7201  GCAGCATCCCTTTTCTACGGGTAGCGCGTATGCCTGCGCGGCCTTCCGGAGCGAGGTGTG  7260
           ||||||||||||||||||||||||||||||||||||||||||||||||||||||||||||
2    7201  GCAGCATCCCTTTTCTACGGGTAGCGCGTATGCCTGCGCGGCCTTCCGGAGCGAGGTGTG  7260

1    7261  GGTGAGCGCAAAGGTGTCCCTGACCATGACTTTGAGGTACTGGTATTTGAAGTCAGTGTC  7320
           ||||||||||||||||||||||||||||||||||||||||||||||||||||||||||||
2    7261  GGTGAGCGCAAAGGTGTCCCTGACCATGACTTTGAGGTACTGGTATTTGAAGTCAGTGTC  7320

1    7321  GTCGCATCCGCCCTGCTCCCAGAGCAAAAAGTCCGTGCGCTTTTTGGAACGCGGATTTGG  7380
           ||||||||||||||||||||||||||||||||||||||||||||||||||||||||||||
2    7321  GTCGCATCCGCCCTGCTCCCAGAGCAAAAAGTCCGTGCGCTTTTTGGAACGCGGATTTGG  7380

1    7381  CAGGGCGAAGGTGACATCGTTGAAGAGTATCTTTCCCGCGCGAGGCATAAAGTTGCGTGT  7440
           ||||||||||||||||||||||||||||||||||||||||||||||||||||||||||||
2    7381  CAGGGCGAAGGTGACATCGTTGAAGAGTATCTTTCCCGCGCGAGGCATAAAGTTGCGTGT  7440

1    7441  GATGCGGAAGGGTCCCGGCACCTCGGAACGGTTGTTAATTACCTGGGCGGCGAGCACGAT  7500
           ||||||||||||||||||||||||||||||||||||||||||||||||||||||||||||
2    7441  GATGCGGAAGGGTCCCGGCACCTCGGAACGGTTGTTAATTACCTGGGCGGCGAGCACGAT  7500

1    7501  CTCGTCAAAGCCGTTGATGTTGTGGCCCACAATGTAAAGTTCCAAGAAGCGCGGGATGCC  7560
           ||||||||||||||||||||||||||||||||||||||||||||||||||||||||||||
2    7501  CTCGTCAAAGCCGTTGATGTTGTGGCCCACAATGTAAAGTTCCAAGAAGCGCGGGATGCC  7560

1    7561  CTTGATGGAAGGCAATTTTTTAAGTTCCTCGTAGGTGAGCTCTTCAGGGGAGCTGAGCCC  7620
           ||||||||||||||||||||||||||||||||||||||||||||||||||||||||||||
2    7561  CTTGATGGAAGGCAATTTTTTAAGTTCCTCGTAGGTGAGCTCTTCAGGGGAGCTGAGCCC  7620

1    7621  GTGCTCTGAAAGGGCCCAGTCTGCAAGATGAGGGTTGGAAGCGACGAATGAGCTCCACAG  7680
           ||||||||||||||||||||||||||||||||||||||||||||||||||||||||||||
2    7621  GTGCTCTGAAAGGGCCCAGTCTGCAAGATGAGGGTTGGAAGCGACGAATGAGCTCCACAG  7680

1    7681  GTCACGGGCCATTAGCATTTGCAGGTGGTCGCGAAAGGTCCTAAACTGGCGACCTATGGC  7740
           ||||||||||||||||||||||||||||||||||||||||||||||||||||||||||||
2    7681  GTCACGGGCCATTAGCATTTGCAGGTGGTCGCGAAAGGTCCTAAACTGGCGACCTATGGC  7740
```

```
1   7741   CATTTTTTCTGGGGTGATGCAGTAGAAGGTAAGCGGGTCTTGTTCCCAGCGGTCCCATCC   7800
           ||||||||||||||||||||||||||||||||||||||||||||||||||||||||||||
2   7741   CATTTTTTCTGGGGTGATGCAGTAGAAGGTAAGCGGGTCTTGTTCCCAGCGGTCCCATCC   7800

1   7801   AAGGTTCGCGGCTAGGTCTCGCGCGGCAGTCACTAGAGGCTCATCTCCGCCGAACTTCAT   7860
           ||||||||||||||||||||||||||||||||||||||||||||||||||||||||||||
2   7801   AAGGTTCGCGGCTAGGTCTCGCGCGGCAGTCACTAGAGGCTCATCTCCGCCGAACTTCAT   7860

1   7861   GACCAGCATGAAGGGCACGAGCTGCTTCCCAAAGGCCCCCATCCAAGTATAGGTCTCTAC   7920
           ||||||||||||||||||||||||||||||||||||||||||||||||||||||||||||
2   7861   GACCAGCATGAAGGGCACGAGCTGCTTCCCAAAGGCCCCCATCCAAGTATAGGTCTCTAC   7920

1   7921   ATCGTAGGTGACAAAGAGACGCTCGGTGCGAGGATGCGAGCCGATCGGGAAGAACTGGAT   7980
           ||||||||||||||||||||||||||||||||||||||||||||||||||||||||||||
2   7921   ATCGTAGGTGACAAAGAGACGCTCGGTGCGAGGATGCGAGCCGATCGGGAAGAACTGGAT   7980

1   7981   CTCCCGCCACCAATTGGAGGAGTGGCTATTGATGTGGTGAAAGTAGAAGTCCCTGCGACG   8040
           ||||||||||||||||||||||||||||||||||||||||||||||||||||||||||||
2   7981   CTCCCGCCACCAATTGGAGGAGTGGCTATTGATGTGGTGAAAGTAGAAGTCCCTGCGACG   8040

1   8041   GGCCGAACACTCGTGCTGGCTTTTGTAAAAACGTGCGCAGTACTGGCAGCGGTGCACGGG   8100
           ||||||||||||||||||||||||||||||||||||||||||||||||||||||||||||
2   8041   GGCCGAACACTCGTGCTGGCTTTTGTAAAAACGTGCGCAGTACTGGCAGCGGTGCACGGG   8100

1   8101   CTGTACATCCTGCACGAGGTTGACCTGACGACCGCGCACAAGGAAGCAGAGTGGGAATTT   8160
           ||||||||||||||||||||||||||||||||||||||||||||||||||||||||||||
2   8101   CTGTACATCCTGCACGAGGTTGACCTGACGACCGCGCACAAGGAAGCAGAGTGGGAATTT   8160

1   8161   GAGCCCCTCGCCTGGCGGGTTTGGCTGGTGGTCTTCTACTTCGGCTGCTTGTCCTTGACC   8220
           ||||||||||||||||||||||||||||||||||||||||||||||||||||||||||||
2   8161   GAGCCCCTCGCCTGGCGGGTTTGGCTGGTGGTCTTCTACTTCGGCTGCTTGTCCTTGACC   8220

1   8221   GTCTGGCTGCTCGAGGGGAGTTACGGTGGATCGGACCACCACGCCGCGCGAGCCCAAAGT   8280
           ||||||||||||||||||||||||||||||||||||||||||||||||||||||||||||
2   8221   GTCTGGCTGCTCGAGGGGAGTTACGGTGGATCGGACCACCACGCCGCGCGAGCCCAAAGT   8280

1   8281   CCAGATGTCCGCGCGCGGCGGTCGGAGCTTGATGACAACATCGCGCAGATGGGAGCTGTC   8340
           ||||||||||||||||||||||||||||||||||||||||||||||||||||||||||||
2   8281   CCAGATGTCCGCGCGCGGCGGTCGGAGCTTGATGACAACATCGCGCAGATGGGAGCTGTC   8340
```

```
1   8341  CATGGTCTGGAGCTCCCGCGGCGTCAGGTCAGGCGGGAGCTCCTGCAGGTTTACCTCGCA  8400
          ||||||||||||||||||||||||||||||||||||||||||||||||||||||||||||
2   8341  CATGGTCTGGAGCTCCCGCGGCGTCAGGTCAGGCGGGAGCTCCTGCAGGTTTACCTCGCA  8400

1   8401  TAGACGGGTCAGGGCGCGGGCTAGATCCAGGTGATACCTAATTTCCAGGGGCTGGTTGGT  8460
          ||||||||||||||||||||||||||||||||||||||||||||||||||||||||||||
2   8401  TAGACGGGTCAGGGCGCGGGCTAGATCCAGGTGATACCTAATTTCCAGGGGCTGGTTGGT  8460

1   8461  GGCGGCGTCGATGGCTTGCAAGAGGCCGCATCCCCGCGGCGCGACTACGGTACCGCGCGG  8520
          ||||||||||||||||||||||||||||||||||||||||||||||||||||||||||||
2   8461  GGCGGCGTCGATGGCTTGCAAGAGGCCGCATCCCCGCGGCGCGACTACGGTACCGCGCGG  8520

1   8521  CGGGCGGTGGGCCGCGGGGGTGTCCTTGGATGATGCATCTAAAAGCGGTGACGCGGGCGA  8580
          ||||||||||||||||||||||||||||||||||||||||||||||||||||||||||||
2   8521  CGGGCGGTGGGCCGCGGGGGTGTCCTTGGATGATGCATCTAAAAGCGGTGACGCGGGCGA  8580

1   8581  GCCCCCGGAGGTAGGGGGGGCTCCGGACCCGCCGGGAGAGGGGGCAGGGGCACGTCGGCG  8640
          ||||||||||||||||||||||||||||||||||||||||||||||||||||||||||||
2   8581  GCCCCCGGAGGTAGGGGGGGCTCCGGACCCGCCGGGAGAGGGGGCAGGGGCACGTCGGCG  8640

1   8641  CCGCGCGCGGGCAGGAGCTGGTGCTGCGCGCGTAGGTTGCTGGCGAACGCGACGACGCGG  8700
          ||||||||||||||||||||||||||||||||||||||||||||||||||||||||||||
2   8641  CCGCGCGCGGGCAGGAGCTGGTGCTGCGCGCGTAGGTTGCTGGCGAACGCGACGACGCGG  8700

1   8701  CGGTTGATCTCCTGAATCTGGCGCCTCTGCGTGAAGACGACGGGCCCGGTGAGCTTGAAC  8760
          ||||||||||||||||||||||||||||||||||||||||||||||||||||||||||||
2   8701  CGGTTGATCTCCTGAATCTGGCGCCTCTGCGTGAAGACGACGGGCCCGGTGAGCTTGAAC  8760

1   8761  CTGAAAGAGAGTTCGACAGAATCAATTTCGGTGTCGTTGACGGCGGCCTGGCGCAAAATC  8820
          ||||||||||||||||||||||||||||||||||||||||||||||||||||||||||||
2   8761  CTGAAAGAGAGTTCGACAGAATCAATTTCGGTGTCGTTGACGGCGGCCTGGCGCAAAATC  8820

1   8821  TCCTGCACGTCTCCTGAGTTGTCTTGATAGGCGATCTCGGCCATGAACTGCTCGATCTCT  8880
          ||||||||||||||||||||||||||||||||||||||||||||||||||||||||||||
2   8821  TCCTGCACGTCTCCTGAGTTGTCTTGATAGGCGATCTCGGCCATGAACTGCTCGATCTCT  8880

1   8881  TCCTCCTGGAGATCTCCGCGTCCGGCTCGCTCCACGGTGGCGGCGAGGTCGTTGGAAATG  8940
          ||||||||||||||||||||||||||||||||||||||||||||||||||||||||||||
2   8881  TCCTCCTGGAGATCTCCGCGTCCGGCTCGCTCCACGGTGGCGGCGAGGTCGTTGGAAATG  8940
```

```
1   8941  CGGGCCATGAGCTGCGAGAAGGCGTTGAGGCCTCCCTCGTTCCAGACGCGGCTGTAGACC  9000
          ||||||||||||||||||||||||||||||||||||||||||||||||||||||||||||
2   8941  CGGGCCATGAGCTGCGAGAAGGCGTTGAGGCCTCCCTCGTTCCAGACGCGGCTGTAGACC  9000

1   9001  ACGCCCCCTTCGGCATCGCGGGCGCGCATGACCACCTGCGCGAGATTGAGCTCCACGTGC  9060
          ||||||||||||||||||||||||||||||||||||||||||||||||||||||||||||
2   9001  ACGCCCCCTTCGGCATCGCGGGCGCGCATGACCACCTGCGCGAGATTGAGCTCCACGTGC  9060

1   9061  CGGGCGAAGACGGCGTAGTTTCGCAGGCGCTGAAAGAGGTAGTTGAGGGTGGTGGCGGTG  9120
          ||||||||||||||||||||||||||||||||||||||||||||||||||||||||||||
2   9061  CGGGCGAAGACGGCGTAGTTTCGCAGGCGCTGAAAGAGGTAGTTGAGGGTGGTGGCGGTG  9120

1   9121  TGTTCTGCCACGAAGAAGTACATAACCCAGCGTCGCAACGTGGATTCGTTGATATCCCCC  9180
          ||||||||||||||||||||||||||||||||||||||||||||||||||||||||||||
2   9121  TGTTCTGCCACGAAGAAGTACATAACCCAGCGTCGCAACGTGGATTCGTTGATATCCCCC  9180

1   9181  AAGGCCTCAAGGCGCTCCATGGCCTCGTAGAAGTCCACGGCGAAGTTGAAAAACTGGGAG  9240
          ||||||||||||||||||||||||||||||||||||||||||||||||||||||||||||
2   9181  AAGGCCTCAAGGCGCTCCATGGCCTCGTAGAAGTCCACGGCGAAGTTGAAAAACTGGGAG  9240

1   9241  TTGCGCGCCGACACGGTTAACTCCTCCTCCAGAAGACGGATGAGCTCGGCGACAGTGTCG  9300
          ||||||||||||||||||||||||||||||||||||||||||||||||||||||||||||
2   9241  TTGCGCGCCGACACGGTTAACTCCTCCTCCAGAAGACGGATGAGCTCGGCGACAGTGTCG  9300

1   9301  CGCACCTCGCGCTCAAAGGCTACAGGGGCCTCTTCTTCTTCTTCAATCTCCTCTTCCATA  9360
          ||||||||||||||||||||||||||||||||||||||||||||||||||||||||||||
2   9301  CGCACCTCGCGCTCAAAGGCTACAGGGGCCTCTTCTTCTTCTTCAATCTCCTCTTCCATA  9360

1   9361  AGGGCCTCCCCTTCTTCTTCTTCTGGCGGCGGTGGGGGAGGGGGGACACGGCGGCGACGA  9420
          ||||||||||||||||||||||||||||||||||||||||||||||||||||||||||||
2   9361  AGGGCCTCCCCTTCTTCTTCTTCTGGCGGCGGTGGGGGAGGGGGGACACGGCGGCGACGA  9420

1   9421  CGGCGCACCGGGAGGCGGTCGACAAAGCGCTCGATCATCTCCCCGCGGCGACGGCGCATG  9480
          ||||||||||||||||||||||||||||||||||||||||||||||||||||||||||||
2   9421  CGGCGCACCGGGAGGCGGTCGACAAAGCGCTCGATCATCTCCCCGCGGCGACGGCGCATG  9480

1   9481  GTCTCGGTGACGGCGCGGCCGTTCTCGCGGGGGCGCAGTTGGAAGACGCCGCCCGTCATG  9540
          ||||||||||||||||||||||||||||||||||||||||||||||||||||||||||||
2   9481  GTCTCGGTGACGGCGCGGCCGTTCTCGCGGGGGCGCAGTTGGAAGACGCCGCCCGTCATG  9540
```

```
1   9541    TCCCGGTTATGGGTTGGCGGGGGGCTGCCATGCGGCAGGGATACGGCGCTAACGATGCAT   9600
            ||||||||||||||||||||||||||||||||||||||||||||||||||||||||||||
2   9541    TCCCGGTTATGGGTTGGCGGGGGGCTGCCATGCGGCAGGGATACGGCGCTAACGATGCAT   9600

1   9601    CTCAACAATTGTTGTGTAGGTACTCCGCCGCCGAGGGACCTGAGCGAGTCCGCATCGACC   9660
            ||||||||||||||||||||||||||||||||||||||||||||||||||||||||||||
2   9601    CTCAACAATTGTTGTGTAGGTACTCCGCCGCCGAGGGACCTGAGCGAGTCCGCATCGACC   9660

1   9661    GGATCGGAAAACCTCTCGAGAAAGGCGTCTAACCAGTCACAGTCGCAAGGTAGGCTGAGC   9720
            ||||||||||||||||||||||||||||||||||||||||||||||||||||||||||||
2   9661    GGATCGGAAAACCTCTCGAGAAAGGCGTCTAACCAGTCACAGTCGCAAGGTAGGCTGAGC   9720

1   9721    ACCGTGGCGGGCGGCAGCGGGCGGCGGTCGGGGTTGTTTCTGGCGGAGGTGCTGCTGATG   9780
            ||||||||||||||||||||||||||||||||||||||||||||||||||||||||||||
2   9721    ACCGTGGCGGGCGGCAGCGGGCGGCGGTCGGGGTTGTTTCTGGCGGAGGTGCTGCTGATG   9780

1   9781    ATGTAATTAAAGTAGGCGGTCTTGAGACGGCGGATGGTCGACAGAAGCACCATGTCCTTG   9840
            ||||||||||||||||||||||||||||||||||||||||||||||||||||||||||||
2   9781    ATGTAATTAAAGTAGGCGGTCTTGAGACGGCGGATGGTCGACAGAAGCACCATGTCCTTG   9840

1   9841    GGTCCGGCCTGCTGAATGCGCAGGCGGTCGGCCATGCCCCAGGCTTCGTTTTGACATCGG   9900
            ||||||||||||||||||||||||||||||||||||||||||||||||||||||||||||
2   9841    GGTCCGGCCTGCTGAATGCGCAGGCGGTCGGCCATGCCCCAGGCTTCGTTTTGACATCGG   9900

1   9901    CGCAGGTCTTTGTAGTAGTCTTGCATGAGCCTTTCTACCGGCACTTCTTCTTCTCCTTCC   9960
            ||||||||||||||||||||||||||||||||||||||||||||||||||||||||||||
2   9901    CGCAGGTCTTTGTAGTAGTCTTGCATGAGCCTTTCTACCGGCACTTCTTCTTCTCCTTCC   9960

1   9961    TCTTGTCCTGCATCTCTTGCATCTATCGCTGCGGCGGCGGCGGAGTTTGGCCGTAGGTGG   10 020
            ||||||||||||||||||||||||||||||||||||||||||||||||||||||||||||
2   9961    TCTTGTCCTGCATCTCTTGCATCTATCGCTGCGGCGGCGGCGGAGTTTGGCCGTAGGTGG   10 020

1   10 021  CGCCCTCTTCCTCCCATGCGTGTGACCCCGAAGCCCCTCATCGGCTGAAGCAGGGCTAGG   10 080
            ||||||||||||||||||||||||||||||||||||||||||||||||||||||||||||
2   10 021  CGCCCTCTTCCTCCCATGCGTGTGACCCCGAAGCCCCTCATCGGCTGAAGCAGGGCTAGG   10 080

1   10 081  TCGGCGACAACGCGCTCGGCTAATATGGCCTGCTGCACCTGCGTGAGGGTAGACTGGAAG   10 140
            ||||||||||||||||||||||||||||||||||||||||||||||||||||||||||||
2   10 081  TCGGCGACAACGCGCTCGGCTAATATGGCCTGCTGCACCTGCGTGAGGGTAGACTGGAAG   10 140
```

```
1  10 141  TCATCCATGTCCACAAAGCGGTGGTATGCGCCCGTGTTGATGGTGTAAGTGCAGTTGGCC  10 200
           ||||||||||||||||||||||||||||||||||||||||||||||||||||||||||||
2  10 141  TCATCCATGTCCACAAAGCGGTGGTATGCGCCCGTGTTGATGGTGTAAGTGCAGTTGGCC  10 200

1  10 201  ATAACGGACCAGTTAACGGTCTGGTGACCCGGCTGCGAGAGCTCGGTGTACCTGAGACGC  10 260
           ||||||||||||||||||||||||||||||||||||||||||||||||||||||||||||
2  10 201  ATAACGGACCAGTTAACGGTCTGGTGACCCGGCTGCGAGAGCTCGGTGTACCTGAGACGC  10 260

1  10 261  GAGTAAGCCCTCGAGTCAAATACGTAGTCGTTGCAAGTCCGCACCAGGTACTGGTATCCC  10 320
           ||||||||||||||||||||||||||||||||||||||||||||||||||||||||||||
2  10 261  GAGTAAGCCCTCGAGTCAAATACGTAGTCGTTGCAAGTCCGCACCAGGTACTGGTATCCC  10 320

1  10 321  ACCAAAAAGTGCGGCGGCGGCTGGCGGTAGAGGGGCCAGCGTAGGGTGGCCGGGGCTCCG  10 380
           ||||||||||||||||||||||||||||||||||||||||||||||||||||||||||||
2  10 321  ACCAAAAAGTGCGGCGGCGGCTGGCGGTAGAGGGGCCAGCGTAGGGTGGCCGGGGCTCCG  10 380

1  10 381  GGGGCGAGATCTTCCAACATAAGGCGATGATATCCGTAGATGTACCTGGACATCCAGGTG  10 440
           ||||||||||||||||||||||||||||||||||||||||||||||||||||||||||||
2  10 381  GGGGCGAGATCTTCCAACATAAGGCGATGATATCCGTAGATGTACCTGGACATCCAGGTG  10 440

1  10 441  ATGCCGGCGGCGGTGGTGGAGGCGCGCGGAAAGTCGCGGACGCGGTTCCAGATGTTGCGC  10 500
           ||||||||||||||||||||||||||||||||||||||||||||||||||||||||||||
2  10 441  ATGCCGGCGGCGGTGGTGGAGGCGCGCGGAAAGTCGCGGACGCGGTTCCAGATGTTGCGC  10 500

1  10 501  AGCGGCAAAAAGTGCTCCATGGTCGGGACGCTCTGGCCGGTCAGGCGCGCGCAATCGTTG  10 560
           ||||||||||||||||||||||||||||||||||||||||||||||||||||||||||||
2  10 501  AGCGGCAAAAAGTGCTCCATGGTCGGGACGCTCTGGCCGGTCAGGCGCGCGCAATCGTTG  10 560

1  10 561  ACGCTCTAGACCGTGCAAAAGGAGAGCCTGTAAGCGGGCACTCTTCCGTGGTCTGGTGGA  10 620
           ||||||||||||||||||||||||||||||||||||||||||||||||||||||||||||
2  10 561  ACGCTCTAGACCGTGCAAAAGGAGAGCCTGTAAGCGGGCACTCTTCCGTGGTCTGGTGGA  10 620

1  10 621  TAAATTCGCAAGGGTATCATGGCGGACGACCGGGGTTCGAGCCCCGTATCCGGCCGTCCG  10 680
           ||||||||||||||||||||||||||||||||||||||||||||||||||||||||||||
2  10 621  TAAATTCGCAAGGGTATCATGGCGGACGACCGGGGTTCGAGCCCCGTATCCGGCCGTCCG  10 680

1  10 681  CCGTGATCCATGCGGTTACCGCCCGCGTGTCGAACCCAGGTGTGCGACGTCAGACAACGG  10 740
           ||||||||||||||||||||||||||||||||||||||||||||||||||||||||||||
2  10 681  CCGTGATCCATGCGGTTACCGCCCGCGTGTCGAACCCAGGTGTGCGACGTCAGACAACGG  10 740
```

```
1   10 741  GGGAGTGCTCCTTTTGGCTTCCTTCCAGGCGCGGCGGCTGCTGCGCTAGCTTTTTTGGCC  10 800
            ||||||||||||||||||||||||||||||||||||||||||||||||||||||||||||
2   10 741  GGGAGTGCTCCTTTTGGCTTCCTTCCAGGCGCGGCGGCTGCTGCGCTAGCTTTTTTGGCC  10 800

1   10 801  ACTGGCCGCGCGCAGCGTAAGCGGTTAGGCTGGAAAGCGAAAGCATTAAGTGGCTCGCTC  10 860
            ||||||||||||||||||||||||||||||||||||||||||||||||||||||||||||
2   10 801  ACTGGCCGCGCGCAGCGTAAGCGGTTAGGCTGGAAAGCGAAAGCATTAAGTGGCTCGCTC  10 860

1   10 861  CCTGTAGCCGGAGGGTTATTTTCCAAGGGTTGAGTCGCGGGACCCCCGGTTCGAGTCTCG  10 920
            ||||||||||||||||||||||||||||||||||||||||||||||||||||||||||||
2   10 861  CCTGTAGCCGGAGGGTTATTTTCCAAGGGTTGAGTCGCGGGACCCCCGGTTCGAGTCTCG  10 920

1   10 921  GACCGGCCGGACTGCGGCGAACGGGGGTTTGCCTCCCCGTCATGCAAGACCCCGCTTGCA  10 980
            ||||||||||||||||||||||||||||||||||||||||||||||||||||||||||||
2   10 921  GACCGGCCGGACTGCGGCGAACGGGGGTTTGCCTCCCCGTCATGCAAGACCCCGCTTGCA  10 980

1   10 981  AATTCCTCCGGAAACAGGGACGAGCCCCTTTTTTGCTTTTCCCAGATGCATCCGGTGCTG  11 040
            ||||||||||||||||||||||||||||||||||||||||||||||||||||||||||||
2   10 981  AATTCCTCCGGAAACAGGGACGAGCCCCTTTTTTGCTTTTCCCAGATGCATCCGGTGCTG  11 040

1   11 041  CGGCAGATGCGCCCCCCTCCTCAGCAGCGGCAAGAGCAAGAGCAGCGGCAGACATGCAGG  11 100
            ||||||||||||||||||||||||||||||||||||||||||||||||||||||||||||
2   11 041  CGGCAGATGCGCCCCCCTCCTCAGCAGCGGCAAGAGCAAGAGCAGCGGCAGACATGCAGG  11 100

1   11 101  GCACCCTCCCCTCCTCCTACCGCGTCAGGAGGGGCGACATCCGCGGTTGACGCGGCAGCA  11 160
            ||||||||||||||||||||||||||||||||||||||||||||||||||||||||||||
2   11 101  GCACCCTCCCCTCCTCCTACCGCGTCAGGAGGGGCGACATCCGCGGTTGACGCGGCAGCA  11 160

1   11 161  GATGGTGATTACGAACCCCCGCGGCGCCGGGCCCGGCACTACCTGGACTTGGAGGAGGGC  11 220
            ||||||||||||||||||||||||||||||||||||||||||||||||||||||||||||
2   11 161  GATGGTGATTACGAACCCCCGCGGCGCCGGGCCCGGCACTACCTGGACTTGGAGGAGGGC  11 220

1   11 221  GAGGGCCTGGCGCGGCTAGGAGCGCCCTCTCCTGAGCGGCACCCAAGGGTGCAGCTGAAG  11 280
            ||||||||||||||||||||||||||||||||||||||||||||||||||||||||||||
2   11 221  GAGGGCCTGGCGCGGCTAGGAGCGCCCTCTCCTGAGCGGCACCCAAGGGTGCAGCTGAAG  11 280

1   11 281  CGTGATACGCGTGAGGCGTACGTGCCGCGGCAGAACCTGTTTCGCGACCGCGAGGGAGAG  11 340
            ||||||||||||||||||||||||||||||||||||||||||||||||||||||||||||
2   11 281  CGTGATACGCGTGAGGCGTACGTGCCGCGGCAGAACCTGTTTCGCGACCGCGAGGGAGAG  11 340
```

```
1   11 341  GAGCCCGAGGAGATGCGGGATCGAAAGTTCCACGCAGGGCGCGAGCTGCGGCATGGCCTG  11 400
            ||||||||||||||||||||||||||||||||||||||||||||||||||||||||||||
2   11 341  GAGCCCGAGGAGATGCGGGATCGAAAGTTCCACGCAGGGCGCGAGCTGCGGCATGGCCTG  11 400

1   11 401  AATCGCGAGCGGTTGCTGCGCGAGGAGGACTTTGAGCCCGACGCGCGAACCGGGATTAGT  11 460
            ||||||||||||||||||||||||||||||||||||||||||||||||||||||||||||
2   11 401  AATCGCGAGCGGTTGCTGCGCGAGGAGGACTTTGAGCCCGACGCGCGAACCGGGATTAGT  11 460

1   11 461  CCCGCGCGCGCACACGTGGCGGCCGCCGACCTGGTAACCGCATACGAGCAGACGGTGAAC  11 520
            ||||||||||||||||||||||||||||||||||||||||||||||||||||||||||||
2   11 461  CCCGCGCGCGCACACGTGGCGGCCGCCGACCTGGTAACCGCATACGAGCAGACGGTGAAC  11 520

1   11 521  CAGGAGATTAACTTTCAAAAAAGCTTTAACAACCACGTGCGTACGCTTGTGGCGCGCGAG  11 580
            ||||||||||||||||||||||||||||||||||||||||||||||||||||||||||||
2   11 521  CAGGAGATTAACTTTCAAAAAAGCTTTAACAACCACGTGCGTACGCTTGTGGCGCGCGAG  11 580

1   11 581  GAGGTGGCTATAGGACTGATGCATCTGTGGGACTTTGTAAGCGCGCTGGAGCAAAACCCA  11 640
            ||||||||||||||||||||||||||||||||||||||||||||||||||||||||||||
2   11 581  GAGGTGGCTATAGGACTGATGCATCTGTGGGACTTTGTAAGCGCGCTGGAGCAAAACCCA  11 640

1   11 641  AATAGCAAGCCGCTCATGGCGCAGCTGTTCCTTATAGTGCAGCACAGCAGGGACAACGAG  11 700
            ||||||||||||||||||||||||||||||||||||||||||||||||||||||||||||
2   11 641  AATAGCAAGCCGCTCATGGCGCAGCTGTTCCTTATAGTGCAGCACAGCAGGGACAACGAG  11 700

1   11 701  GCATTCAGGGATGCGCTGCTAAACATAGTAGAGCCCGAGGGCCGCTGGCTGCTCGATTTG  11 760
            ||||||||||||||||||||||||||||||||||||||||||||||||||||||||||||
2   11 701  GCATTCAGGGATGCGCTGCTAAACATAGTAGAGCCCGAGGGCCGCTGGCTGCTCGATTTG  11 760

1   11 761  ATAAACATCCTGCAGAGCATAGTGGTGCAGGAGCGCAGCTTGAGCCTGGCTGACAAGGTG  11 820
            ||||||||||||||||||||||||||||||||||||||||||||||||||||||||||||
2   11 761  ATAAACATCCTGCAGAGCATAGTGGTGCAGGAGCGCAGCTTGAGCCTGGCTGACAAGGTG  11 820

1   11 821  GCCGCCATCAACTATTCCATGCTTAGCCTGGGCAAGTTTTACGCCCGCAAGATATACCAT  11 880
            ||||||||||||||||||||||||||||||||||||||||||||||||||||||||||||
2   11 821  GCCGCCATCAACTATTCCATGCTTAGCCTGGGCAAGTTTTACGCCCGCAAGATATACCAT  11 880

1   11 881  ACCCCTTACGTTCCCATAGACAAGGAGGTAAAGATCGAGGGGTTCTACATGCGCATGGCG  11 940
            ||||||||||||||||||||||||||||||||||||||||||||||||||||||||||||
2   11 881  ACCCCTTACGTTCCCATAGACAAGGAGGTAAAGATCGAGGGGTTCTACATGCGCATGGCG  11 940
```

```
1   11 941  CTGAAGGTGCTTACCTTGAGCGACGACCTGGGCGTTTATCGCAACGAGCGCATCCACAAG  12 000
            ||||||||||||||||||||||||||||||||||||||||||||||||||||||||||||
2   11 941  CTGAAGGTGCTTACCTTGAGCGACGACCTGGGCGTTTATCGCAACGAGCGCATCCACAAG  12 000

1   12 001  GCCGTGAGCGTGAGCCGGCGGCGCGAGCTCAGCGACCGCGAGCTGATGCACAGCCTGCAA  12 060
            ||||||||||||||||||||||||||||||||||||||||||||||||||||||||||||
2   12 001  GCCGTGAGCGTGAGCCGGCGGCGCGAGCTCAGCGACCGCGAGCTGATGCACAGCCTGCAA  12 060

1   12 061  AGGGCCCTGGCTGGCACGGGCAGCGGCGATAGAGAGGCCGAGTCCTACTTTGACGCGGGC  12 120
            ||||||||||||||||||||||||||||||||||||||||||||||||||||||||||||
2   12 061  AGGGCCCTGGCTGGCACGGGCAGCGGCGATAGAGAGGCCGAGTCCTACTTTGACGCGGGC  12 120

1   12 121  GCTGACCTGCGCTGGGCCCCAAGCCGACGCGCCCTGGAGGCAGCTGGGGCCGGACCTGGG  12 180
            ||||||||||||||||||||||||||||||||||||||||||||||||||||||||||||
2   12 121  GCTGACCTGCGCTGGGCCCCAAGCCGACGCGCCCTGGAGGCAGCTGGGGCCGGACCTGGG  12 180

1   12 181  CTGGCGGTGGCACCCGCGCGCGCTGGCAACGTCGGCGGCGTGGAGGAATATGACGAGGAC  12 240
            ||||||||||||||||||||||||||||||||||||||||||||||||||||||||||||
2   12 181  CTGGCGGTGGCACCCGCGCGCGCTGGCAACGTCGGCGGCGTGGAGGAATATGACGAGGAC  12 240

1   12 241  GATGAGTACGAGCCAGAGGACGGCGAGTACTAAGCGGTGATGTTTCTGATCAGATGATGC  12 300
            ||||||||||||||||||||||||||||||||||||||||||||||||||||||||||||
2   12 241  GATGAGTACGAGCCAGAGGACGGCGAGTACTAAGCGGTGATGTTTCTGATCAGATGATGC  12 300

1   12 301  AAGACGCAACGGACCCGGCGGTGCGGGCGGCGCTGCAGAGCCAGCCGTCCGGCCTTAACT  12 360
            ||||||||||||||||||||||||||||||||||||||||||||||||||||||||||||
2   12 301  AAGACGCAACGGACCCGGCGGTGCGGGCGGCGCTGCAGAGCCAGCCGTCCGGCCTTAACT  12 360

1   12 361  CCACGGACGACTGGCGCCAGGTCATGGACCGCATCATGTCGCTGACTGCGCGCAATCCTG  12 420
            ||||||||||||||||||||||||||||||||||||||||||||||||||||||||||||
2   12 361  CCACGGACGACTGGCGCCAGGTCATGGACCGCATCATGTCGCTGACTGCGCGCAATCCTG  12 420

1   12 421  ACGCGTTCCGGCAGCAGCCGCAGGCCAACCGGCTCTCCGCAATTCTGGAAGCGGTGGTCC  12 480
            ||||||||||||||||||||||||||||||||||||||||||||||||||||||||||||
2   12 421  ACGCGTTCCGGCAGCAGCCGCAGGCCAACCGGCTCTCCGCAATTCTGGAAGCGGTGGTCC  12 480

1   12 481  CGGCGCGCGCAAACCCCACGCACGAGAAGGTGCTGGCGATCGTAAACGCGCTGGCCGAAA  12 540
            ||||||||||||||||||||||||||||||||||||||||||||||||||||||||||||
2   12 481  CGGCGCGCGCAAACCCCACGCACGAGAAGGTGCTGGCGATCGTAAACGCGCTGGCCGAAA  12 540
```

```
1   12 541  ACAGGGCCATCCGGCCCGACGAGGCCGGCCTGGTCTACGACGCGCTGCTTCAGCGCGTGG  12 600
            ||||||||||||||||||||||||||||||||||||||||||||||||||||||||||||
2   12 541  ACAGGGCCATCCGGCCCGACGAGGCCGGCCTGGTCTACGACGCGCTGCTTCAGCGCGTGG  12 600

1   12 601  CTCGTTACAACAGCGGCAACGTGCAGACCAACCTGGACCGGCTGGTGGGGGATGTGCGCG  12 660
            ||||||||||||||||||||||||||||||||||||||||||||||||||||||||||||
2   12 601  CTCGTTACAACAGCGGCAACGTGCAGACCAACCTGGACCGGCTGGTGGGGGATGTGCGCG  12 660

1   12 661  AGGCCGTGGCGCAGCGTGAGCGCGCGCAGCAGCAGGGCAACCTGGGCTCCATGGTTGCAC  12 720
            ||||||||||||||||||||||||||||||||||||||||||||||||||||||||||||
2   12 661  AGGCCGTGGCGCAGCGTGAGCGCGCGCAGCAGCAGGGCAACCTGGGCTCCATGGTTGCAC  12 720

1   12 721  TAAACGCCTTCCTGAGTACACAGCCCGCCAACGTGCCGCGGGGACAGGAGGACTACACCA  12 780
            ||||||||||||||||||||||||||||||||||||||||||||||||||||||||||||
2   12 721  TAAACGCCTTCCTGAGTACACAGCCCGCCAACGTGCCGCGGGGACAGGAGGACTACACCA  12 780

1   12 781  ACTTTGTGAGCGCACTGCGGCTAATGGTGACTGAGACACCGCAAAGTGAGGTGTACCAGT  12 840
            ||||||||||||||||||||||||||||||||||||||||||||||||||||||||||||
2   12 781  ACTTTGTGAGCGCACTGCGGCTAATGGTGACTGAGACACCGCAAAGTGAGGTGTACCAGT  12 840

1   12 841  CTGGGCCAGACTATTTTTTCCAGACCAGTAGACAAGGCCTGCAGACCGTAAACCTGAGCC  12 900
            ||||||||||||||||||||||||||||||||||||||||||||||||||||||||||||
2   12 841  CTGGGCCAGACTATTTTTTCCAGACCAGTAGACAAGGCCTGCAGACCGTAAACCTGAGCC  12 900

1   12 901  AGGCTTTCAAAAACTTGCAGGGGCTGTGGGGGGTGCGGGCTCCCACAGGCGACCGCGCGA  12 960
            ||||||||||||||||||||||||||||||||||||||||||||||||||||||||||||
2   12 901  AGGCTTTCAAAAACTTGCAGGGGCTGTGGGGGGTGCGGGCTCCCACAGGCGACCGCGCGA  12 960

1   12 961  CCGTGTCTAGCTTGCTGACGCCCAACTCGCGCCTGTTGCTGCTGCTAATAGCGCCCTTCA  13 020
            ||||||||||||||||||||||||||||||||||||||||||||||||||||||||||||
2   12 961  CCGTGTCTAGCTTGCTGACGCCCAACTCGCGCCTGTTGCTGCTGCTAATAGCGCCCTTCA  13 020

1   13 021  CGGACAGTGGCAGCGTGTCCCGGGACACATACCTAGGTCACTTGCTGACACTGTACCGCG  13 080
            ||||||||||||||||||||||||||||||||||||||||||||||||||||||||||||
2   13 021  CGGACAGTGGCAGCGTGTCCCGGGACACATACCTAGGTCACTTGCTGACACTGTACCGCG  13 080

1   13 081  AGGCCATAGGTCAGGCGCATGTGGACGAGCATACTTTCCAGGAGATTACAAGTGTCAGCC  13 140
            ||||||||||||||||||||||||||||||||||||||||||||||||||||||||||||
2   13 081  AGGCCATAGGTCAGGCGCATGTGGACGAGCATACTTTCCAGGAGATTACAAGTGTCAGCC  13 140
```

```
1   13 141  GCGCGCTGGGGCAGGAGGACACGGGCAGCCTGGAGGCAACCCTAAACTACCTGCTGACCA  13 200
            ||||||||||||||||||||||||||||||||||||||||||||||||||||||||||||
2   13 141  GCGCGCTGGGGCAGGAGGACACGGGCAGCCTGGAGGCAACCCTAAACTACCTGCTGACCA  13 200

1   13 201  ACCGGCGGCAGAAGATCCCCTCGTTGCACAGTTTAAACAGCGAGGAGGAGCGCATTTTGC  13 260
            ||||||||||||||||||||||||||||||||||||||||||||||||||||||||||||
2   13 201  ACCGGCGGCAGAAGATCCCCTCGTTGCACAGTTTAAACAGCGAGGAGGAGCGCATTTTGC  13 260

1   13 261  GCTACGTGCAGCAGAGCGTGAGCCTTAACCTGATGCGCGACGGGGTAACGCCCAGCGTGG  13 320
            ||||||||||||||||||||||||||||||||||||||||||||||||||||||||||||
2   13 261  GCTACGTGCAGCAGAGCGTGAGCCTTAACCTGATGCGCGACGGGGTAACGCCCAGCGTGG  13 320

1   13 321  CGCTGGACATGACCGCGCGCAACATGGAACCGGGCATGTATGCCTCAAACCGGCCGTTTA  13 380
            ||||||||||||||||||||||||||||||||||||||||||||||||||||||||||||
2   13 321  CGCTGGACATGACCGCGCGCAACATGGAACCGGGCATGTATGCCTCAAACCGGCCGTTTA  13 380

1   13 381  TCAACCGCCTAATGGACTACTTGCATCGCGCGGCCGCCGTGAACCCCGAGTATTTCACCA  13 440
            ||||||||||||||||||||||||||||||||||||||||||||||||||||||||||||
2   13 381  TCAACCGCCTAATGGACTACTTGCATCGCGCGGCCGCCGTGAACCCCGAGTATTTCACCA  13 440

1   13 441  ATGCCATCTTGAACCCGCACTGGCTACCGCCCCCTGGTTTCTACACCGGGGGATTCGAGG  13 500
            ||||||||||||||||||||||||||||||||||||||||||||||||||||||||||||
2   13 441  ATGCCATCTTGAACCCGCACTGGCTACCGCCCCCTGGTTTCTACACCGGGGGATTCGAGG  13 500

1   13 501  TGCCCGAGGGTAACGATGGATTCCTCTGGGACGACATAGACGACAGCGTGTTTTCCCCGC  13 560
            ||||||||||||||||||||||||||||||||||||||||||||||||||||||||||||
2   13 501  TGCCCGAGGGTAACGATGGATTCCTCTGGGACGACATAGACGACAGCGTGTTTTCCCCGC  13 560

1   13 561  AACCGCAGACCCTGCTAGAGTTGCAACAGCGCGAGCAGGCAGAGGCGGCGCTGCGAAAGG  13 620
            ||||||||||||||||||||||||||||||||||||||||||||||||||||||||||||
2   13 561  AACCGCAGACCCTGCTAGAGTTGCAACAGCGCGAGCAGGCAGAGGCGGCGCTGCGAAAGG  13 620

1   13 621  AAAGCTTCCGCAGGCCAAGCAGCTTGTCCGATCTAGGCGCTGCGGCCCCGCGGTCAGATG  13 680
            ||||||||||||||||||||||||||||||||||||||||||||||||||||||||||||
2   13 621  AAAGCTTCCGCAGGCCAAGCAGCTTGTCCGATCTAGGCGCTGCGGCCCCGCGGTCAGATG  13 680

1   13 681  CTAGTAGCCCATTTCCAAGCTTGATAGGGTCTCTTACCAGCACTCGCACCACCCGCCCGC  13 740
            ||||||||||||||||||||||||||||||||||||||||||||||||||||||||||||
2   13 681  CTAGTAGCCCATTTCCAAGCTTGATAGGGTCTCTTACCAGCACTCGCACCACCCGCCCGC  13 740
```

```
1   13 741   GCCTGCTGGGCGAGGAGGAGTACCTAAACAACTCGCTGCTGCAGCCGCAGCGCGAAAAAA   13 800
             ||||||||||||||||||||||||||||||||||||||||||||||||||||||||||||
2   13 741   GCCTGCTGGGCGAGGAGGAGTACCTAAACAACTCGCTGCTGCAGCCGCAGCGCGAAAAAA   13 800

1   13 801   ACCTGCCTCCGGCATTTCCCAACAACGGGATAGAGAGCCTAGTGGACAAGATGAGTAGAT   13 860
             ||||||||||||||||||||||||||||||||||||||||||||||||||||||||||||
2   13 801   ACCTGCCTCCGGCATTTCCCAACAACGGGATAGAGAGCCTAGTGGACAAGATGAGTAGAT   13 860

1   13 861   GGAAGACGTACGCGCAGGAGCACAGGGACGTGCCAGGCCCGCGCCCGCCCACCCGTCGTC   13 920
             ||||||||||||||||||||||||||||||||||||||||||||||||||||||||||||
2   13 861   GGAAGACGTACGCGCAGGAGCACAGGGACGTGCCAGGCCCGCGCCCGCCCACCCGTCGTC   13 920

1   13 921   AAAGGCACGACCGTCAGCGGGGTCTGGTGTGGGAGGACGATGACTCGGCAGACGACAGCA   13 980
             ||||||||||||||||||||||||||||||||||||||||||||||||||||||||||||
2   13 921   AAAGGCACGACCGTCAGCGGGGTCTGGTGTGGGAGGACGATGACTCGGCAGACGACAGCA   13 980

1   13 981   GCGTCCTGGATTTGGGAGGGAGTGGCAACCCGTTTGCGCACCTTCGCCCCAGGCTGGGGA   14 040
             ||||||||||||||||||||||||||||||||||||||||||||||||||||||||||||
2   13 981   GCGTCCTGGATTTGGGAGGGAGTGGCAACCCGTTTGCGCACCTTCGCCCCAGGCTGGGGA   14 040

1   14 041   GAATGTTTTAAAAAAAAAAAAAGCATGATGCAAAATAAAAAACTCACCAAGGCCATGGCA   14 100
             ||||||||||||||||||||||||||||||||||||||||||||||||||||||||||||
2   14 041   GAATGTTTTAAAAAAAAAAAAAGCATGATGCAAAATAAAAAACTCACCAAGGCCATGGCA   14 100

1   14 101   CCGAGCGTTGGTTTTCTTGTATTCCCCTTAGTATGCGGCGCGCGGCGATGTATGAGGAAG   14 160
             ||||||||||||||||||||||||||||||||||||||||||||||||||||||||||||
2   14 101   CCGAGCGTTGGTTTTCTTGTATTCCCCTTAGTATGCGGCGCGCGGCGATGTATGAGGAAG   14 160

1   14 161   GTCCTCCTCCCTCCTACGAGAGTGTGGTGAGCGCGGCGCCAGTGGCGGCGGCGCTGGGTT   14 220
             ||||||||||||||||||||||||||||||||||||||||||||||||||||||||||||
2   14 161   GTCCTCCTCCCTCCTACGAGAGTGTGGTGAGCGCGGCGCCAGTGGCGGCGGCGCTGGGTT   14 220

1   14 221   CTCCCTTCGATGCTCCCCTGGACCCGCCGTTTGTGCCTCCGCGGTACCTGCGGCCTACCG   14 280
             ||||||||||||||||||||||||||||||||||||||||||||||||||||||||||||
2   14 221   CTCCCTTCGATGCTCCCCTGGACCCGCCGTTTGTGCCTCCGCGGTACCTGCGGCCTACCG   14 280

1   14 281   GGGGGAGAAACAGCATCCGTTACTCTGAGTTGGCACCCCTATTCGACACCACCCGTGTGT   14 340
             ||||||||||||||||||||||||||||||||||||||||||||||||||||||||||||
2   14 281   GGGGGAGAAACAGCATCCGTTACTCTGAGTTGGCACCCCTATTCGACACCACCCGTGTGT   14 340
```

```
1   14 341  ACCTGGTGGACAACAAGTCAACGGATGTGGCATCCCTGAACTACCAGAACGACCACAGCA  14 400
            ||||||||||||||||||||||||||||||||||||||||||||||||||||||||||||
2   14 341  ACCTGGTGGACAACAAGTCAACGGATGTGGCATCCCTGAACTACCAGAACGACCACAGCA  14 400

1   14 401  ACTTTCTGACCACGGTCATTCAAAACAATGACTACAGCCCGGGGGAGGCAAGCACACAGA  14 460
            ||||||||||||||||||||||||||||||||||||||||||||||||||||||||||||
2   14 401  ACTTTCTGACCACGGTCATTCAAAACAATGACTACAGCCCGGGGGAGGCAAGCACACAGA  14 460

1   14 461  CCATCAATCTTGACGACCGGTCGCACTGGGGCGGCGACCTGAAAACCATCCTGCATACCA  14 520
            ||||||||||||||||||||||||||||||||||||||||||||||||||||||||||||
2   14 461  CCATCAATCTTGACGACCGGTCGCACTGGGGCGGCGACCTGAAAACCATCCTGCATACCA  14 520

1   14 521  ACATGCCAAATGTGAACGAGTTCATGTTTACCAATAAGTTTAAGGCGCGGGTGATGGTGT  14 580
            ||||||||||||||||||||||||||||||||||||||||||||||||||||||||||||
2   14 521  ACATGCCAAATGTGAACGAGTTCATGTTTACCAATAAGTTTAAGGCGCGGGTGATGGTGT  14 580

1   14 581  CGCGCTTGCCTACTAAGGACAATCAGGTGGAGCTGAAATACGAGTGGGTGGAGTTCACGC  14 640
            ||||||||||||||||||||||||||||||||||||||||||||||||||||||||||||
2   14 581  CGCGCTTGCCTACTAAGGACAATCAGGTGGAGCTGAAATACGAGTGGGTGGAGTTCACGC  14 640

1   14 641  TGCCCGAGGGCAACTACTCCGAGACCATGACCATAGACCTTATGAACAACGCGATCGTGG  14 700
            ||||||||||||||||||||||||||||||||||||||||||||||||||||||||||||
2   14 641  TGCCCGAGGGCAACTACTCCGAGACCATGACCATAGACCTTATGAACAACGCGATCGTGG  14 700

1   14 701  AGCACTACTTGAAAGTGGGCAGACAGAACGGGGTTCTGGAAAGCGACATCGGGGTAAAGT  14 760
            ||||||||||||||||||||||||||||||||||||||||||||||||||||||||||||
2   14 701  AGCACTACTTGAAAGTGGGCAGACAGAACGGGGTTCTGGAAAGCGACATCGGGGTAAAGT  14 760

1   14 761  TTGACACCCGCAACTTCAGACTGGGGTTTGACCCCGTCACTGGTCTTGTCATGCCTGGGG  14 820
            ||||||||||||||||||||||||||||||||||||||||||||||||||||||||||||
2   14 761  TTGACACCCGCAACTTCAGACTGGGGTTTGACCCCGTCACTGGTCTTGTCATGCCTGGGG  14 820

1   14 821  TATATACAAACGAAGCCTTCCATCCAGACATCATTTTGCTGCCAGGATGCGGGGTGGACT  14 880
            ||||||||||||||||||||||||||||||||||||||||||||||||||||||||||||
2   14 821  TATATACAAACGAAGCCTTCCATCCAGACATCATTTTGCTGCCAGGATGCGGGGTGGACT  14 880

1   14 881  TCACCCACAGCCGCCTGAGCAACTTGTTGGGCATCCGCAAGCGGCAACCCTTCCAGGAGG  14 940
            ||||||||||||||||||||||||||||||||||||||||||||||||||||||||||||
2   14 881  TCACCCACAGCCGCCTGAGCAACTTGTTGGGCATCCGCAAGCGGCAACCCTTCCAGGAGG  14 940
```

```
1   14 941  GCTTTAGGATCACCTACGATGATCTGGAGGGTGGTAACATTCCCGCACTGTTGGATGTGG  15 000
            ||||||||||||||||||||||||||||||||||||||||||||||||||||||||||||
2   14 941  GCTTTAGGATCACCTACGATGATCTGGAGGGTGGTAACATTCCCGCACTGTTGGATGTGG  15 000

1   15 001  ACGCCTACCAGGCGAGCTTGAAAGATGACACCGAACAGGGCGGGGGTGGCGCAGGCGGCA  15 060
            ||||||||||||||||||||||||||||||||||||||||||||||||||||||||||||
2   15 001  ACGCCTACCAGGCGAGCTTGAAAGATGACACCGAACAGGGCGGGGGTGGCGCAGGCGGCA  15 060

1   15 061  GCAACAGCAGTGGCAGCGGCGCGGAAGAGAACTCCAACGCGGCAGCCGCGGCAATGCAGC  15 120
            ||||||||||||||||||||||||||||||||||||||||||||||||||||||||||||
2   15 061  GCAACAGCAGTGGCAGCGGCGCGGAAGAGAACTCCAACGCGGCAGCCGCGGCAATGCAGC  15 120

1   15 121  CGGTGGAGGACATGAACGATCATGCCATTCGCGGCGACACCTTTGCCACACGGGCTGAGG  15 180
            ||||||||||||||||||||||||||||||||||||||||||||||||||||||||||||
2   15 121  CGGTGGAGGACATGAACGATCATGCCATTCGCGGCGACACCTTTGCCACACGGGCTGAGG  15 180

1   15 181  AGAAGCGCGCTGAGGCCGAAGCAGCGGCCGAAGCTGCCGCCCCCGCTGCGCAACCCGAGG  15 240
            ||||||||||||||||||||||||||||||||||||||||||||||||||||||||||||
2   15 181  AGAAGCGCGCTGAGGCCGAAGCAGCGGCCGAAGCTGCCGCCCCCGCTGCGCAACCCGAGG  15 240

1   15 241  TCGAGAAGCCTCAGAAGAAACCGGTGATCAAACCCCTGACAGAGGACAGCAAGAAACGCA  15 300
            ||||||||||||||||||||||||||||||||||||||||||||||||||||||||||||
2   15 241  TCGAGAAGCCTCAGAAGAAACCGGTGATCAAACCCCTGACAGAGGACAGCAAGAAACGCA  15 300

1   15 301  GTTACAACCTAATAAGCAATGACAGCACCTTCACCCAGTACCGCAGCTGGTACCTTGCAT  15 360
            ||||||||||||||||||||||||||||||||||||||||||||||||||||||||||||
2   15 301  GTTACAACCTAATAAGCAATGACAGCACCTTCACCCAGTACCGCAGCTGGTACCTTGCAT  15 360

1   15 361  ACAACTACGGCGACCCTCAGACCGGAATCCGCTCATGGACCCTGCTTTGCACTCCTGACG  15 420
            ||||||||||||||||||||||||||||||||||||||||||||||||||||||||||||
2   15 361  ACAACTACGGCGACCCTCAGACCGGAATCCGCTCATGGACCCTGCTTTGCACTCCTGACG  15 420

1   15 421  TAACCTGCGGCTCGGAGCAGGTCTACTGGTCGTTGCCAGACATGATGCAAGACCCCGTGA  15 480
            ||||||||||||||||||||||||||||||||||||||||||||||||||||||||||||
2   15 421  TAACCTGCGGCTCGGAGCAGGTCTACTGGTCGTTGCCAGACATGATGCAAGACCCCGTGA  15 480

1   15 481  CCTTCCGCTCCACGCGCCAGATCAGCAACTTTCCGGTGGTGGGCGCCGAGCTGTTGCCCG  15 540
            ||||||||||||||||||||||||||||||||||||||||||||||||||||||||||||
2   15 481  CCTTCCGCTCCACGCGCCAGATCAGCAACTTTCCGGTGGTGGGCGCCGAGCTGTTGCCCG  15 540
```

```
1   15 541  TGCACTCCAAGAGCTTCTACAACGACCAGGCCGTCTACTCCCAACTCATCCGCCAGTTTA  15 600
            ||||||||||||||||||||||||||||||||||||||||||||||||||||||||||||
2   15 541  TGCACTCCAAGAGCTTCTACAACGACCAGGCCGTCTACTCCCAACTCATCCGCCAGTTTA  15 600

1   15 601  CCTCTCTGACCCACGTGTTCAATCGCTTTCCCGAGAACCAGATTTTGGCGCGCCCGCCAG  15 660
            ||||||||||||||||||||||||||||||||||||||||||||||||||||||||||||
2   15 601  CCTCTCTGACCCACGTGTTCAATCGCTTTCCCGAGAACCAGATTTTGGCGCGCCCGCCAG  15 660

1   15 661  CCCCCACCATCACCACCGTCAGTGAAAACGTTCCTGCTCTCACAGATCACGGGACGCTAC  15 720
            ||||||||||||||||||||||||||||||||||||||||||||||||||||||||||||
2   15 661  CCCCCACCATCACCACCGTCAGTGAAAACGTTCCTGCTCTCACAGATCACGGGACGCTAC  15 720

1   15 721  CGCTGCGCAACAGCATCGGAGGAGTCCAGCGAGTGACCATTACTGACGCCAGACGCCGCA  15 780
            ||||||||||||||||||||||||||||||||||||||||||||||||||||||||||||
2   15 721  CGCTGCGCAACAGCATCGGAGGAGTCCAGCGAGTGACCATTACTGACGCCAGACGCCGCA  15 780

1   15 781  CCTGCCCCTACGTTTACAAGGCCCTGGGCATAGTCTCGCCGCGCGTCCTATCGAGCCGCA  15 840
            ||||||||||||||||||||||||||||||||||||||||||||||||||||||||||||
2   15 781  CCTGCCCCTACGTTTACAAGGCCCTGGGCATAGTCTCGCCGCGCGTCCTATCGAGCCGCA  15 840

1   15 841  CTTTTTGAGCAAGCATGTCCATCCTTATATCGCCCAGCAATAACACAGGCTGGGGCCTGC  15 900
            ||||||||||||||||||||||||||||||||||||||||||||||||||||||||||||
2   15 841  CTTTTTGAGCAAGCATGTCCATCCTTATATCGCCCAGCAATAACACAGGCTGGGGCCTGC  15 900

1   15 901  GCTTCCCAAGCAAGATGTTTGGCGGGGCCAAGAAGCGCTCCGACCAACACCCAGTGCGCG  15 960
            ||||||||||||||||||||||||||||||||||||||||||||||||||||||||||||
2   15 901  GCTTCCCAAGCAAGATGTTTGGCGGGGCCAAGAAGCGCTCCGACCAACACCCAGTGCGCG  15 960

1   15 961  TGCGCGGGCACTACCGCGCGCCCTGGGGCGCGCACAAACGCGGCCGCACTGGGCGCACCA  16 020
            ||||||||||||||||||||||||||||||||||||||||||||||||||||||||||||
2   15 961  TGCGCGGGCACTACCGCGCGCCCTGGGGCGCGCACAAACGCGGCCGCACTGGGCGCACCA  16 020

1   16 021  CCGTCGATGACGCCATCGACGCGGTGGTGGAGGAGGCGCGCAACTACACGCCCACGCCGC  16 080
            ||||||||||||||||||||||||||||||||||||||||||||||||||||||||||||
2   16 021  CCGTCGATGACGCCATCGACGCGGTGGTGGAGGAGGCGCGCAACTACACGCCCACGCCGC  16 080

1   16 081  CACCAGTGTCCACAGTGGACGCGGCCATTCAGACCGTGGTGCGCGGAGCCCGGCGCTATG  16 140
            ||||||||||||||||||||||||||||||||||||||||||||||||||||||||||||
2   16 081  CACCAGTGTCCACAGTGGACGCGGCCATTCAGACCGTGGTGCGCGGAGCCCGGCGCTATG  16 140
```

```
1   16 141  CTAAAATGAAGAGACGGCGGAGGCGCGTAGCACGTCGCCACCGCCGCCGACCCGGCACTG  16 200
            ||||||||||||||||||||||||||||||||||||||||||||||||||||||||||||
2   16 141  CTAAAATGAAGAGACGGCGGAGGCGCGTAGCACGTCGCCACCGCCGCCGACCCGGCACTG  16 200

1   16 201  CCGCCCAACGCGCGGCGGCGGCCCTGCTTAACCGCGCACGTCGCACCGGCCGACGGGCGG  16 260
            ||||||||||||||||||||||||||||||||||||||||||||||||||||||||||||
2   16 201  CCGCCCAACGCGCGGCGGCGGCCCTGCTTAACCGCGCACGTCGCACCGGCCGACGGGCGG  16 260

1   16 261  CCATGCGGGCCGCTCGAAGGCTGGCCGCGGGTATTGTCACTGTGCCCCCCAGGTCCAGGC  16 320
            ||||||||||||||||||||||||||||||||||||||||||||||||||||||||||||
2   16 261  CCATGCGGGCCGCTCGAAGGCTGGCCGCGGGTATTGTCACTGTGCCCCCCAGGTCCAGGC  16 320

1   16 321  GACGAGCGGCCGCCGCAGCAGCCGCGGCCATTAGTGCTATGACTCAGGGTCGCAGGGGCA  16 380
            ||||||||||||||||||||||||||||||||||||||||||||||||||||||||||||
2   16 321  GACGAGCGGCCGCCGCAGCAGCCGCGGCCATTAGTGCTATGACTCAGGGTCGCAGGGGCA  16 380

1   16 381  ACGTGTATTGGGTGCGCGACTCGGTTAGCGGCCTGCGCGTGCCCGTGCGCACCCGCCCCC  16 440
            ||||||||||||||||||||||||||||||||||||||||||||||||||||||||||||
2   16 381  ACGTGTATTGGGTGCGCGACTCGGTTAGCGGCCTGCGCGTGCCCGTGCGCACCCGCCCCC  16 440

1   16 441  CGCGCAACTAGATTGCAAGAAAAAACTACTTAGACTCGTACTGTTGTATGTATCCAGCGG  16 500
            ||||||||||||||||||||||||||||||||||||||||||||||||||||||||||||
2   16 441  CGCGCAACTAGATTGCAAGAAAAAACTACTTAGACTCGTACTGTTGTATGTATCCAGCGG  16 500

1   16 501  CGGCGGCGCGCAACGAAGCTATGTCCAAGCGCAAAATCAAAGAAGAGATGCTCCAGGTCA  16 560
            ||||||||||||||||||||||||||||||||||||||||||||||||||||||||||||
2   16 501  CGGCGGCGCGCAACGAAGCTATGTCCAAGCGCAAAATCAAAGAAGAGATGCTCCAGGTCA  16 560

1   16 561  TCGCGCCGGAGATCTATGGCCCCCCGAAGAAGGAAGAGCAGGATTACAAGCCCCGAAAGC  16 620
            ||||||||||||||||||||||||||||||||||||||||||||||||||||||||||||
2   16 561  TCGCGCCGGAGATCTATGGCCCCCCGAAGAAGGAAGAGCAGGATTACAAGCCCCGAAAGC  16 620

1   16 621  TAAAGCGGGTCAAAAAGAAAAAGAAAGATGATGATGATGAACTTGACGACGAGGTGGAAC  16 680
            ||||||||||||||||||||||||||||||||||||||||||||||||||||||||||||
2   16 621  TAAAGCGGGTCAAAAAGAAAAAGAAAGATGATGATGATGAACTTGACGACGAGGTGGAAC  16 680

1   16 681  TGCTGCACGCTACCGCGCCCAGGCGACGGGTACAGTGGAAAGGTCGACGCGTAAAACGTG  16 740
            ||||||||||||||||||||||||||||||||||||||||||||||||||||||||||||
2   16 681  TGCTGCACGCTACCGCGCCCAGGCGACGGGTACAGTGGAAAGGTCGACGCGTAAAACGTG  16 740
```

```
1  16 741  TTTTGCGACCCGGCACCACCGTAGTCTTTACGCCCGGTGAGCGCTCCACCCGCACCTACA  16 800
           ||||||||||||||||||||||||||||||||||||||||||||||||||||||||||||
2  16 741  TTTTGCGACCCGGCACCACCGTAGTCTTTACGCCCGGTGAGCGCTCCACCCGCACCTACA  16 800

1  16 801  AGCGCGTGTATGATGAGGTGTACGGCGACGAGGACCTGCTTGAGCAGGCCAACGAGCGCC  16 860
           ||||||||||||||||||||||||||||||||||||||||||||||||||||||||||||
2  16 801  AGCGCGTGTATGATGAGGTGTACGGCGACGAGGACCTGCTTGAGCAGGCCAACGAGCGCC  16 860

1  16 861  TCGGGGAGTTTGCCTACGGAAAGCGGCATAAGGACATGCTGGCGTTGCCGCTGGACGAGG  16 920
           ||||||||||||||||||||||||||||||||||||||||||||||||||||||||||||
2  16 861  TCGGGGAGTTTGCCTACGGAAAGCGGCATAAGGACATGCTGGCGTTGCCGCTGGACGAGG  16 920

1  16 921  GCAACCCAACACCTAGCCTAAAGCCCGTAACACTGCAGCAGGTGCTGCCCGCGCTTGCAC  16 980
           ||||||||||||||||||||||||||||||||||||||||||||||||||||||||||||
2  16 921  GCAACCCAACACCTAGCCTAAAGCCCGTAACACTGCAGCAGGTGCTGCCCGCGCTTGCAC  16 980

1  16 981  CGTCCGAAGAAAAGCGCGGCCTAAAGCGCGAGTCTGGTGACTTGGCACCCACCGTGCAGC  17 040
           ||||||||||||||||||||||||||||||||||||||||||||||||||||||||||||
2  16 981  CGTCCGAAGAAAAGCGCGGCCTAAAGCGCGAGTCTGGTGACTTGGCACCCACCGTGCAGC  17 040

1  17 041  TGATGGTACCCAAGCGCCAGCGACTGGAAGATGTCTTGGAAAAAATGACCGTGGAACCTG  17 100
           ||||||||||||||||||||||||||||||||||||||||||||||||||||||||||||
2  17 041  TGATGGTACCCAAGCGCCAGCGACTGGAAGATGTCTTGGAAAAAATGACCGTGGAACCTG  17 100

1  17 101  GGCTGGAGCCCGAGGTCCGCGTGCGGCCAATCAAGCAGGTGGCGCCGGGACTGGGCGTGC  17 160
           ||||||||||||||||||||||||||||||||||||||||||||||||||||||||||||
2  17 101  GGCTGGAGCCCGAGGTCCGCGTGCGGCCAATCAAGCAGGTGGCGCCGGGACTGGGCGTGC  17 160

1  17 161  AGACCGTGGACGTTCAGATACCCACTACCAGTAGCACCAGTATTGCCACCGCCACAGAGG  17 220
           ||||||||||||||||||||||||||||||||||||||||||||||||||||||||||||
2  17 161  AGACCGTGGACGTTCAGATACCCACTACCAGTAGCACCAGTATTGCCACCGCCACAGAGG  17 220

1  17 221  GCATGGAGACACAAACGTCCCCGGTTGCCTCAGCGGTGGCGGATGCCGCGGTGCAGGCGG  17 280
           ||||||||||||||||||||||||||||||||||||||||||||||||||||||||||||
2  17 221  GCATGGAGACACAAACGTCCCCGGTTGCCTCAGCGGTGGCGGATGCCGCGGTGCAGGCGG  17 280

1  17 281  TCGCTGCGGCCGCGTCCAAGACCTCTACGGAGGTGCAAACGGACCCGTGGATGTTTCGCG  17 340
           ||||||||||||||||||||||||||||||||||||||||||||||||||||||||||||
2  17 281  TCGCTGCGGCCGCGTCCAAGACCTCTACGGAGGTGCAAACGGACCCGTGGATGTTTCGCG  17 340
```

```
1   17 341  TTTCAGCCCCCCGGCGCCCGCGCCGTTCGAGGAAGTACGGCGCCGCCAGCGCGCTACTGC  17 400
            ||||||||||||||||||||||||||||||||||||||||||||||||||||||||||||
2   17 341  TTTCAGCCCCCCGGCGCCCGCGCCGTTCGAGGAAGTACGGCGCCGCCAGCGCGCTACTGC  17 400

1   17 401  CCGAATATGCCCTACATCCTTCCATTGCGCCTACCCCCGGCTATCGTGGCTACACCTACC  17 460
            ||||||||||||||||||||||||||||||||||||||||||||||||||||||||||||
2   17 401  CCGAATATGCCCTACATCCTTCCATTGCGCCTACCCCCGGCTATCGTGGCTACACCTACC  17 460

1   17 461  GCCCCAGAAGACGAGCAACTACCCGACGCCGAACCACCACTGGAACCCGCCGCCGCCGTC  17 520
            ||||||||||||||||||||||||||||||||||||||||||||||||||||||||||||
2   17 461  GCCCCAGAAGACGAGCAACTACCCGACGCCGAACCACCACTGGAACCCGCCGCCGCCGTC  17 520

1   17 521  GCCGTCGCCAGCCCGTGCTGGCCCCGATTTCCGTGCGCAGGGTGGCTCGCGAAGGAGGCA  17 580
            ||||||||||||||||||||||||||||||||||||||||||||||||||||||||||||
2   17 521  GCCGTCGCCAGCCCGTGCTGGCCCCGATTTCCGTGCGCAGGGTGGCTCGCGAAGGAGGCA  17 580

1   17 581  GGACCCTGGTGCTGCCAACAGCGCGCTACCACCCCAGCATCGTTTAAAAGCCGGTCTTTG  17 640
            ||||||||||||||||||||||||||||||||||||||||||||||||||||||||||||
2   17 581  GGACCCTGGTGCTGCCAACAGCGCGCTACCACCCCAGCATCGTTTAAAAGCCGGTCTTTG  17 640

1   17 641  TGGTTCTTGCAGATATGGCCCTCACCTGCCGCCTCCGTTTCCCGGTGCCGGGATTCCGAG  17 700
            ||||||||||||||||||||||||||||||||||||||||||||||||||||||||||||
2   17 641  TGGTTCTTGCAGATATGGCCCTCACCTGCCGCCTCCGTTTCCCGGTGCCGGGATTCCGAG  17 700

1   17 701  GAAGAATGCACCGTAGGAGGGGCATGGCCGGCCACGGCCTGACGGGCGGCATGCGTCGTG  17 760
            ||||||||||||||||||||||||||||||||||||||||||||||||||||||||||||
2   17 701  GAAGAATGCACCGTAGGAGGGGCATGGCCGGCCACGGCCTGACGGGCGGCATGCGTCGTG  17 760

1   17 761  CGCACCACCGGCGGCGGCGCGCGTCGCACCGTCGCATGCGCGGCGGTATCCTGCCCCTCC  17 820
            ||||||||||||||||||||||||||||||||||||||||||||||||||||||||||||
2   17 761  CGCACCACCGGCGGCGGCGCGCGTCGCACCGTCGCATGCGCGGCGGTATCCTGCCCCTCC  17 820

1   17 821  TTATTCCACTGATCGCCGCGGCGATTGGCGCCGTGCCCGGAATTGCATCCGTGGCCTTGC  17 880
            ||||||||||||||||||||||||||||||||||||||||||||||||||||||||||||
2   17 821  TTATTCCACTGATCGCCGCGGCGATTGGCGCCGTGCCCGGAATTGCATCCGTGGCCTTGC  17 880

1   17 881  AGGCGCAGAGACACTGATTAAAAACAAGTTGCATGTGGAAAAATCAAAATAAAAAGTCTG  17 940
            ||||||||||||||||||||||||||||||||||||||||||||||||||||||||||||
2   17 881  AGGCGCAGAGACACTGATTAAAAACAAGTTGCATGTGGAAAAATCAAAATAAAAAGTCTG  17 940
```

```
1   17 941  GACTCTCACGCTCGCTTGGTCCTGTAACTATTTTGTAGAATGGAAGACATCAACTTTGCG  18 000
            ||||||||||||||||||||||||||||||||||||||||||||||||||||||||||||
2   17 941  GACTCTCACGCTCGCTTGGTCCTGTAACTATTTTGTAGAATGGAAGACATCAACTTTGCG  18 000

1   18 001  TCTCTGGCCCCGCGACACGGCTCGCGCCCGTTCATGGGAAACTGGCAAGATATCGGCACC  18 060
            ||||||||||||||||||||||||||||||||||||||||||||||||||||||||||||
2   18 001  TCTCTGGCCCCGCGACACGGCTCGCGCCCGTTCATGGGAAACTGGCAAGATATCGGCACC  18 060

1   18 061  AGCAATATGAGCGGTGGCGCCTTCAGCTGGGGCTCGCTGTGGAGCGGCATTAAAAATTTC  18 120
            ||||||||||||||||||||||||||||||||||||||||||||||||||||||||||||
2   18 061  AGCAATATGAGCGGTGGCGCCTTCAGCTGGGGCTCGCTGTGGAGCGGCATTAAAAATTTC  18 120

1   18 121  GGTTCCACCGTTAAGAACTATGGCAGCAAGGCCTGGAACAGCAGCACAGGCCAGATGCTG  18 180
            ||||||||||||||||||||||||||||||||||||||||||||||||||||||||||||
2   18 121  GGTTCCACCGTTAAGAACTATGGCAGCAAGGCCTGGAACAGCAGCACAGGCCAGATGCTG  18 180

1   18 181  AGGGATAAGTTGAAAGAGCAAAATTTCCAACAAAAGGTGGTAGATGGCCTGGCCTCTGGC  18 240
            ||||||||||||||||||||||||||||||||||||||||||||||||||||||||||||
2   18 181  AGGGATAAGTTGAAAGAGCAAAATTTCCAACAAAAGGTGGTAGATGGCCTGGCCTCTGGC  18 240

1   18 241  ATTAGCGGGGTGGTGGACCTGGCCAACCAGGCAGTGCAAAATAAGATTAACAGTAAGCTT  18 300
            ||||||||||||||||||||||||||||||||||||||||||||||||||||||||||||
2   18 241  ATTAGCGGGGTGGTGGACCTGGCCAACCAGGCAGTGCAAAATAAGATTAACAGTAAGCTT  18 300

1   18 301  GATCCCCGCCCTCCCGTAGAGGAGCCTCCACCGGCCGTGGAGACAGTGTCTCCAGAGGGG  18 360
            ||||||||||||||||||||||||||||||||||||||||||||||||||||||||||||
2   18 301  GATCCCCGCCCTCCCGTAGAGGAGCCTCCACCGGCCGTGGAGACAGTGTCTCCAGAGGGG  18 360

1   18 361  CGTGGCGAAAAGCGTCCGCGCCCCGACAGGGAAGAAACTCTGGTGACGCAAATAGACGAG  18 420
            ||||||||||||||||||||||||||||||||||||||||||||||||||||||||||||
2   18 361  CGTGGCGAAAAGCGTCCGCGCCCCGACAGGGAAGAAACTCTGGTGACGCAAATAGACGAG  18 420

1   18 421  CCTCCCTCGTACGAGGAGGCACTAAAGCAAGGCCTGCCCACCACCCGTCCCATCGCGCCC  18 480
            ||||||||||||||||||||||||||||||||||||||||||||||||||||||||||||
2   18 421  CCTCCCTCGTACGAGGAGGCACTAAAGCAAGGCCTGCCCACCACCCGTCCCATCGCGCCC  18 480

1   18 481  ATGGCTACCGGAGTGCTGGGCCAGCACACACCCGTAACGCTGGACCTGCCTCCCCCCGCC  18 540
            ||||||||||||||||||||||||||||||||||||||||||||||||||||||||||||
2   18 481  ATGGCTACCGGAGTGCTGGGCCAGCACACACCCGTAACGCTGGACCTGCCTCCCCCCGCC  18 540
```

```
1   18 541  GACACCCAGCAGAAACCTGTGCTGCCAGGCCCGACCGCCGTTGTTGTAACCCGTCCTAGC  18 600
            ||||||||||||||||||||||||||||||||||||||||||||||||||||||||||||
2   18 541  GACACCCAGCAGAAACCTGTGCTGCCAGGCCCGACCGCCGTTGTTGTAACCCGTCCTAGC  18 600

1   18 601  CGCGCGTCCCTGCGCCGCGCCGCCAGCGGTCCGCGATCGTTGCGGCCCGTAGCCAGTGGC  18 660
            ||||||||||||||||||||||||||||||||||||||||||||||||||||||||||||
2   18 601  CGCGCGTCCCTGCGCCGCGCCGCCAGCGGTCCGCGATCGTTGCGGCCCGTAGCCAGTGGC  18 660

1   18 661  AACTGGCAAAGCACACTGAACAGCATCGTGGGTCTGGGGGTGCAATCCCTGAAGCGCCGA  18 720
            ||||||||||||||||||||||||||||||||||||||||||||||||||||||||||||
2   18 661  AACTGGCAAAGCACACTGAACAGCATCGTGGGTCTGGGGGTGCAATCCCTGAAGCGCCGA  18 720

1   18 721  CGATGCTTCTGATAGCTAACGTGTCGTATGTGTGTCATGTATGCGTCCATGTCGCCGCCA  18 780
            ||||||||||||||||||||||||||||||||||||||||||||||||||||||||||||
2   18 721  CGATGCTTCTGATAGCTAACGTGTCGTATGTGTGTCATGTATGCGTCCATGTCGCCGCCA  18 780

1   18 781  GAGGAGCTGCTGAGCCGCCGCGCGCCCGCTTTCCAAGATGGCTACCCCTTCGATGATGCC  18 840
            ||||||||||||||||||||||||||||||||||||||||||||||||||||||||||||
2   18 781  GAGGAGCTGCTGAGCCGCCGCGCGCCCGCTTTCCAAGATGGCTACCCCTTCGATGATGCC  18 840

1   18 841  GCAGTGGTCTTACATGCACATCTCGGGCCAGGACGCCTCGGAGTACCTGAGCCCCGGGCT  18 900
            ||||||||||||||||||||||||||||||||||||||||||||||||||||||||||||
2   18 841  GCAGTGGTCTTACATGCACATCTCGGGCCAGGACGCCTCGGAGTACCTGAGCCCCGGGCT  18 900

1   18 901  GGTGCAGTTTGCCCGCGCCACCGAGACGTACTTCAGCCTGAATAACAAGTTTAGAAACCC  18 960
            ||||||||||||||||||||||||||||||||||||||||||||||||||||||||||||
2   18 901  GGTGCAGTTTGCCCGCGCCACCGAGACGTACTTCAGCCTGAATAACAAGTTTAGAAACCC  18 960

1   18 961  CACGGTGGCGCCTACGCACGACGTGACCACAGACCGGTCCCAGCGTTTGACGCTGCGGTT  19 020
            ||||||||||||||||||||||||||||||||||||||||||||||||||||||||||||
2   18 961  CACGGTGGCGCCTACGCACGACGTGACCACAGACCGGTCCCAGCGTTTGACGCTGCGGTT  19 020

1   19 021  CATCCCTGTGGACCGTGAGGATACTGCGTACTCGTACAAGGCGCGGTTCACCCTAGCTGT  19 080
            ||||||||||||||||||||||||||||||||||||||||||||||||||||||||||||
2   19 021  CATCCCTGTGGACCGTGAGGATACTGCGTACTCGTACAAGGCGCGGTTCACCCTAGCTGT  19 080

1   19 081  GGGTGATAACCGTGTGCTGGACATGGCTTCCACGTACTTTGACATCCGCGGCGTGCTGGA  19 140
            ||||||||||||||||||||||||||||||||||||||||||||||||||||||||||||
2   19 081  GGGTGATAACCGTGTGCTGGACATGGCTTCCACGTACTTTGACATCCGCGGCGTGCTGGA  19 140
```

```
1   19 141  CAGGGGCCCTACTTTTAAGCCCTACTCTGGCACTGCCTACAACGCCCTGGCTCCCAAGGG  19 200
            ||||||||||||||||||||||||||||||||||||||||||||||||||||||||||||
2   19 141  CAGGGGCCCTACTTTTAAGCCCTACTCTGGCACTGCCTACAACGCCCTGGCTCCCAAGGG  19 200

1   19 201  TGCCCCAAATCCTTGCGAATGGGATGAAGCTGCTACTGCTCTTGAAATAAACCTAGAAGA  19 260
            ||||||||||||||||||||||||||||||||||||||||||||||||||||||||||||
2   19 201  TGCCCCAAATCCTTGCGAATGGGATGAAGCTGCTACTGCTCTTGAAATAAACCTAGAAGA  19 260

1   19 261  AGAGGACGATGACAACGAAGACGAAGTAGACGAGCAAGCTGAGCAGCAAAAAACTCACGT  19 320
            ||||||||||||||||||||||||||||||||||||||||||||||||||||||||||||
2   19 261  AGAGGACGATGACAACGAAGACGAAGTAGACGAGCAAGCTGAGCAGCAAAAAACTCACGT  19 320

1   19 321  ATTTGGGCAGGCGCCTTATTCTGGTATAAATATTACAAAGGAGGGTATTCAAATAGGTGT  19 380
            ||||||||||||||||||||||||||||||||||||||||||||||||||||||||||||
2   19 321  ATTTGGGCAGGCGCCTTATTCTGGTATAAATATTACAAAGGAGGGTATTCAAATAGGTGT  19 380

1   19 381  CGAAGGTCAAACACCTAAATATGCCGATAAAACATTTCAACCTGAACCTCAAATAGGAGA  19 440
            ||||||||||||||||||||||||||||||||||||||||||||||||||||||||||||
2   19 381  CGAAGGTCAAACACCTAAATATGCCGATAAAACATTTCAACCTGAACCTCAAATAGGAGA  19 440

1   19 441  ATCTCAGTGGTACGAAACAGAAATTAATCATGCAGCTGGGAGAGTCCTAAAAAAGACTAC  19 500
            ||||||||||||||||||||||||||||||||||||||||||||||||||||||||||||
2   19 441  ATCTCAGTGGTACGAAACAGAAATTAATCATGCAGCTGGGAGAGTCCTAAAAAAGACTAC  19 500

1   19 501  CCCAATGAAACCATGTTACGGTTCATATGCAAAACCCACAAATGAAAATGGAGGGCAAGG  19 560
            ||||||||||||||||||||||||||||||||||||||||||||||||||||||||||||
2   19 501  CCCAATGAAACCATGTTACGGTTCATATGCAAAACCCACAAATGAAAATGGAGGGCAAGG  19 560

1   19 561  CATTCTTGTAAAGCAACAAAATGGAAAGCTAGAAAGTCAAGTGGAAATGCAATTTTTCTC  19 620
            ||||||||||||||||||||||||||||||||||||||||||||||||||||||||||||
2   19 561  CATTCTTGTAAAGCAACAAAATGGAAAGCTAGAAAGTCAAGTGGAAATGCAATTTTTCTC  19 620

1   19 621  AACTACTGAGGCAGCCGCAGGCAATGGTGATAACTTGACTCCTAAAGTGGTATTGTACAG  19 680
            ||||||||||||||||||||||||||||||||||||||||||||||||||||||||||||
2   19 621  AACTACTGAGGCAGCCGCAGGCAATGGTGATAACTTGACTCCTAAAGTGGTATTGTACAG  19 680

1   19 681  TGAAGATGTAGATATAGAAACCCCAGACACTCATATTTCTTACATGCCCACTATTAAGGA  19 740
            ||||||||||||||||||||||||||||||||||||||||||||||||||||||||||||
2   19 681  TGAAGATGTAGATATAGAAACCCCAGACACTCATATTTCTTACATGCCCACTATTAAGGA  19 740
```

```
1   19 741  AGGTAACTCACGAGAACTAATGGGCCAACAATCTATGCCCAACAGGCCTAATTACATTGC  19 800
            ||||||||||||||||||||||||||||||||||||||||||||||||||||||||||||
2   19 741  AGGTAACTCACGAGAACTAATGGGCCAACAATCTATGCCCAACAGGCCTAATTACATTGC  19 800

1   19 801  TTTTAGGGACAATTTTATTGGTCTAATGTATTACAACAGCACGGGTAATATGGGTGTTCT  19 860
            ||||||||||||||||||||||||||||||||||||||||||||||||||||||||||||
2   19 801  TTTTAGGGACAATTTTATTGGTCTAATGTATTACAACAGCACGGGTAATATGGGTGTTCT  19 860

1   19 861  GGCGGGCCAAGCATCGCAGTTGAATGCTGTTGTAGATTTGCAAGACAGAAACACAGAGCT  19 920
            ||||||||||||||||||||||||||||||||||||||||||||||||||||||||||||
2   19 861  GGCGGGCCAAGCATCGCAGTTGAATGCTGTTGTAGATTTGCAAGACAGAAACACAGAGCT  19 920

1   19 921  TTCATACCAGCTTTTGCTTGATTCCATTGGTGATAGAACCAGGTACTTTTCTATGTGGAA  19 980
            ||||||||||||||||||||||||||||||||||||||||||||||||||||||||||||
2   19 921  TTCATACCAGCTTTTGCTTGATTCCATTGGTGATAGAACCAGGTACTTTTCTATGTGGAA  19 980

1   19 981  TCAGGCTGTTGACAGCTATGATCCAGATGTTAGAATTATTGAAAATCATGGAACTGAAGA  20 040
            ||||||||||||||||||||||||||||||||||||||||||||||||||||||||||||
2   19 981  TCAGGCTGTTGACAGCTATGATCCAGATGTTAGAATTATTGAAAATCATGGAACTGAAGA  20 040

1   20 041  TGAACTTCCAAATTACTGCTTTCCACTGGGAGGTGTGATTAATACAGAGACTCTTACCAA  20 100
            ||||||||||||||||||||||||||||||||||||||||||||||||||||||||||||
2   20 041  TGAACTTCCAAATTACTGCTTTCCACTGGGAGGTGTGATTAATACAGAGACTCTTACCAA  20 100

1   20 101  GGTAAAACCTAAAACAGGTCAGGAAAATGGATGGGAAAAAGATGCTACAGAATTTTCAGA  20 160
            ||||||||||||||||||||||||||||||||||||||||||||||||||||||||||||
2   20 101  GGTAAAACCTAAAACAGGTCAGGAAAATGGATGGGAAAAAGATGCTACAGAATTTTCAGA  20 160

1   20 161  TAAAAATGAAATAAGAGTTGGAAATAATTTTGCCATGGAAATCAATCTAAATGCCAACCT  20 220
            ||||||||||||||||||||||||||||||||||||||||||||||||||||||||||||
2   20 161  TAAAAATGAAATAAGAGTTGGAAATAATTTTGCCATGGAAATCAATCTAAATGCCAACCT  20 220

1   20 221  GTGGAGAAATTTCCTGTACTCCAACATAGCGCTGTATTTGCCCGACAAGCTAAAGTACAG  20 280
            ||||||||||||||||||||||||||||||||||||||||||||||||||||||||||||
2   20 221  GTGGAGAAATTTCCTGTACTCCAACATAGCGCTGTATTTGCCCGACAAGCTAAAGTACAG  20 280

1   20 281  TCCTTCCAACGTAAAAATTTCTGATAACCCAAACACCTACGACTACATGAACAAGCGAGT  20 340
            ||||||||||||||||||||||||||||||||||||||||||||||||||||||||||||
2   20 281  TCCTTCCAACGTAAAAATTTCTGATAACCCAAACACCTACGACTACATGAACAAGCGAGT  20 340
```

```
1    20 341  GGTGGCTCCCGGGCTAGTGGACTGCTACATTAACCTTGGAGCACGCTGGTCCCTTGACTA  20 400
             ||||||||||||||||||||||||||||||||||||||||||||||||||||||||||||
2    20 341  GGTGGCTCCCGGGCTAGTGGACTGCTACATTAACCTTGGAGCACGCTGGTCCCTTGACTA  20 400

1    20 401  TATGGACAACGTCAACCCATTTAACCACCACCGCAATGCTGGCCTGCGCTACCGCTCAAT  20 460
             ||||||||||||||||||||||||||||||||||||||||||||||||||||||||||||
2    20 401  TATGGACAACGTCAACCCATTTAACCACCACCGCAATGCTGGCCTGCGCTACCGCTCAAT  20 460

1    20 461  GTTGCTGGGCAATGGTCGCTATGTGCCCTTCCACATCCAGGTGCCTCAGAAGTTCTTTGC  20 520
             ||||||||||||||||||||||||||||||||||||||||||||||||||||||||||||
2    20 461  GTTGCTGGGCAATGGTCGCTATGTGCCCTTCCACATCCAGGTGCCTCAGAAGTTCTTTGC  20 520

1    20 521  CATTAAAAACCTCCTTCTCCTGCCGGGCTCATACACCTACGAGTGGAACTTCAGGAAGGA  20 580
             ||||||||||||||||||||||||||||||||||||||||||||||||||||||||||||
2    20 521  CATTAAAAACCTCCTTCTCCTGCCGGGCTCATACACCTACGAGTGGAACTTCAGGAAGGA  20 580

1    20 581  TGTTAACATGGTTCTGCAGAGCTCCCTAGGAAATGACCTAAGGGTTGACGGAGCCAGCAT  20 640
             ||||||||||||||||||||||||||||||||||||||||||||||||||||||||||||
2    20 581  TGTTAACATGGTTCTGCAGAGCTCCCTAGGAAATGACCTAAGGGTTGACGGAGCCAGCAT  20 640

1    20 641  TAAGTTTGATAGCATTTGCCTTTACGCCACCTTCTTCCCCATGGCCCACAACACCGCCTC  20 700
             ||||||||||||||||||||||||||||||||||||||||||||||||||||||||||||
2    20 641  TAAGTTTGATAGCATTTGCCTTTACGCCACCTTCTTCCCCATGGCCCACAACACCGCCTC  20 700

1    20 701  CACGCTTGAGGCCATGCTTAGAAACGACACCAACGACCAGTCCTTTAACGACTATCTCTC  20 760
             ||||||||||||||||||||||||||||||||||||||||||||||||||||||||||||
2    20 701  CACGCTTGAGGCCATGCTTAGAAACGACACCAACGACCAGTCCTTTAACGACTATCTCTC  20 760

1    20 761  CGCCGCCAACATGCTCTACCCTATACCCGCCAACGCTACCAACGTGCCCATATCCATCCC  20 820
             ||||||||||||||||||||||||||||||||||||||||||||||||||||||||||||
2    20 761  CGCCGCCAACATGCTCTACCCTATACCCGCCAACGCTACCAACGTGCCCATATCCATCCC  20 820

1    20 821  CTCCCGCAACTGGGCGGCTTTCCGCGGCTGGGCCTTCACGCGCCTTAAGACTAAGGAAAC  20 880
             ||||||||||||||||||||||||||||||||||||||||||||||||||||||||||||
2    20 821  CTCCCGCAACTGGGCGGCTTTCCGCGGCTGGGCCTTCACGCGCCTTAAGACTAAGGAAAC  20 880

1    20 881  CCCATCACTGGGCTCGGGCTACGACCCTTATTACACCTACTCTGGCTCTATACCCTACCT  20 940
             ||||||||||||||||||||||||||||||||||||||||||||||||||||||||||||
2    20 881  CCCATCACTGGGCTCGGGCTACGACCCTTATTACACCTACTCTGGCTCTATACCCTACCT  20 940
```

```
1    20 941  AGATGGAACCTTTTACCTCAACCACACCTTTAAGAAGGTGGCCATTACCTTTGACTCTTC  21 000
             ||||||||||||||||||||||||||||||||||||||||||||||||||||||||||||
2    20 941  AGATGGAACCTTTTACCTCAACCACACCTTTAAGAAGGTGGCCATTACCTTTGACTCTTC  21 000

1    21 001  TGTCAGCTGGCCTGGCAATGACCGCCTGCTTACCCCCAACGAGTTTGAAATTAAGCGCTC  21 060
             ||||||||||||||||||||||||||||||||||||||||||||||||||||||||||||
2    21 001  TGTCAGCTGGCCTGGCAATGACCGCCTGCTTACCCCCAACGAGTTTGAAATTAAGCGCTC  21 060

1    21 061  AGTTGACGGGGAGGGTTACAACGTTGCCCAGTGTAACATGACCAAAGACTGGTTCCTGGT  21 120
             ||||||||||||||||||||||||||||||||||||||||||||||||||||||||||||
2    21 061  AGTTGACGGGGAGGGTTACAACGTTGCCCAGTGTAACATGACCAAAGACTGGTTCCTGGT  21 120

1    21 121  ACAAATGCTAGCTAACTATAACATTGGCTACCAGGGCTTCTATATCCCAGAGAGCTACAA  21 180
             ||||||||||||||||||||||||||||||||||||||||||||||||||||||||||||
2    21 121  ACAAATGCTAGCTAACTATAACATTGGCTACCAGGGCTTCTATATCCCAGAGAGCTACAA  21 180

1    21 181  GGACCGCATGTACTCCTTCTTTAGAAACTTCCAGCCCATGAGCCGTCAGGTGGTGGATGA  21 240
             ||||||||||||||||||||||||||||||||||||||||||||||||||||||||||||
2    21 181  GGACCGCATGTACTCCTTCTTTAGAAACTTCCAGCCCATGAGCCGTCAGGTGGTGGATGA  21 240

1    21 241  TACTAAATACAAGGACTACCAACAGGTGGGCATCCTACACCAACACAACAACTCTGGATT  21 300
             ||||||||||||||||||||||||||||||||||||||||||||||||||||||||||||
2    21 241  TACTAAATACAAGGACTACCAACAGGTGGGCATCCTACACCAACACAACAACTCTGGATT  21 300

1    21 301  TGTTGGCTACCTTGCCCCCACCATGCGCGAAGGACAGGCCTACCCTGCTAACTTCCCCTA  21 360
             ||||||||||||||||||||||||||||||||||||||||||||||||||||||||||||
2    21 301  TGTTGGCTACCTTGCCCCCACCATGCGCGAAGGACAGGCCTACCCTGCTAACTTCCCCTA  21 360

1    21 361  TCCGCTTATAGGCAAGACCGCAGTTGACAGCATTACCCAGAAAAAGTTTCTTTGCGATCG  21 420
             ||||||||||||||||||||||||||||||||||||||||||||||||||||||||||||
2    21 361  TCCGCTTATAGGCAAGACCGCAGTTGACAGCATTACCCAGAAAAAGTTTCTTTGCGATCG  21 420

1    21 421  CACCCTTTGGCGCATCCCATTCTCCAGTAACTTTATGTCCATGGGCGCACTCACAGACCT  21 480
             ||||||||||||||||||||||||||||||||||||||||||||||||||||||||||||
2    21 421  CACCCTTTGGCGCATCCCATTCTCCAGTAACTTTATGTCCATGGGCGCACTCACAGACCT  21 480

1    21 481  GGGCCAAAACCTTCTCTACGCCAACTCCGCCCACGCGCTAGACATGACTTTTGAGGTGGA  21 540
             ||||||||||||||||||||||||||||||||||||||||||||||||||||||||||||
2    21 481  GGGCCAAAACCTTCTCTACGCCAACTCCGCCCACGCGCTAGACATGACTTTTGAGGTGGA  21 540
```

```
1    21 541  TCCCATGGACGAGCCCACCCTTCTTTATGTTTTGTTTGAAGTCTTTGACGTGGTCCGTGT  21 600
             ||||||||||||||||||||||||||||||||||||||||||||||||||||||||||||
2    21 541  TCCCATGGACGAGCCCACCCTTCTTTATGTTTTGTTTGAAGTCTTTGACGTGGTCCGTGT  21 600

1    21 601  GCACCAGCCGCACCGCGGCGTCATCGAAACCGTGTACCTGCGCACGCCCTTCTCGGCCGG  21 660
             ||||||||||||||||||||||||||||||||||||||||||||||||||||||||||||
2    21 601  GCACCAGCCGCACCGCGGCGTCATCGAAACCGTGTACCTGCGCACGCCCTTCTCGGCCGG  21 660

1    21 661  CAACGCCACAACATAAAGAAGCAAGCAACATCAACAACAGCTGCCGCCATGGGCTCCAGT  21 720
             ||||||||||||||||||||||||||||||||||||||||||||||||||||||||||||
2    21 661  CAACGCCACAACATAAAGAAGCAAGCAACATCAACAACAGCTGCCGCCATGGGCTCCAGT  21 720

1    21 721  GAGCAGGAACTGAAAGCCATTGTCAAAGATCTTGGTTGTGGGCCATATTTTTTGGGCACC  21 780
             ||||||||||||||||||||||||||||||||||||||||||||||||||||||||||||
2    21 721  GAGCAGGAACTGAAAGCCATTGTCAAAGATCTTGGTTGTGGGCCATATTTTTTGGGCACC  21 780

1    21 781  TATGACAAGCGCTTTCCAGGCTTTGTTTCTCCACACAAGCTCGCCTGCGCCATAGTCAAT  21 840
             ||||||||||||||||||||||||||||||||||||||||||||||||||||||||||||
2    21 781  TATGACAAGCGCTTTCCAGGCTTTGTTTCTCCACACAAGCTCGCCTGCGCCATAGTCAAT  21 840

1    21 841  ACGGCCGGTCGCGAGACTGGGGGCGTACACTGGATGGCCTTTGCCTGGAACCCGCACTCA  21 900
             ||||||||||||||||||||||||||||||||||||||||||||||||||||||||||||
2    21 841  ACGGCCGGTCGCGAGACTGGGGGCGTACACTGGATGGCCTTTGCCTGGAACCCGCACTCA  21 900

1    21 901  AAAACATGCTACCTCTTTGAGCCCTTTGGCTTTTCTGACCAGCGACTCAAGCAGGTTTAC  21 960
             ||||||||||||||||||||||||||||||||||||||||||||||||||||||||||||
2    21 901  AAAACATGCTACCTCTTTGAGCCCTTTGGCTTTTCTGACCAGCGACTCAAGCAGGTTTAC  21 960

1    21 961  CAGTTTGAGTACGAGTCACTCCTGCGCCGTAGCGCCATTGCTTCTTCCCCCGACCGCTGT  22 020
             ||||||||||||||||||||||||||||||||||||||||||||||||||||||||||||
2    21 961  CAGTTTGAGTACGAGTCACTCCTGCGCCGTAGCGCCATTGCTTCTTCCCCCGACCGCTGT  22 020

1    22 021  ATAACGCTGGAAAAGTCCACCCAAAGCGTACAGGGGCCCAACTCGGCCGCCTGTGGACTA  22 080
             ||||||||||||||||||||||||||||||||||||||||||||||||||||||||||||
2    22 021  ATAACGCTGGAAAAGTCCACCCAAAGCGTACAGGGGCCCAACTCGGCCGCCTGTGGACTA  22 080

1    22 081  TTCTGCTGCATGTTTCTCCACGCCTTTGCCAACTGGCCCCAAACTCCCATGGATCACAAC  22 140
             ||||||||||||||||||||||||||||||||||||||||||||||||||||||||||||
2    22 081  TTCTGCTGCATGTTTCTCCACGCCTTTGCCAACTGGCCCCAAACTCCCATGGATCACAAC  22 140
```

```
1   22 141  CCCACCATGAACCTTATTACCGGGGTACCCAACTCCATGCTCAACAGTCCCCAGGTACAG  22 200
            ||||||||||||||||||||||||||||||||||||||||||||||||||||||||||||
2   22 141  CCCACCATGAACCTTATTACCGGGGTACCCAACTCCATGCTCAACAGTCCCCAGGTACAG  22 200

1   22 201  CCCACCCTGCGTCGCAACCAGGAACAGCTCTACAGCTTCCTGGAGCGCCACTCGCCCTAC  22 260
            ||||||||||||||||||||||||||||||||||||||||||||||||||||||||||||
2   22 201  CCCACCCTGCGTCGCAACCAGGAACAGCTCTACAGCTTCCTGGAGCGCCACTCGCCCTAC  22 260

1   22 261  TTCCGCAGCCACAGTGCGCAGATTAGGAGCGCCACTTCTTTTTGTCACTTGAAAAACATG  22 320
            ||||||||||||||||||||||||||||||||||||||||||||||||||||||||||||
2   22 261  TTCCGCAGCCACAGTGCGCAGATTAGGAGCGCCACTTCTTTTTGTCACTTGAAAAACATG  22 320

1   22 321  TAAAAATAATGTACTAGAGACACTTTCAATAAAGGCAAATGCTTTTATTTGTACACTCTC  22 380
            ||||||||||||||||||||||||||||||||||||||||||||||||||||||||||||
2   22 321  TAAAAATAATGTACTAGAGACACTTTCAATAAAGGCAAATGCTTTTATTTGTACACTCTC  22 380

1   22 381  GGGTGATTATTTACCCCCACCCTTGCCGTCTGCGCCGTTTAAAAATCAAAGGGGTTCTGC  22 440
            ||||||||||||||||||||||||||||||||||||||||||||||||||||||||||||
2   22 381  GGGTGATTATTTACCCCCACCCTTGCCGTCTGCGCCGTTTAAAAATCAAAGGGGTTCTGC  22 440

1   22 441  CGCGCATCGCTATGCGCCACTGGCAGGGACACGTTGCGATACTGGTGTTTAGTGCTCCAC  22 500
            ||||||||||||||||||||||||||||||||||||||||||||||||||||||||||||
2   22 441  CGCGCATCGCTATGCGCCACTGGCAGGGACACGTTGCGATACTGGTGTTTAGTGCTCCAC  22 500

1   22 501  TTAAACTCAGGCACAACCATCCGCGGCAGCTCGGTGAAGTTTTCACTCCACAGGCTGCGC  22 560
            ||||||||||||||||||||||||||||||||||||||||||||||||||||||||||||
2   22 501  TTAAACTCAGGCACAACCATCCGCGGCAGCTCGGTGAAGTTTTCACTCCACAGGCTGCGC  22 560

1   22 561  ACCATCACCAACGCGTTTAGCAGGTCGGGCGCCGATATCTTGAAGTCGCAGTTGGGGCCT  22 620
            ||||||||||||||||||||||||||||||||||||||||||||||||||||||||||||
2   22 561  ACCATCACCAACGCGTTTAGCAGGTCGGGCGCCGATATCTTGAAGTCGCAGTTGGGGCCT  22 620

1   22 621  CCGCCCTGCGCGCGCGAGTTGCGATACACAGGGTTGCAGCACTGGAACACTATCAGCGCC  22 680
            ||||||||||||||||||||||||||||||||||||||||||||||||||||||||||||
2   22 621  CCGCCCTGCGCGCGCGAGTTGCGATACACAGGGTTGCAGCACTGGAACACTATCAGCGCC  22 680

1   22 681  GGGTGGTGCACGCTGGCCAGCACGCTCTTGTCGGAGATCAGATCCGCGTCCAGGTCCTCC  22 740
            ||||||||||||||||||||||||||||||||||||||||||||||||||||||||||||
2   22 681  GGGTGGTGCACGCTGGCCAGCACGCTCTTGTCGGAGATCAGATCCGCGTCCAGGTCCTCC  22 740
```

```
1   22 741   GCGTTGCTCAGGGCGAACGGAGTCAACTTTGGTAGCTGCCTTCCCAAAAAGGGCGCGTGC   22 800
             ||||||||||||||||||||||||||||||||||||||||||||||||||||||||||||
2   22 741   GCGTTGCTCAGGGCGAACGGAGTCAACTTTGGTAGCTGCCTTCCCAAAAAGGGCGCGTGC   22 800

1   22 801   CCAGGCTTTGAGTTGCACTCGCACCGTAGTGGCATCAAAAGGTGACCGTGCCCGGTCTGG   22 860
             ||||||||||||||||||||||||||||||||||||||||||||||||||||||||||||
2   22 801   CCAGGCTTTGAGTTGCACTCGCACCGTAGTGGCATCAAAAGGTGACCGTGCCCGGTCTGG   22 860

1   22 861   GCGTTAGGATACAGCGCCTGCATAAAAGCCTTGATCTGCTTAAAAGCCACCTGAGCCTTT   22 920
             ||||||||||||||||||||||||||||||||||||||||||||||||||||||||||||
2   22 861   GCGTTAGGATACAGCGCCTGCATAAAAGCCTTGATCTGCTTAAAAGCCACCTGAGCCTTT   22 920

1   22 921   GCGCCTTCAGAGAAGAACATGCCGCAAGACTTGCCGGAAAACTGATTGGCCGGACAGGCC   22 980
             ||||||||||||||||||||||||||||||||||||||||||||||||||||||||||||
2   22 921   GCGCCTTCAGAGAAGAACATGCCGCAAGACTTGCCGGAAAACTGATTGGCCGGACAGGCC   22 980

1   22 981   GCGTCGTGCACGCAGCACCTTGCGTCGGTGTTGGAGATCTGCACCACATTTCGGCCCCAC   23 040
             ||||||||||||||||||||||||||||||||||||||||||||||||||||||||||||
2   22 981   GCGTCGTGCACGCAGCACCTTGCGTCGGTGTTGGAGATCTGCACCACATTTCGGCCCCAC   23 040

1   23 041   CGGTTCTTCACGATCTTGGCCTTGCTAGACTGCTCCTTCAGCGCGCGCTGCCCGTTTTCG   23 100
             ||||||||||||||||||||||||||||||||||||||||||||||||||||||||||||
2   23 041   CGGTTCTTCACGATCTTGGCCTTGCTAGACTGCTCCTTCAGCGCGCGCTGCCCGTTTTCG   23 100

1   23 101   CTCGTCACATCCATTTCAATCACGTGCTCCTTATTTATCATAATGCTTCCGTGTAGACAC   23 160
             ||||||||||||||||||||||||||||||||||||||||||||||||||||||||||||
2   23 101   CTCGTCACATCCATTTCAATCACGTGCTCCTTATTTATCATAATGCTTCCGTGTAGACAC   23 160

1   23 161   TTAAGCTCGCCTTCGATCTCAGCGCAGCGGTGCAGCCACAACGCGCAGCCCGTGGGCTCG   23 220
             ||||||||||||||||||||||||||||||||||||||||||||||||||||||||||||
2   23 161   TTAAGCTCGCCTTCGATCTCAGCGCAGCGGTGCAGCCACAACGCGCAGCCCGTGGGCTCG   23 220

1   23 221   TGATGCTTGTAGGTCACCTCTGCAAACGACTGCAGGTACGCCTGCAGGAATCGCCCCATC   23 280
             ||||||||||||||||||||||||||||||||||||||||||||||||||||||||||||
2   23 221   TGATGCTTGTAGGTCACCTCTGCAAACGACTGCAGGTACGCCTGCAGGAATCGCCCCATC   23 280

1   23 281   ATCGTCACAAAGGTCTTGTTGCTGGTGAAGGTCAGCTGCAACCCGCGGTGCTCCTCGTTC   23 340
             ||||||||||||||||||||||||||||||||||||||||||||||||||||||||||||
2   23 281   ATCGTCACAAAGGTCTTGTTGCTGGTGAAGGTCAGCTGCAACCCGCGGTGCTCCTCGTTC   23 340
```

```
1   23 341  AGCCAGGTCTTGCATACGGCCGCCAGAGCTTCCACTTGGTCAGGCAGTAGTTTGAAGTTC  23 400
            ||||||||||||||||||||||||||||||||||||||||||||||||||||||||||||
2   23 341  AGCCAGGTCTTGCATACGGCCGCCAGAGCTTCCACTTGGTCAGGCAGTAGTTTGAAGTTC  23 400

1   23 401  GCCTTTAGATCGTTATCCACGTGGTACTTGTCCATCAGCGCGCGCGCAGCCTCCATGCCC  23 460
            ||||||||||||||||||||||||||||||||||||||||||||||||||||||||||||
2   23 401  GCCTTTAGATCGTTATCCACGTGGTACTTGTCCATCAGCGCGCGCGCAGCCTCCATGCCC  23 460

1   23 461  TTCTCCCACGCAGACACGATCGGCACACTCAGCGGGTTCATCACCGTAATTTCACTTTCC  23 520
            ||||||||||||||||||||||||||||||||||||||||||||||||||||||||||||
2   23 461  TTCTCCCACGCAGACACGATCGGCACACTCAGCGGGTTCATCACCGTAATTTCACTTTCC  23 520

1   23 521  GCTTCGCTGGGCTCTTCCTCTTCCTCTTGCGTCCGCATACCACGCGCCACTGGGTCGTCT  23 580
            ||||||||||||||||||||||||||||||||||||||||||||||||||||||||||||
2   23 521  GCTTCGCTGGGCTCTTCCTCTTCCTCTTGCGTCCGCATACCACGCGCCACTGGGTCGTCT  23 580

1   23 581  TCATTCAGCCGCCGCACTGTGCGCTTACCTCCTTTGCCATGCTTGATTAGCACCGGTGGG  23 640
            ||||||||||||||||||||||||||||||||||||||||||||||||||||||||||||
2   23 581  TCATTCAGCCGCCGCACTGTGCGCTTACCTCCTTTGCCATGCTTGATTAGCACCGGTGGG  23 640

1   23 641  TTGCTGAAACCCACCATTTGTAGCGCCACATCTTCTCTTTCTTCCTCGCTGTCCACGATT  23 700
            ||||||||||||||||||||||||||||||||||||||||||||||||||||||||||||
2   23 641  TTGCTGAAACCCACCATTTGTAGCGCCACATCTTCTCTTTCTTCCTCGCTGTCCACGATT  23 700

1   23 701  ACCTCTGGTGATGGCGGGCGCTCGGGCTTGGGAGAAGGGCGCTTCTTTTTCTTCTTGGGC  23 760
            ||||||||||||||||||||||||||||||||||||||||||||||||||||||||||||
2   23 701  ACCTCTGGTGATGGCGGGCGCTCGGGCTTGGGAGAAGGGCGCTTCTTTTTCTTCTTGGGC  23 760

1   23 761  GCAATGGCCAAATCCGCCGCCGAGGTCGATGGCCGCGGGCTGGGTGTGCGCGGCACCAGC  23 820
            ||||||||||||||||||||||||||||||||||||||||||||||||||||||||||||
2   23 761  GCAATGGCCAAATCCGCCGCCGAGGTCGATGGCCGCGGGCTGGGTGTGCGCGGCACCAGC  23 820

1   23 821  GCGTCTTGTGATGAGTCTTCCTCGTCCTCGGACTCGATACGCCGCCTCATCCGCTTTTTT  23 880
            ||||||||||||||||||||||||||||||||||||||||||||||||||||||||||||
2   23 821  GCGTCTTGTGATGAGTCTTCCTCGTCCTCGGACTCGATACGCCGCCTCATCCGCTTTTTT  23 880

1   23 881  GGGGGCGCCCGGGGAGGCGGCGGCGACGGGGACGGGGACGACACGTCCTCCATGGTTGGG  23 940
            ||||||||||||||||||||||||||||||||||||||||||||||||||||||||||||
2   23 881  GGGGGCGCCCGGGGAGGCGGCGGCGACGGGGACGGGGACGACACGTCCTCCATGGTTGGG  23 940
```

```
1   23 941  GGACGTCGCGCCGCACCGCGTCCGCGCTCGGGGGTGGTTTCGCGCTGCTCCTCTTCCCGA  24 000
            ||||||||||||||||||||||||||||||||||||||||||||||||||||||||||||
2   23 941  GGACGTCGCGCCGCACCGCGTCCGCGCTCGGGGGTGGTTTCGCGCTGCTCCTCTTCCCGA  24 000

1   24 001  CTGGCCATTTCCTTCTCCTATAGGCAGAAAAAGATCATGGAGTCAGTCGAGAAGAAGGAC  24 060
            ||||||||||||||||||||||||||||||||||||||||||||||||||||||||||||
2   24 001  CTGGCCATTTCCTTCTCCTATAGGCAGAAAAAGATCATGGAGTCAGTCGAGAAGAAGGAC  24 060

1   24 061  AGCCTAACCGCCCCCTCTGAGTTCGCCACCACCGCCTCCACCGATGCCGCCAACGCGCCT  24 120
            ||||||||||||||||||||||||||||||||||||||||||||||||||||||||||||
2   24 061  AGCCTAACCGCCCCCTCTGAGTTCGCCACCACCGCCTCCACCGATGCCGCCAACGCGCCT  24 120

1   24 121  ACCACCTTCCCCGTCGAGGCACCCCCGCTTGAGGAGGAGGAAGTGATTATCGAGCAGGAC  24 180
            ||||||||||||||||||||||||||||||||||||||||||||||||||||||||||||
2   24 121  ACCACCTTCCCCGTCGAGGCACCCCCGCTTGAGGAGGAGGAAGTGATTATCGAGCAGGAC  24 180

1   24 181  CCAGGTTTTGTAAGCGAAGACGACGAGGACCGCTCAGTACCAACAGAGGATAAAAAGCAA  24 240
            ||||||||||||||||||||||||||||||||||||||||||||||||||||||||||||
2   24 181  CCAGGTTTTGTAAGCGAAGACGACGAGGACCGCTCAGTACCAACAGAGGATAAAAAGCAA  24 240

1   24 241  GACCAGGACAACGCAGAGGCAAACGAGGAACAAGTCGGGCGGGGGGACGAAAGGCATGGC  24 300
            ||||||||||||||||||||||||||||||||||||||||||||||||||||||||||||
2   24 241  GACCAGGACAACGCAGAGGCAAACGAGGAACAAGTCGGGCGGGGGGACGAAAGGCATGGC  24 300

1   24 301  GACTACCTAGATGTGGGAGACGACGTGCTGTTGAAGCATCTGCAGCGCCAGTGCGCCATT  24 360
            ||||||||||||||||||||||||||||||||||||||||||||||||||||||||||||
2   24 301  GACTACCTAGATGTGGGAGACGACGTGCTGTTGAAGCATCTGCAGCGCCAGTGCGCCATT  24 360

1   24 361  ATCTGCGACGCGTTGCAAGAGCGCAGCGATGTGCCCCTCGCCATAGCGGATGTCAGCCTT  24 420
            ||||||||||||||||||||||||||||||||||||||||||||||||||||||||||||
2   24 361  ATCTGCGACGCGTTGCAAGAGCGCAGCGATGTGCCCCTCGCCATAGCGGATGTCAGCCTT  24 420

1   24 421  GCCTACGAACGCCACCTATTCTCACCGCGCGTACCCCCCAAACGCCAAGAAAACGGCACA  24 480
            ||||||||||||||||||||||||||||||||||||||||||||||||||||||||||||
2   24 421  GCCTACGAACGCCACCTATTCTCACCGCGCGTACCCCCCAAACGCCAAGAAAACGGCACA  24 480

1   24 481  TGCGAGCCCAACCCGCGCCTCAACTTCTACCCCGTATTTGCCGTGCCAGAGGTGCTTGCC  24 540
            ||||||||||||||||||||||||||||||||||||||||||||||||||||||||||||
2   24 481  TGCGAGCCCAACCCGCGCCTCAACTTCTACCCCGTATTTGCCGTGCCAGAGGTGCTTGCC  24 540
```

```
1   24 541  ACCTATCACATCTTTTTCCAAAACTGCAAGATACCCCTATCCTGCCGTGCCAACCGCAGC  24 600
            ||||||||||||||||||||||||||||||||||||||||||||||||||||||||||||
2   24 541  ACCTATCACATCTTTTTCCAAAACTGCAAGATACCCCTATCCTGCCGTGCCAACCGCAGC  24 600

1   24 601  CGAGCGGACAAGCAGCTGGCCTTGCGGCAGGGCGCTGTCATACCTGATATCGCCTCGCTC  24 660
            ||||||||||||||||||||||||||||||||||||||||||||||||||||||||||||
2   24 601  CGAGCGGACAAGCAGCTGGCCTTGCGGCAGGGCGCTGTCATACCTGATATCGCCTCGCTC  24 660

1   24 661  AACGAAGTGCCAAAAATCTTTGAGGGTCTTGGACGCGACGAGAAGCGCGCGGCAAACGCT  24 720
            ||||||||||||||||||||||||||||||||||||||||||||||||||||||||||||
2   24 661  AACGAAGTGCCAAAAATCTTTGAGGGTCTTGGACGCGACGAGAAGCGCGCGGCAAACGCT  24 720

1   24 721  CTGCAACAGGAAAACAGCGAAAATGAAAGTCACTCTGGAGTGTTGGTGGAACTCGAGGGT  24 780
            ||||||||||||||||||||||||||||||||||||||||||||||||||||||||||||
2   24 721  CTGCAACAGGAAAACAGCGAAAATGAAAGTCACTCTGGAGTGTTGGTGGAACTCGAGGGT  24 780

1   24 781  GACAACGCGCGCCTAGCCGTACTAAAACGCAGCATCGAGGTCACCCACTTTGCCTACCCG  24 840
            ||||||||||||||||||||||||||||||||||||||||||||||||||||||||||||
2   24 781  GACAACGCGCGCCTAGCCGTACTAAAACGCAGCATCGAGGTCACCCACTTTGCCTACCCG  24 840

1   24 841  GCACTTAACCTACCCCCCAAGGTCATGAGCACAGTCATGAGTGAGCTGATCGTGCGCCGT  24 900
            ||||||||||||||||||||||||||||||||||||||||||||||||||||||||||||
2   24 841  GCACTTAACCTACCCCCCAAGGTCATGAGCACAGTCATGAGTGAGCTGATCGTGCGCCGT  24 900

1   24 901  GCGCAGCCCCTGGAGAGGGATGCAAATTTGCAAGAACAAACAGAGGAGGGCCTACCCGCA  24 960
            ||||||||||||||||||||||||||||||||||||||||||||||||||||||||||||
2   24 901  GCGCAGCCCCTGGAGAGGGATGCAAATTTGCAAGAACAAACAGAGGAGGGCCTACCCGCA  24 960

1   24 961  GTTGGCGACGAGCAGCTAGCGCGCTGGCTTCAAACGCGCGAGCCTGCCGACTTGGAGGAG  25 020
            ||||||||||||||||||||||||||||||||||||||||||||||||||||||||||||
2   24 961  GTTGGCGACGAGCAGCTAGCGCGCTGGCTTCAAACGCGCGAGCCTGCCGACTTGGAGGAG  25 020

1   25 021  CGACGCAAACTAATGATGGCCGCAGTGCTCGTTACCGTGGAGCTTGAGTGCATGCAGCGG  25 080
            ||||||||||||||||||||||||||||||||||||||||||||||||||||||||||||
2   25 021  CGACGCAAACTAATGATGGCCGCAGTGCTCGTTACCGTGGAGCTTGAGTGCATGCAGCGG  25 080

1   25 081  TTCTTTGCTGACCCGGAGATGCAGCGCAAGCTAGAGGAAACATTGCACTACACCTTTCGA  25 140
            ||||||||||||||||||||||||||||||||||||||||||||||||||||||||||||
2   25 081  TTCTTTGCTGACCCGGAGATGCAGCGCAAGCTAGAGGAAACATTGCACTACACCTTTCGA  25 140
```

```
1   25 141  CAGGGCTACGTACGCCAGGCCTGCAAGATCTCCAACGTGGAGCTCTGCAACCTGGTCTCC  25 200
            ||||||||||||||||||||||||||||||||||||||||||||||||||||||||||||
2   25 141  CAGGGCTACGTACGCCAGGCCTGCAAGATCTCCAACGTGGAGCTCTGCAACCTGGTCTCC  25 200

1   25 201  TACCTTGGAATTTTGCACGAAAACCGCCTTGGGCAAAACGTGCTTCATTCCACGCTCAAG  25 260
            ||||||||||||||||||||||||||||||||||||||||||||||||||||||||||||
2   25 201  TACCTTGGAATTTTGCACGAAAACCGCCTTGGGCAAAACGTGCTTCATTCCACGCTCAAG  25 260

1   25 261  GGCGAGGCGCGCCGCGACTACGTCCGCGACTGCGTTTACTTATTTCTATGCTACACCTGG  25 320
            ||||||||||||||||||||||||||||||||||||||||||||||||||||||||||||
2   25 261  GGCGAGGCGCGCCGCGACTACGTCCGCGACTGCGTTTACTTATTTCTATGCTACACCTGG  25 320

1   25 321  CAGACGGCCATGGGCGTTTGGCAGCAGTGCTTGGAGGAGTGCAACCTCAAGGAGCTGCAG  25 380
            ||||||||||||||||||||||||||||||||||||||||||||||||||||||||||||
2   25 321  CAGACGGCCATGGGCGTTTGGCAGCAGTGCTTGGAGGAGTGCAACCTCAAGGAGCTGCAG  25 380

1   25 381  AAACTGCTAAAGCAAAACTTGAAGGACCTATGGACGGCCTTCAACGAGCGCTCCGTGGCC  25 440
            ||||||||||||||||||||||||||||||||||||||||||||||||||||||||||||
2   25 381  AAACTGCTAAAGCAAAACTTGAAGGACCTATGGACGGCCTTCAACGAGCGCTCCGTGGCC  25 440

1   25 441  GCGCACCTGGCGGACATCATTTTCCCCGAACGCCTGCTTAAAACCCTGCAACAGGGTCTG  25 500
            ||||||||||||||||||||||||||||||||||||||||||||||||||||||||||||
2   25 441  GCGCACCTGGCGGACATCATTTTCCCCGAACGCCTGCTTAAAACCCTGCAACAGGGTCTG  25 500

1   25 501  CCAGACTTCACCAGTCAAAGCATGTTGCAGAACTTTAGGAACTTTATCCTAGAGCGCTCA  25 560
            ||||||||||||||||||||||||||||||||||||||||||||||||||||||||||||
2   25 501  CCAGACTTCACCAGTCAAAGCATGTTGCAGAACTTTAGGAACTTTATCCTAGAGCGCTCA  25 560

1   25 561  GGAATCTTGCCCGCCACCTGCTGTGCACTTCCTAGCGACTTTGTGCCCATTAAGTACCGC  25 620
            ||||||||||||||||||||||||||||||||||||||||||||||||||||||||||||
2   25 561  GGAATCTTGCCCGCCACCTGCTGTGCACTTCCTAGCGACTTTGTGCCCATTAAGTACCGC  25 620

1   25 621  GAATGCCCTCCGCCGCTTTGGGGCCACTGCTACCTTCTGCAGCTAGCCAACTACCTTGCC  25 680
            ||||||||||||||||||||||||||||||||||||||||||||||||||||||||||||
2   25 621  GAATGCCCTCCGCCGCTTTGGGGCCACTGCTACCTTCTGCAGCTAGCCAACTACCTTGCC  25 680

1   25 681  TACCACTCTGACATAATGGAAGACGTGAGCGGTGACGGTCTACTGGAGTGTCACTGTCGC  25 740
            ||||||||||||||||||||||||||||||||||||||||||||||||||||||||||||
2   25 681  TACCACTCTGACATAATGGAAGACGTGAGCGGTGACGGTCTACTGGAGTGTCACTGTCGC  25 740
```

```
1   25 741  TGCAACCTATGCACCCCGCACCGCTCCCTGGTTTGCAATTCGCAGCTGCTTAACGAAAGT  25 800
            ||||||||||||||||||||||||||||||||||||||||||||||||||||||||||||
2   25 741  TGCAACCTATGCACCCCGCACCGCTCCCTGGTTTGCAATTCGCAGCTGCTTAACGAAAGT  25 800

1   25 801  CAAATTATCGGTACCTTTGAGCTGCAGGGTCCCTCGCCTGACGAAAAGTCCGCGGCTCCG  25 860
            ||||||||||||||||||||||||||||||||||||||||||||||||||||||||||||
2   25 801  CAAATTATCGGTACCTTTGAGCTGCAGGGTCCCTCGCCTGACGAAAAGTCCGCGGCTCCG  25 860

1   25 861  GGGTTGAAACTCACTCCGGGGCTGTGGACGTCGGCTTACCTTCGCAAATTTGTACCTGAG  25 920
            ||||||||||||||||||||||||||||||||||||||||||||||||||||||||||||
2   25 861  GGGTTGAAACTCACTCCGGGGCTGTGGACGTCGGCTTACCTTCGCAAATTTGTACCTGAG  25 920

1   25 921  GACTACCACGCCCACGAGATTAGGTTCTACGAAGACCAATCCCGCCCGCCTAATGCGGAG  25 980
            ||||||||||||||||||||||||||||||||||||||||||||||||||||||||||||
2   25 921  GACTACCACGCCCACGAGATTAGGTTCTACGAAGACCAATCCCGCCCGCCTAATGCGGAG  25 980

1   25 981  CTTACCGCCTGCGTCATTACCCAGGGCCACATTCTTGGCCAATTGCAAGCCATCAACAAA  26 040
            ||||||||||||||||||||||||||||||||||||||||||||||||||||||||||||
2   25 981  CTTACCGCCTGCGTCATTACCCAGGGCCACATTCTTGGCCAATTGCAAGCCATCAACAAA  26 040

1   26 041  GCCCGCCAAGAGTTTCTGCTACGAAAGGGACGGGGGGTTTACTTGGACCCCCAGTCCGGC  26 100
            ||||||||||||||||||||||||||||||||||||||||||||||||||||||||||||
2   26 041  GCCCGCCAAGAGTTTCTGCTACGAAAGGGACGGGGGGTTTACTTGGACCCCCAGTCCGGC  26 100

1   26 101  GAGGAGCTCAACCCAATCCCCCCGCCGCCGCAGCCCTATCAGCAGCAGCCGCGGGCCCTT  26 160
            ||||||||||||||||||||||||||||||||||||||||||||||||||||||||||||
2   26 101  GAGGAGCTCAACCCAATCCCCCCGCCGCCGCAGCCCTATCAGCAGCAGCCGCGGGCCCTT  26 160

1   26 161  GCTTCCCAGGATGGCACCCAAAAAGAAGCTGCAGCTGCCGCCGCCACCCACGGACGAGGA  26 220
            ||||||||||||||||||||||||||||||||||||||||||||||||||||||||||||
2   26 161  GCTTCCCAGGATGGCACCCAAAAAGAAGCTGCAGCTGCCGCCGCCACCCACGGACGAGGA  26 220

1   26 221  GGAATACTGGGACAGTCAGGCAGAGGAGGTTTTGGACGAGGAGGAGGAGGACATGATGGA  26 280
            ||||||||||||||||||||||||||||||||||||||||||||||||||||||||||||
2   26 221  GGAATACTGGGACAGTCAGGCAGAGGAGGTTTTGGACGAGGAGGAGGAGGACATGATGGA  26 280

1   26 281  AGACTGGGAGAGCCTAGACGAGGAAGCTTCCGAGGTCGAAGAGGTGTCAGACGAAACACC  26 340
            ||||||||||||||||||||||||||||||||||||||||||||||||||||||||||||
2   26 281  AGACTGGGAGAGCCTAGACGAGGAAGCTTCCGAGGTCGAAGAGGTGTCAGACGAAACACC  26 340
```

```
1  26 341  GTCACCCTCGGTCGCATTCCCCTCGCCGGCGCCCCAGAAATCGGCAACCGGTTCCAGCAT  26 400
           ||||||||||||||||||||||||||||||||||||||||||||||||||||||||||||
2  26 341  GTCACCCTCGGTCGCATTCCCCTCGCCGGCGCCCCAGAAATCGGCAACCGGTTCCAGCAT  26 400

1  26 401  GGCTACAACCTCCGCTCCTCAGGCGCCGCCGGCACTGCCCGTTCGCCGACCCAACCGTAG  26 460
           ||||||||||||||||||||||||||||||||||||||||||||||||||||||||||||
2  26 401  GGCTACAACCTCCGCTCCTCAGGCGCCGCCGGCACTGCCCGTTCGCCGACCCAACCGTAG  26 460

1  26 461  ATGGGACACCACTGGAACCAGGGCCGGTAAGTCCAAGCAGCCGCCGCCGTTAGCCCAAGA  26 520
           ||||||||||||||||||||||||||||||||||||||||||||||||||||||||||||
2  26 461  ATGGGACACCACTGGAACCAGGGCCGGTAAGTCCAAGCAGCCGCCGCCGTTAGCCCAAGA  26 520

1  26 521  GCAACAACAGCGCCAAGGCTACCGCTCATGGCGCGGGCACAAGAACGCCATAGTTGCTTG  26 580
           ||||||||||||||||||||||||||||||||||||||||||||||||||||||||||||
2  26 521  GCAACAACAGCGCCAAGGCTACCGCTCATGGCGCGGGCACAAGAACGCCATAGTTGCTTG  26 580

1  26 581  CTTGCAAGACTGTGGGGGCAACATCTCCTTCGCCCGCCGCTTTCTTCTCTACCATCACGG  26 640
           ||||||||||||||||||||||||||||||||||||||||||||||||||||||||||||
2  26 581  CTTGCAAGACTGTGGGGGCAACATCTCCTTCGCCCGCCGCTTTCTTCTCTACCATCACGG  26 640

1  26 641  CGTGGCCTTCCCCCGTAACATCCTGCATTACTACCGTCATCTCTACAGCCCATACTGCAC  26 700
           ||||||||||||||||||||||||||||||||||||||||||||||||||||||||||||
2  26 641  CGTGGCCTTCCCCCGTAACATCCTGCATTACTACCGTCATCTCTACAGCCCATACTGCAC  26 700

1  26 701  CGGCGGCAGCGGCAGCAACAGCAGCGGCCACACAGAAGCAAAGGCGACCGGATAGCAAGA  26 760
           ||||||||||||||||||||||||||||||||||||||||||||||||||||||||||||
2  26 701  CGGCGGCAGCGGCAGCAACAGCAGCGGCCACACAGAAGCAAAGGCGACCGGATAGCAAGA  26 760

1  26 761  CTCTGACAAAGCCCAAGAAATCCACAGCGGCGGCAGCAGCAGGAGGAGGAGCGCTGCGTC  26 820
           ||||||||||||||||||||||||||||||||||||||||||||||||||||||||||||
2  26 761  CTCTGACAAAGCCCAAGAAATCCACAGCGGCGGCAGCAGCAGGAGGAGGAGCGCTGCGTC  26 820

1  26 821  TGGCGCCCAACGAACCCGTATCGACCCGCGAGCTTAGAAACAGGATTTTTCCCACTCTGT  26 880
           ||||||||||||||||||||||||||||||||||||||||||||||||||||||||||||
2  26 821  TGGCGCCCAACGAACCCGTATCGACCCGCGAGCTTAGAAACAGGATTTTTCCCACTCTGT  26 880

1  26 881  ATGCTATATTTCAACAGAGCAGGGGCCAAGAACAAGAGCTGAAAATAAAAAACAGGTCTC  26 940
           ||||||||||||||||||||||||||||||||||||||||||||||||||||||||||||
2  26 881  ATGCTATATTTCAACAGAGCAGGGGCCAAGAACAAGAGCTGAAAATAAAAAACAGGTCTC  26 940
```

```
1   26 941  TGCGATCCCTCACCCGCAGCTGCCTGTATCACAAAAGCGAAGATCAGCTTCGGCGCACGC  27 000
            ||||||||||||||||||||||||||||||||||||||||||||||||||||||||||||
2   26 941  TGCGATCCCTCACCCGCAGCTGCCTGTATCACAAAAGCGAAGATCAGCTTCGGCGCACGC  27 000

1   27 001  TGGAAGACGCGGAGGCTCTCTTCAGTAAATACTGCGCGCTGACTCTTAAGGACTAGTTTC  27 060
            ||||||||||||||||||||||||||||||||||||||||||||||||||||||||||||
2   27 001  TGGAAGACGCGGAGGCTCTCTTCAGTAAATACTGCGCGCTGACTCTTAAGGACTAGTTTC  27 060

1   27 061  GCGCCCTTTCTCAAATTTAAGCGCGAAAACTACGTCATCTCCAGCGGCCACACCCGGCGC  27 120
            ||||||||||||||||||||||||||||||||||||||||||||||||||||||||||||
2   27 061  GCGCCCTTTCTCAAATTTAAGCGCGAAAACTACGTCATCTCCAGCGGCCACACCCGGCGC  27 120

1   27 121  CAGCACCTGTTGTCAGCGCCATTATGAGCAAGGAAATTCCCACGCCCTACATGTGGAGTT  27 180
            ||||||||||||||||||||||||||||||||||||||||||||||||||||||||||||
2   27 121  CAGCACCTGTTGTCAGCGCCATTATGAGCAAGGAAATTCCCACGCCCTACATGTGGAGTT  27 180

1   27 181  ACCAGCCACAAATGGGACTTGCGGCTGGAGCTGCCCAAGACTACTCAACCCGAATAAACT  27 240
            ||||||||||||||||||||||||||||||||||||||||||||||||||||||||||||
2   27 181  ACCAGCCACAAATGGGACTTGCGGCTGGAGCTGCCCAAGACTACTCAACCCGAATAAACT  27 240

1   27 241  ACATGAGCGCGGGACCCCACATGATATCCCGGGTCAACGGAATACGCGCCCACCGAAACC  27 300
            ||||||||||||||||||||||||||||||||||||||||||||||||||||||||||||
2   27 241  ACATGAGCGCGGGACCCCACATGATATCCCGGGTCAACGGAATACGCGCCCACCGAAACC  27 300

1   27 301  GAATTCTCCTGGAACAGGCGGCTATTACCACCACACCTCGTAATAACCTTAATCCCCGTA  27 360
            ||||||||||||||||||||||||||||||||||||||||||||||||||||||||||||
2   27 301  GAATTCTCCTGGAACAGGCGGCTATTACCACCACACCTCGTAATAACCTTAATCCCCGTA  27 360

1   27 361  GTTGGCCCGCTGCCCTGGTGTACCAGGAAAGTCCCGCTCCCACCACTGTGGTACTTCCCA  27 420
            ||||||||||||||||||||||||||||||||||||||||||||||||||||||||||||
2   27 361  GTTGGCCCGCTGCCCTGGTGTACCAGGAAAGTCCCGCTCCCACCACTGTGGTACTTCCCA  27 420

1   27 421  GAGACGCCCAGGCCGAAGTTCAGATGACTAACTCAGGGGCGCAGCTTGCGGGCGGCTTTC  27 480
            ||||||||||||||||||||||||||||||||||||||||||||||||||||||||||||
2   27 421  GAGACGCCCAGGCCGAAGTTCAGATGACTAACTCAGGGGCGCAGCTTGCGGGCGGCTTTC  27 480

1   27 481  GTCACAGGGTGCGGTCGCCCGGGCAGGGTATAACTCACCTGACAATCAGAGGGCGAGGTA  27 540
            ||||||||||||||||||||||||||||||||||||||||||||||||||||||||||||
2   27 481  GTCACAGGGTGCGGTCGCCCGGGCAGGGTATAACTCACCTGACAATCAGAGGGCGAGGTA  27 540
```

```
1   27 541   TTCAGCTCAACGACGAGTCGGTGAGCTCCTCGCTTGGTCTCCGTCCGGACGGGACATTTC   27 600
             ||||||||||||||||||||||||||||||||||||||||||||||||||||||||||||
2   27 541   TTCAGCTCAACGACGAGTCGGTGAGCTCCTCGCTTGGTCTCCGTCCGGACGGGACATTTC   27 600

1   27 601   AGATCGGCGGCGCCGGCCGCTCTTCATTCACGCCTCGTCAGGCAATCCTAACTCTGCAGA   27 660
             ||||||||||||||||||||||||||||||||||||||||||||||||||||||||||||
2   27 601   AGATCGGCGGCGCCGGCCGCTCTTCATTCACGCCTCGTCAGGCAATCCTAACTCTGCAGA   27 660

1   27 661   CCTCGTCCTCTGAGCCGCGCTCTGGAGGCATTGGAACTCTGCAATTTATTGAGGAGTTTG   27 720
             ||||||||||||||||||||||||||||||||||||||||||||||||||||||||||||
2   27 661   CCTCGTCCTCTGAGCCGCGCTCTGGAGGCATTGGAACTCTGCAATTTATTGAGGAGTTTG   27 720

1   27 721   TGCCATCGGTCTACTTTAACCCCTTCTCGGGACCTCCCGGCCACTATCCGGATCAATTTA   27 780
             ||||||||||||||||||||||||||||||||||||||||||||||||||||||||||||
2   27 721   TGCCATCGGTCTACTTTAACCCCTTCTCGGGACCTCCCGGCCACTATCCGGATCAATTTA   27 780

1   27 781   TTCCTAACTTTGACGCGGTAAAGGACTCGGCGGACGGCTACGACTGAATGTTAAGTGGAG   27 840
             ||||||||||||||||||||||||||||||||||||||||||||||||||||||||||||
2   27 781   TTCCTAACTTTGACGCGGTAAAGGACTCGGCGGACGGCTACGACTGAATGTTAAGTGGAG   27 840

1   27 841   AGGCAGAGCAACTGCGCCTGAAACACCTGGTCCACTGTCGCCGCCACAAGTGCTTTGCCC   27 900
             ||||||||||||||||||||||||||||||||||||||||||||||||||||||||||||
2   27 841   AGGCAGAGCAACTGCGCCTGAAACACCTGGTCCACTGTCGCCGCCACAAGTGCTTTGCCC   27 900

1   27 901   GCGACTCCGGTGAGTTTTGCTACTTTGAATTGCCCGAGGATCATATCGAGGGCCCGGCGC   27 960
             ||||||||||||||||||||||||||||||||||||||||||||||||||||||||||||
2   27 901   GCGACTCCGGTGAGTTTTGCTACTTTGAATTGCCCGAGGATCATATCGAGGGCCCGGCGC   27 960

1   27 961   ACGGCGTCCGGCTTACCGCCCAGGGAGAGCTTGCCCGTAGCCTGATTCGGGAGTTTACCC   28 020
             ||||||||||||||||||||||||||||||||||||||||||||||||||||||||||||
2   27 961   ACGGCGTCCGGCTTACCGCCCAGGGAGAGCTTGCCCGTAGCCTGATTCGGGAGTTTACCC   28 020

1   28 021   AGCGCCCCCTGCTAGTTGAGCGGGACAGGGGACCCTGTGTTCTCACTGTGATTTGCAACT   28 080
             ||||||||||||||||||||||||||||||||||||||||||||||||||||||||||||
2   28 021   AGCGCCCCCTGCTAGTTGAGCGGGACAGGGGACCCTGTGTTCTCACTGTGATTTGCAACT   28 080

1   28 081   GTCCTAACCCTGGATTACATCAAGATCTTTGTTGCCATCTCTGTGCTGAGTATAATAAAT   28 140
             ||||||||||||||||||||||||||||||||||||||||||||||||||||||||||||
2   28 081   GTCCTAACCCTGGATTACATCAAGATCTTTGTTGCCATCTCTGTGCTGAGTATAATAAAT   28 140
```

```
1   28 141  ACAGAAATTAAAATATACTGGGGCTCCTATCGCCATCCTGTAAACGCCACCGTCTTCACC  28 200
            ||||||||||||||||||||||||||||||||||||||||||||||||||||||||||||
2   28 141  ACAGAAATTAAAATATACTGGGGCTCCTATCGCCATCCTGTAAACGCCACCGTCTTCACC  28 200

1   28 201  CGCCCAAGCAAACCAAGGCGAACCTTACCTGGTACTTTTAACATCTCTCCCTCTGTGATT  28 260
            ||||||||||||||||||||||||||||||||||||||||||||||||||||||||||||
2   28 201  CGCCCAAGCAAACCAAGGCGAACCTTACCTGGTACTTTTAACATCTCTCCCTCTGTGATT  28 260

1   28 261  TACAACAGTTTCAACCCAGACGGAGTGAGTCTACGAGAGAACCTCTCCGAGCTCAGCTAC  28 320
            ||||||||||||||||||||||||||||||||||||||||||||||||||||||||||||
2   28 261  TACAACAGTTTCAACCCAGACGGAGTGAGTCTACGAGAGAACCTCTCCGAGCTCAGCTAC  28 320

1   28 321  TCCATCAGAAAAAACACCACCCTCCTTACCTGCCGGGAACGTACGAGTGCGTCACCGGCC  28 380
            ||||||||||||||||||||||||||||||||||||||||||||||||||||||||||||
2   28 321  TCCATCAGAAAAAACACCACCCTCCTTACCTGCCGGGAACGTACGAGTGCGTCACCGGCC  28 380

1   28 381  GCTGCACCACACCTACCGCCTGACCGTAAACCAGACTTTTTCCGGACAGACCTCAATAAC  28 440
            ||||||||||||||||||||||||||||||||||||||||||||||||||||||||||||
2   28 381  GCTGCACCACACCTACCGCCTGACCGTAAACCAGACTTTTTCCGGACAGACCTCAATAAC  28 440

1   28 441  TCTGTTTACCAGAACAGGAGGTGAGCTTAGAAAACCCTTAGGGTATTAGGCCAAAGGCGC  28 500
            ||||||||||||||||||||||||||||||||||||||||||||||||||||||||||||
2   28 441  TCTGTTTACCAGAACAGGAGGTGAGCTTAGAAAACCCTTAGGGTATTAGGCCAAAGGCGC  28 500

1   28 501  AGCTACTGTGGGGTTTACCATGGAAAGGGTCCAACCCCTGGAAGAGAATGTGGGAAATGC  28 560
            ||||||||||||||||||||||||||||||||||||||||||||||||||||||||||||
2   28 501  AGCTACTGTGGGGTTTACCATGGAAAGGGTCCAACCCCTGGAAGAGAATGTGGGAAATGC  28 560

1   28 561  AGCCAGGCCAAGATTCGAGAGGAACAAGCTATTGCTGGTGGCCTCTGTAATTCAGGGACT  28 620
            ||||||||||||||||||||||||||||||||||||||||||||||||||||||||||||
2   28 561  AGCCAGGCCAAGATTCGAGAGGAACAAGCTATTGCTGGTGGCCTCTGTAATTCAGGGACT  28 620

1   28 621  GGGGCTGCTCCTGTGCTTCACCTACATCTGCCTGCACTTCTCTGCTCTTCAGGTATCACA  28 680
            ||||||||||||||||||||||||||||||||||||||||||||||||||||||||||||
2   28 621  GGGGCTGCTCCTGTGCTTCACCTACATCTGCCTGCACTTCTCTGCTCTTCAGGTATCACA  28 680

1   28 681  TCGGTATCCTCGAATTCAAAGTATCAAAGTACAATTTACCGAATATAAGAAGGAGAAAGG  28 740
            ||||||||||||||||||||||||||||||||||||||||||||||||||||||||||||
2   28 681  TCGGTATCCTCGAATTCAAAGTATCAAAGTACAATTTACCGAATATAAGAAGGAGAAAGG  28 740
```

```
1   28 741  TTTCATCCTCACTTCCCAAAAGGAGGATGAAATCATGAAGGTGCAGAACAACTCAGTCAT  28 800
            ||||||||||||||||||||||||||||||||||||||||||||||||||||||||||||
2   28 741  TTTCATCCTCACTTCCCAAAAGGAGGATGAAATCATGAAGGTGCAGAACAACTCAGTCAT  28 800

1   28 801  CATCAACTGTGATGGGTTTTATCTCATCTCCCTGAAGGGCTACTTCTCCCAGGAAGTCAA  28 860
            ||||||||||||||||||||||||||||||||||||||||||||||||||||||||||||
2   28 801  CATCAACTGTGATGGGTTTTATCTCATCTCCCTGAAGGGCTACTTCTCCCAGGAAGTCAA  28 860

1   28 861  CATTAGCCTTCATTACCAGAAGGATGAGGAGCCCCTCTTCCAACTGAAGAAGGTCAGGTC  28 920
            ||||||||||||||||||||||||||||||||||||||||||||||||||||||||||||
2   28 861  CATTAGCCTTCATTACCAGAAGGATGAGGAGCCCCTCTTCCAACTGAAGAAGGTCAGGTC  28 920

1   28 921  TGTCAACTCCTTGATGGTGGCCTCTCTGACTTACAAAGACAAAGTCTACTTGAATGTGAC  28 980
            ||||||||||||||||||||||||||||||||||||||||||||||||||||||||||||
2   28 921  TGTCAACTCCTTGATGGTGGCCTCTCTGACTTACAAAGACAAAGTCTACTTGAATGTGAC  28 980

1   28 981  CACTGACAATACCTCCCTGGATGACTTCCATGTGAATGGCGGAGAACTGATTCTTATCCA  29 040
            ||||||||||||||||||||||||||||||||||||||||||||||||||||||||||||
2   28 981  CACTGACAATACCTCCCTGGATGACTTCCATGTGAATGGCGGAGAACTGATTCTTATCCA  29 040

1   29 041  TCAAAATCCTGGTGAATTCTGTGTCCTTAGGAAGAGGAGGAGCGGCAGCGGCGAGGCCAG  29 100
            ||||||||||||||||||||||||||||||||||||||||||||||||||||||||||||
2   29 041  TCAAAATCCTGGTGAATTCTGTGTCCTTAGGAAGAGGAGGAGCGGCAGCGGCGAGGCCAG  29 100

1   29 101  GCACAAGCAGAAGATCGTGGCCCCCGTGAAGCAGACCCTGAACTTCGACCTGCTGAAGCT  29 160
            ||||||||||||||||||||||||||||||||||||||||||||||||||||||||||||
2   29 101  GCACAAGCAGAAGATCGTGGCCCCCGTGAAGCAGACCCTGAACTTCGACCTGCTGAAGCT  29 160

1   29 161  GGCCGGCGACGTGGAGAGCAACCCCGGCCCCATGATCGAAACATACAACCAAACTTCTCC  29 220
            ||||||||||||||||||||||||||||||||||||||||||||||||||||||||||||
2   29 161  GGCCGGCGACGTGGAGAGCAACCCCGGCCCCATGATCGAAACATACAACCAAACTTCTCC  29 220

1   29 221  CCGATCTGCGGCCACTGGACTGCCCATCAGCATGAAAATTTTTATGTATTTACTTACTGT  29 280
            ||||||||||||||||||||||||||||||||||||||||||||||||||||||||||||
2   29 221  CCGATCTGCGGCCACTGGACTGCCCATCAGCATGAAAATTTTTATGTATTTACTTACTGT  29 280

1   29 281  TTTTCTTATCACCCAGATGATTGGGTCAGCACTTTTTGCTGTGTATCTTCATAGAAGGTT  29 340
            ||||||||||||||||||||||||||||||||||||||||||||||||||||||||||||
2   29 281  TTTTCTTATCACCCAGATGATTGGGTCAGCACTTTTTGCTGTGTATCTTCATAGAAGGTT  29 340
```

```
1   29 341  GGACAAGATAGAAGATGAAAGGAATCTTCATGAAGATTTTGTATTCATGAAAACGATACA  29 400
            ||||||||||||||||||||||||||||||||||||||||||||||||||||||||||||
2   29 341  GGACAAGATAGAAGATGAAAGGAATCTTCATGAAGATTTTGTATTCATGAAAACGATACA  29 400

1   29 401  GAGATGCAACACAGGAGAAAGATCCTTATCCTTACTGAACTGTGAGGAGATTAAAAGCCA  29 460
            ||||||||||||||||||||||||||||||||||||||||||||||||||||||||||||
2   29 401  GAGATGCAACACAGGAGAAAGATCCTTATCCTTACTGAACTGTGAGGAGATTAAAAGCCA  29 460

1   29 461  GTTTGAAGGCTTTGTGAAGGATATAATGTTAAACAAAGAGGAGACGAAGAAAGAAAACAG  29 520
            ||||||||||||||||||||||||||||||||||||||||||||||||||||||||||||
2   29 461  GTTTGAAGGCTTTGTGAAGGATATAATGTTAAACAAAGAGGAGACGAAGAAAGAAAACAG  29 520

1   29 521  CTTTGAAATGCAAAAAGGTGATCAGAATCCTCAAATTGCGGCACATGTCATAAGTGAGGC  29 580
            ||||||||||||||||||||||||||||||||||||||||||||||||||||||||||||
2   29 521  CTTTGAAATGCAAAAAGGTGATCAGAATCCTCAAATTGCGGCACATGTCATAAGTGAGGC  29 580

1   29 581  CAGCAGTAAAACAACATCTGTGTTACAGTGGGCTGAAAAAGGATACTACACCATGAGCAA  29 640
            ||||||||||||||||||||||||||||||||||||||||||||||||||||||||||||
2   29 581  CAGCAGTAAAACAACATCTGTGTTACAGTGGGCTGAAAAAGGATACTACACCATGAGCAA  29 640

1   29 641  CAACTTGGTAACCCTGGAAAATGGGAAACAGCTGACCGTTAAAAGACAAGGACTCTATTA  29 700
            ||||||||||||||||||||||||||||||||||||||||||||||||||||||||||||
2   29 641  CAACTTGGTAACCCTGGAAAATGGGAAACAGCTGACCGTTAAAAGACAAGGACTCTATTA  29 700

1   29 701  TATCTATGCCCAAGTCACCTTCTGTTCCAATCGGGAAGCTTCGAGTCAAGCTCCATTTAT  29 760
            ||||||||||||||||||||||||||||||||||||||||||||||||||||||||||||
2   29 701  TATCTATGCCCAAGTCACCTTCTGTTCCAATCGGGAAGCTTCGAGTCAAGCTCCATTTAT  29 760

1   29 761  AGCCAGCCTCTGCCTAAAGTCCCCCGGTAGATTCGAGAGAATCTTACTCAGAGCTGCAAA  29 820
            ||||||||||||||||||||||||||||||||||||||||||||||||||||||||||||
2   29 761  AGCCAGCCTCTGCCTAAAGTCCCCCGGTAGATTCGAGAGAATCTTACTCAGAGCTGCAAA  29 820

1   29 821  TACCCACAGTTCCGCCAAACCTTGCGGGCAACAATCCATTCACTTGGGAGGAGTATTTGA  29 880
            ||||||||||||||||||||||||||||||||||||||||||||||||||||||||||||
2   29 821  TACCCACAGTTCCGCCAAACCTTGCGGGCAACAATCCATTCACTTGGGAGGAGTATTTGA  29 880

1   29 881  ATTGCAACCAGGTGCTTCGGTGTTTGTCAATGTGACTGATCCAAGCCAAGTGAGCCATGG  29 940
            ||||||||||||||||||||||||||||||||||||||||||||||||||||||||||||
2   29 881  ATTGCAACCAGGTGCTTCGGTGTTTGTCAATGTGACTGATCCAAGCCAAGTGAGCCATGG  29 940
```

```
1   29 941  CACTGGCTTCACGTCCTTTGGCTTACTCAAACTCTGATAATTTACTAAGTTACAAAGCTA  30 000
            ||||||||||||||||||||||||||||||||||||||||||||||||||||||||||||
2   29 941  CACTGGCTTCACGTCCTTTGGCTTACTCAAACTCTGATAATTTACTAAGTTACAAAGCTA  30 000

1   30 001  ATGTCACCACTAACTGCTTTACTCGCTGCTTGCAAAACAAATTCAAAAAGTTAGCATTAT  30 060
            ||||||||||||||||||||||||||||||||||||||||||||||||||||||||||||
2   30 001  ATGTCACCACTAACTGCTTTACTCGCTGCTTGCAAAACAAATTCAAAAAGTTAGCATTAT  30 060

1   30 061  AATTAGAATAGGATTTAAACCCCCCGGTCATTTCCTGCTCAATACCATTCCCCTGAACAA  30 120
            ||||||||||||||||||||||||||||||||||||||||||||||||||||||||||||
2   30 061  AATTAGAATAGGATTTAAACCCCCCGGTCATTTCCTGCTCAATACCATTCCCCTGAACAA  30 120

1   30 121  TTGACTCTATGTGGGATATGCTCCAGCGCTACAACCTTGAAGTCAGGCTTCCTGGATGTC  30 180
            ||||||||||||||||||||||||||||||||||||||||||||||||||||||||||||
2   30 121  TTGACTCTATGTGGGATATGCTCCAGCGCTACAACCTTGAAGTCAGGCTTCCTGGATGTC  30 180

1   30 181  AGCATCTGACTTTGGCCAGCACCTGTCCCGCGGATTTGTTCCAGTCCAACTACAGCGACC  30 240
            ||||||||||||||||||||||||||||||||||||||||||||||||||||||||||||
2   30 181  AGCATCTGACTTTGGCCAGCACCTGTCCCGCGGATTTGTTCCAGTCCAACTACAGCGACC  30 240

1   30 241  CACCCTAACAGAGATGACCAACACAACCAACGCGGCCGCCGCTACCGGACTTACATCTAC  30 300
            ||||||||||||||||||||||||||||||||||||||||||||||||||||||||||||
2   30 241  CACCCTAACAGAGATGACCAACACAACCAACGCGGCCGCCGCTACCGGACTTACATCTAC  30 300

1   30 301  CACAAATACACCCCAAGTTTCTGCCTTTGTCAATAACTGGGATAACTTGGGCATGTGGTG  30 360
            ||||||||||||||||||||||||||||||||||||||||||||||||||||||||||||
2   30 301  CACAAATACACCCCAAGTTTCTGCCTTTGTCAATAACTGGGATAACTTGGGCATGTGGTG  30 360

1   30 361  GTTCTCCATAGCGCTTATGTTTGTATGCCTTATTATTATGTGGCTCATCTGCTGCCTAAA  30 420
            ||||||||||||||||||||||||||||||||||||||||||||||||||||||||||||
2   30 361  GTTCTCCATAGCGCTTATGTTTGTATGCCTTATTATTATGTGGCTCATCTGCTGCCTAAA  30 420

1   30 421  GCGCAAACGCGCCCGACCACCCATCTATAGTCCCATCATTGTGCTACACCCAAACAATGA  30 480
            ||||||||||||||||||||||||||||||||||||||||||||||||||||||||||||
2   30 421  GCGCAAACGCGCCCGACCACCCATCTATAGTCCCATCATTGTGCTACACCCAAACAATGA  30 480

1   30 481  TGGAATCCATAGATTGGACGGACTGAAACACATGTTCTTTTCTCTTACAGTATGATTAAA  30 540
            ||||||||||||||||||||||||||||||||||||||||||||||||||||||||||||
2   30 481  TGGAATCCATAGATTGGACGGACTGAAACACATGTTCTTTTCTCTTACAGTATGATTAAA  30 540
```

```
1   30 541  TGAGACATGATTCCTCGAGTTTTTATATTACTGACCCTTGTTGCGCTTTTTTGTGCGTGC  30 600
            ||||||||||||||||||||||||||||||||||||||||||||||||||||||||||||
2   30 541  TGAGACATGATTCCTCGAGTTTTTATATTACTGACCCTTGTTGCGCTTTTTTGTGCGTGC  30 600

1   30 601  TCCACATTGGCTGCGGTTTCTCACATCGAAGTAGACTGCATTCCAGCCTTCACAGTCTAT  30 660
            ||||||||||||||||||||||||||||||||||||||||||||||||||||||||||||
2   30 601  TCCACATTGGCTGCGGTTTCTCACATCGAAGTAGACTGCATTCCAGCCTTCACAGTCTAT  30 660

1   30 661  TTGCTTTACGGATTTGTCACCCTCACGCTCATCTGCAGCCTCATCACTGTGGTCATCGCC  30 720
            ||||||||||||||||||||||||||||||||||||||||||||||||||||||||||||
2   30 661  TTGCTTTACGGATTTGTCACCCTCACGCTCATCTGCAGCCTCATCACTGTGGTCATCGCC  30 720

1   30 721  TTTATCCAGTGCATTGACTGGGTCTGTGTGCGCTTTGCATATCTCAGACACCATCCCCAG  30 780
            ||||||||||||||||||||||||||||||||||||||||||||||||||||||||||||
2   30 721  TTTATCCAGTGCATTGACTGGGTCTGTGTGCGCTTTGCATATCTCAGACACCATCCCCAG  30 780

1   30 781  TACAGGGACAGGACTATAGCTGAGCTTCTTAGAATTCTTTAATTATGAAATTTACTGTGA  30 840
            ||||||||||||||||||||||||||||||||||||||||||||||||||||||||||||
2   30 781  TACAGGGACAGGACTATAGCTGAGCTTCTTAGAATTCTTTAATTATGAAATTTACTGTGA  30 840

1   30 841  CTTTTCTGCTGATTATTTGCACCCTATCTGCGTTTTGTTCCCCGACCTCCAAGCCTCAAA  30 900
            ||||||||||||||||||||||||||||||||||||||||||||||||||||||||||||
2   30 841  CTTTTCTGCTGATTATTTGCACCCTATCTGCGTTTTGTTCCCCGACCTCCAAGCCTCAAA  30 900

1   30 901  GACATATATCATGCAGATTCACTCGTATATGGAATATTCCAAGTTGCTACAATGAAAAAA  30 960
            ||||||||||||||||||||||||||||||||||||||||||||||||||||||||||||
2   30 901  GACATATATCATGCAGATTCACTCGTATATGGAATATTCCAAGTTGCTACAATGAAAAAA  30 960

1   30 961  GCGATCTTTCCGAAGCCTGGTTATATGCAATCATCTCTGTTATGGTGTTCTGCAGTACCA  31 020
            ||||||||||||||||||||||||||||||||||||||||||||||||||||||||||||
2   30 961  GCGATCTTTCCGAAGCCTGGTTATATGCAATCATCTCTGTTATGGTGTTCTGCAGTACCA  31 020

1   31 021  TCTTAGCCCTAGCTATATATCCCTACCTTGACATTGGCTGGAACGCAATAGATGCCATGA  31 080
            ||||||||||||||||||||||||||||||||||||||||||||||||||||||||||||
2   31 021  TCTTAGCCCTAGCTATATATCCCTACCTTGACATTGGCTGGAACGCAATAGATGCCATGA  31 080

1   31 081  ACCACCCAACTTTCCCCGCGCCCGCTATGCTTCCACTGCAACAAGTTGTTGCCGGCGGCT  31 140
            ||||||||||||||||||||||||||||||||||||||||||||||||||||||||||||
2   31 081  ACCACCCAACTTTCCCCGCGCCCGCTATGCTTCCACTGCAACAAGTTGTTGCCGGCGGCT  31 140
```

```
1   31 141  TTGTCCCAGCCAATCAGCCTCGCCCACCTTCTCCCACCCCCACTGAAATCAGCTACTTTA  31 200
            ||||||||||||||||||||||||||||||||||||||||||||||||||||||||||||
2   31 141  TTGTCCCAGCCAATCAGCCTCGCCCACCTTCTCCCACCCCCACTGAAATCAGCTACTTTA  31 200

1   31 201  ATCTAACAGGAGGAGACGACTGATAAAAAAAAATAATAAAGCATCACTTACTTAAAATCA  31 260
            ||||||||||||||||||||||||||||||||||||||||||||||||||||||||||||
2   31 201  ATCTAACAGGAGGAGACGACTGATAAAAAAAAATAATAAAGCATCACTTACTTAAAATCA  31 260

1   31 261  GTTAGCAAATTTCTGTCCAGTTTATTCAGCAGCACCTCCTTGCCCTCCTCCCAGCTCTGG  31 320
            ||||||||||||||||||||||||||||||||||||||||||||||||||||||||||||
2   31 261  GTTAGCAAATTTCTGTCCAGTTTATTCAGCAGCACCTCCTTGCCCTCCTCCCAGCTCTGG  31 320

1   31 321  TATTGCAGCTTCCTCCTGGCTGCAAACTTTCTCCACAATCTAAATGGAATGTCAGTTTCC  31 380
            ||||||||||||||||||||||||||||||||||||||||||||||||||||||||||||
2   31 321  TATTGCAGCTTCCTCCTGGCTGCAAACTTTCTCCACAATCTAAATGGAATGTCAGTTTCC  31 380

1   31 381  TCCTGTTCCTGTCCATCCGCACCCACTATCTTCATGTTGTTGCAGATGAAGCGCGCAAGA  31 440
            ||||||||||||||||||||||||||||||||||||||||||||||||||||||||||||
2   31 381  TCCTGTTCCTGTCCATCCGCACCCACTATCTTCATGTTGTTGCAGATGAAGCGCGCAAGA  31 440

1   31 441  CCGTCTGAAGATACCTTCAACCCCGTGTATCCATATGACACGGAAACCGGTCCTCCAACT  31 500
            ||||||||||||||||||||||||||||||||||||||||||||||||||||||||||||
2   31 441  CCGTCTGAAGATACCTTCAACCCCGTGTATCCATATGACACGGAAACCGGTCCTCCAACT  31 500

1   31 501  GTGCCTTTTCTTACTCCTCCCTTTGTATCCCCCAATGGGTTTCAAGAGAGTCCCCCTGGG  31 560
            ||||||||||||||||||||||||||||||||||||||||||||||||||||||||||||
2   31 501  GTGCCTTTTCTTACTCCTCCCTTTGTATCCCCCAATGGGTTTCAAGAGAGTCCCCCTGGG  31 560

1   31 561  GTACTCTCTTTGCGCCTATCCGAACCTCTAGTTACCTCCAATGGCATGCTTGCGCTCAAA  31 620
            ||||||||||||||||||||||||||||||||||||||||||||||||||||||||||||
2   31 561  GTACTCTCTTTGCGCCTATCCGAACCTCTAGTTACCTCCAATGGCATGCTTGCGCTCAAA  31 620

1   31 621  ATGGGCAACGGCCTCTCTCTGGACGAGGCCGGCAACCTTACCTCCCAAAATGTAACCACT  31 680
            ||||||||||||||||||||||||||||||||||||||||||||||||||||||||||||
2   31 621  ATGGGCAACGGCCTCTCTCTGGACGAGGCCGGCAACCTTACCTCCCAAAATGTAACCACT  31 680

1   31 681  GTGAGCCCACCTCTCAAAAAAACCAAGTCAAACATAAACCTGGAAATATCTGCACCCCTC  31 740
            ||||||||||||||||||||||||||||||||||||||||||||||||||||||||||||
2   31 681  GTGAGCCCACCTCTCAAAAAAACCAAGTCAAACATAAACCTGGAAATATCTGCACCCCTC  31 740
```

```
1   31 741  ACAGTTACCTCAGAAGCCCTAACTGTGGCTGCCGCCGCACCTCTAATGGTCGCGGGCAAC  31 800
            ||||||||||||||||||||||||||||||||||||||||||||||||||||||||||||
2   31 741  ACAGTTACCTCAGAAGCCCTAACTGTGGCTGCCGCCGCACCTCTAATGGTCGCGGGCAAC  31 800

1   31 801  ACACTCACCATGCAATCACAGGCCCCGCTAACCGTGCACGACTCCAAACTTAGCATTGCC  31 860
            ||||||||||||||||||||||||||||||||||||||||||||||||||||||||||||
2   31 801  ACACTCACCATGCAATCACAGGCCCCGCTAACCGTGCACGACTCCAAACTTAGCATTGCC  31 860

1   31 861  ACCCAAGGACCCCTCACAGTGTCAGAAGGAAAGCTAGCCCTGCAAACATCAGGCCCCCTC  31 920
            ||||||||||||||||||||||||||||||||||||||||||||||||||||||||||||
2   31 861  ACCCAAGGACCCCTCACAGTGTCAGAAGGAAAGCTAGCCCTGCAAACATCAGGCCCCCTC  31 920

1   31 921  ACCACCACCGATAGCAGTACCCTTACTATCACTGCCTCACCCCCTCTAACTACTGCCACT  31 980
            ||||||||||||||||||||||||||||||||||||||||||||||||||||||||||||
2   31 921  ACCACCACCGATAGCAGTACCCTTACTATCACTGCCTCACCCCCTCTAACTACTGCCACT  31 980

1   31 981  GGTAGCTTGGGCATTGACTTGAAAGAGCCCATTTATACACAAAATGGAAAACTAGGACTA  32 040
            ||||||||||||||||||||||||||||||||||||||||||||||||||||||||||||
2   31 981  GGTAGCTTGGGCATTGACTTGAAAGAGCCCATTTATACACAAAATGGAAAACTAGGACTA  32 040

1   32 041  AAGTACGGGGCTCCTTTGCATGTAACAGACGACCTAAACACTTTGACCGTAGCAACTGGT  32 100
            ||||||||||||||||||||||||||||||||||||||||||||||||||||||||||||
2   32 041  AAGTACGGGGCTCCTTTGCATGTAACAGACGACCTAAACACTTTGACCGTAGCAACTGGT  32 100

1   32 101  CCAGGTGTGACTATTAATAATACTTCCTTGCAAACTAAAGTTACTGGAGCCTTGGGTTTT  32 160
            ||||||||||||||||||||||||||||||||||||||||||||||||||||||||||||
2   32 101  CCAGGTGTGACTATTAATAATACTTCCTTGCAAACTAAAGTTACTGGAGCCTTGGGTTTT  32 160

1   32 161  GATTCACAAGGCAATATGCAACTTAATGTAGCAGGAGGACTAAGGATTGATTCTCAAAAC  32 220
            ||||||||||||||||||||||||||||||||||||||||||||||||||||||||||||
2   32 161  GATTCACAAGGCAATATGCAACTTAATGTAGCAGGAGGACTAAGGATTGATTCTCAAAAC  32 220

1   32 221  AGACGCCTTATACTTGATGTTAGTTATCCGTTTGATGCTCAAAACCAACTAAATCTAAGA  32 280
            ||||||||||||||||||||||||||||||||||||||||||||||||||||||||||||
2   32 221  AGACGCCTTATACTTGATGTTAGTTATCCGTTTGATGCTCAAAACCAACTAAATCTAAGA  32 280

1   32 281  CTAGGACAGGGCCCTCTTTTTATAAACTCAGCCCACAACTTGGATATTAACTACAACAAA  32 340
            ||||||||||||||||||||||||||||||||||||||||||||||||||||||||||||
2   32 281  CTAGGACAGGGCCCTCTTTTTATAAACTCAGCCCACAACTTGGATATTAACTACAACAAA  32 340
```

```
1   32 341  GGCCTTTACTTGTTTACAGCTTCAAACAATTCCAAAAAGCTTGAGGTTAACCTAAGCACT  32 400
            ||||||||||||||||||||||||||||||||||||||||||||||||||||||||||||
2   32 341  GGCCTTTACTTGTTTACAGCTTCAAACAATTCCAAAAAGCTTGAGGTTAACCTAAGCACT  32 400

1   32 401  GCCAAGGGGTTGATGTTTGACGCTACAGCCATAGCCATTAATGCAGGAGATGGGCTTGAA  32 460
            ||||||||||||||||||||||||||||||||||||||||||||||||||||||||||||
2   32 401  GCCAAGGGGTTGATGTTTGACGCTACAGCCATAGCCATTAATGCAGGAGATGGGCTTGAA  32 460

1   32 461  TTTGGTTCACCTAATGCACCAAACACAAATCCCCTCAAAACAAAAATTGGCCATGGCCTA  32 520
            ||||||||||||||||||||||||||||||||||||||||||||||||||||||||||||
2   32 461  TTTGGTTCACCTAATGCACCAAACACAAATCCCCTCAAAACAAAAATTGGCCATGGCCTA  32 520

1   32 521  GAATTTGATTCAAACAAGGCTATGGTTCCTAAACTAGGAACTGGCCTTAGTTTTGACAGC  32 580
            ||||||||||||||||||||||||||||||||||||||||||||||||||||||||||||
2   32 521  GAATTTGATTCAAACAAGGCTATGGTTCCTAAACTAGGAACTGGCCTTAGTTTTGACAGC  32 580

1   32 581  ACAGGTGCCATTACAGTAGGAAACAAAAATAATGATAAGCTAACCCTATGGACAGGTCCA  32 640
            ||||||||||||||||||||||||||||||||||||||||||||||||||||||||||||
2   32 581  ACAGGTGCCATTACAGTAGGAAACAAAAATAATGATAAGCTAACCCTATGGACAGGTCCA  32 640

1   32 641  AAACCAGAAGCCAACTGCATAATTGAATACGGGAAACAAAACCCAGATAGCAAACTAACT  32 700
            ||||||||||||||||||||||||||||||||||||||||||||||||||||||||||||
2   32 641  AAACCAGAAGCCAACTGCATAATTGAATACGGGAAACAAAACCCAGATAGCAAACTAACT  32 700

1   32 701  TTAATCCTTGTAAAAAATGGAGGAATTGTTAATGGATATGTAACGCTAATGGGAGCCTCA  32 760
            ||||||||||||||||||||||||||||||||||||||||||||||||||||||||||||
2   32 701  TTAATCCTTGTAAAAAATGGAGGAATTGTTAATGGATATGTAACGCTAATGGGAGCCTCA  32 760

1   32 761  GACTACGTTAACACCTTATTTAAAAACAAAAATGTCTCCATTAATGTAGAACTATACTTT  32 820
            ||||||||||||||||||||||||||||||||||||||||||||||||||||||||||||
2   32 761  GACTACGTTAACACCTTATTTAAAAACAAAAATGTCTCCATTAATGTAGAACTATACTTT  32 820

1   32 821  GATGCCACTGGTCATATATTACCAGACTCATCTTCTCTTAAAACAGATCTAGAACTAAAA  32 880
            ||||||||||||||||||||||||||||||||||||||||||||||||||||||||||||
2   32 821  GATGCCACTGGTCATATATTACCAGACTCATCTTCTCTTAAAACAGATCTAGAACTAAAA  32 880

1   32 881  TACAAGCAAACCGCTGACTTTAGTGCAAGAGGTTTTATGCCAAGTACTACAGCGTATCCA  32 940
            ||||||||||||||||||||||||||||||||||||||||||||||||||||||||||||
2   32 881  TACAAGCAAACCGCTGACTTTAGTGCAAGAGGTTTTATGCCAAGTACTACAGCGTATCCA  32 940
```

```
1   32 941  TTTGTCCTTCCTAATGCGGGAACACATAATGAAAATTATATTTTTGGTCAATGCTACTAC  33 000
            ||||||||||||||||||||||||||||||||||||||||||||||||||||||||||||
2   32 941  TTTGTCCTTCCTAATGCGGGAACACATAATGAAAATTATATTTTTGGTCAATGCTACTAC  33 000

1   33 001  AAAGCAAGCGATGGTGCCCTTTTTCCGTTGGAAGTTACTGTTATGCTTAATAAACGCCTG  33 060
            ||||||||||||||||||||||||||||||||||||||||||||||||||||||||||||
2   33 001  AAAGCAAGCGATGGTGCCCTTTTTCCGTTGGAAGTTACTGTTATGCTTAATAAACGCCTG  33 060

1   33 061  CCAGATAGTCGCACATCCTATGTTATGACTTTTTTATGGTCCTTGAATGCTGGTCTAGCT  33 120
            ||||||||||||||||||||||||||||||||||||||||||||||||||||||||||||
2   33 061  CCAGATAGTCGCACATCCTATGTTATGACTTTTTTATGGTCCTTGAATGCTGGTCTAGCT  33 120

1   33 121  CCAGAAACTACTCAGGCAACCCTCATAACCTCCCCATTTACCTTTTCCTATATTAGAGAA  33 180
            ||||||||||||||||||||||||||||||||||||||||||||||||||||||||||||
2   33 121  CCAGAAACTACTCAGGCAACCCTCATAACCTCCCCATTTACCTTTTCCTATATTAGAGAA  33 180

1   33 181  GATGACTAATAAACTCTAAAGAATCGTTTGTGTTATGTTTCAACGTGTTTATTTTTCAAT  33 240
            ||||||||||||||||||||||||||||||||||||||||||||||||||||||||||||
2   33 181  GATGACTAATAAACTCTAAAGAATCGTTTGTGTTATGTTTCAACGTGTTTATTTTTCAAT  33 240

1   33 241  TGCAGAAAATTTCAAGTCATTTTTCATTCAGTAGTATAGCCCCACCACCACATAGCTTAT  33 300
            ||||||||||||||||||||||||||||||||||||||||||||||||||||||||||||
2   33 241  TGCAGAAAATTTCAAGTCATTTTTCATTCAGTAGTATAGCCCCACCACCACATAGCTTAT  33 300

1   33 301  ACAGATCACCGTACCTTAATCAAACTCACAGAACCCTAGTATTCAACCTGCCACCTCCCT  33 360
            ||||||||||||||||||||||||||||||||||||||||||||||||||||||||||||
2   33 301  ACAGATCACCGTACCTTAATCAAACTCACAGAACCCTAGTATTCAACCTGCCACCTCCCT  33 360

1   33 361  CCCAACACACAGAGTACACAGTCCTTTCTCCCCGGCTGGCCTTAAAAAGCATCATATCAT  33 420
            ||||||||||||||||||||||||||||||||||||||||||||||||||||||||||||
2   33 361  CCCAACACACAGAGTACACAGTCCTTTCTCCCCGGCTGGCCTTAAAAAGCATCATATCAT  33 420

1   33 421  GGGTAACAGACATATTCTTAGGTGTTATATTCCACACGGTTTCCTGTCGAGCCAAACGCT  33 480
            ||||||||||||||||||||||||||||||||||||||||||||||||||||||||||||
2   33 421  GGGTAACAGACATATTCTTAGGTGTTATATTCCACACGGTTTCCTGTCGAGCCAAACGCT  33 480

1   33 481  CATCAGTGATATTAATAAACTCCCCGGGCAGCTCACTTAAGTTCATGTCGCTGTCCAGCT  33 540
            ||||||||||||||||||||||||||||||||||||||||||||||||||||||||||||
2   33 481  CATCAGTGATATTAATAAACTCCCCGGGCAGCTCACTTAAGTTCATGTCGCTGTCCAGCT  33 540
```

```
1   33 541  GCTGAGCCACAGGCTGCTGTCCAACTTGCGGTTGCTTAACGGGCGGCGAAGGAGAAGTCC  33 600
            ||||||||||||||||||||||||||||||||||||||||||||||||||||||||||||
2   33 541  GCTGAGCCACAGGCTGCTGTCCAACTTGCGGTTGCTTAACGGGCGGCGAAGGAGAAGTCC  33 600

1   33 601  ACGCCTACATGGGGGTAGAGTCATAATCGTGCATCAGGATAGGGCGGTGGTGCTGCAGCA  33 660
            ||||||||||||||||||||||||||||||||||||||||||||||||||||||||||||
2   33 601  ACGCCTACATGGGGGTAGAGTCATAATCGTGCATCAGGATAGGGCGGTGGTGCTGCAGCA  33 660

1   33 661  GCGCGCGAATAAACTGCTGCCGCCGCCGCTCCGTCCTGCAGGAATACAACATGGCAGTGG  33 720
            ||||||||||||||||||||||||||||||||||||||||||||||||||||||||||||
2   33 661  GCGCGCGAATAAACTGCTGCCGCCGCCGCTCCGTCCTGCAGGAATACAACATGGCAGTGG  33 720

1   33 721  TCTCCTCAGCGATGATTCGCACCGCCCGCAGCATAAGGCGCCTTGTCCTCCGGGCACAGC  33 780
            ||||||||||||||||||||||||||||||||||||||||||||||||||||||||||||
2   33 721  TCTCCTCAGCGATGATTCGCACCGCCCGCAGCATAAGGCGCCTTGTCCTCCGGGCACAGC  33 780

1   33 781  AGCGCACCCTGATCTCACTTAAATCAGCACAGTAACTGCAGCACAGCACCACAATATTGT  33 840
            ||||||||||||||||||||||||||||||||||||||||||||||||||||||||||||
2   33 781  AGCGCACCCTGATCTCACTTAAATCAGCACAGTAACTGCAGCACAGCACCACAATATTGT  33 840

1   33 841  TCAAAATCCCACAGTGCAAGGCGCTGTATCCAAAGCTCATGGCGGGGACCACAGAACCCA  33 900
            ||||||||||||||||||||||||||||||||||||||||||||||||||||||||||||
2   33 841  TCAAAATCCCACAGTGCAAGGCGCTGTATCCAAAGCTCATGGCGGGGACCACAGAACCCA  33 900

1   33 901  CGTGGCCATCATACCACAAGCGCAGGTAGATTAAGTGGCGACCCCTCATAAACACGCTGG  33 960
            ||||||||||||||||||||||||||||||||||||||||||||||||||||||||||||
2   33 901  CGTGGCCATCATACCACAAGCGCAGGTAGATTAAGTGGCGACCCCTCATAAACACGCTGG  33 960

1   33 961  ACATAAACATTACCTCTTTTGGCATGTTGTAATTCACCACCTCCCGGTACCATATAAACC  34 020
            ||||||||||||||||||||||||||||||||||||||||||||||||||||||||||||
2   33 961  ACATAAACATTACCTCTTTTGGCATGTTGTAATTCACCACCTCCCGGTACCATATAAACC  34 020

1   34 021  TCTGATTAAACATGGCGCCATCCACCACCATCCTAAACCAGCTGGCCAAAACCTGCCCGC  34 080
            ||||||||||||||||||||||||||||||||||||||||||||||||||||||||||||
2   34 021  TCTGATTAAACATGGCGCCATCCACCACCATCCTAAACCAGCTGGCCAAAACCTGCCCGC  34 080

1   34 081  CGGCTATACACTGCAGGGAACCGGGACTGGAACAATGACAGTGGAGAGCCCAGGACTCGT  34 140
            ||||||||||||||||||||||||||||||||||||||||||||||||||||||||||||
2   34 081  CGGCTATACACTGCAGGGAACCGGGACTGGAACAATGACAGTGGAGAGCCCAGGACTCGT  34 140
```

```
1   34 141  AACCATGGATCATCATGCTCGTCATGATATCAATGTTGGCACAACACAGGCACACGTGCA  34 200
            ||||||||||||||||||||||||||||||||||||||||||||||||||||||||||||
2   34 141  AACCATGGATCATCATGCTCGTCATGATATCAATGTTGGCACAACACAGGCACACGTGCA  34 200

1   34 201  TACACTTCCTCAGGATTACAAGCTCCTCCCGCGTTAGAACCATATCCCAGGGAACAACCC  34 260
            ||||||||||||||||||||||||||||||||||||||||||||||||||||||||||||
2   34 201  TACACTTCCTCAGGATTACAAGCTCCTCCCGCGTTAGAACCATATCCCAGGGAACAACCC  34 260

1   34 261  ATTCCTGAATCAGCGTAAATCCCACACTGCAGGGAAGACCTCGCACGTAACTCACGTTGT  34 320
            ||||||||||||||||||||||||||||||||||||||||||||||||||||||||||||
2   34 261  ATTCCTGAATCAGCGTAAATCCCACACTGCAGGGAAGACCTCGCACGTAACTCACGTTGT  34 320

1   34 321  GCATTGTCAAAGTGTTACATTCGGGCAGCAGCGGATGATCCTCCAGTATGGTAGCGCGGG  34 380
            ||||||||||||||||||||||||||||||||||||||||||||||||||||||||||||
2   34 321  GCATTGTCAAAGTGTTACATTCGGGCAGCAGCGGATGATCCTCCAGTATGGTAGCGCGGG  34 380

1   34 381  TTTCTGTCTCAAAAGGAGGTAGACGATCCCTACTGTACGGAGTGCGCCGAGACAACCGAG  34 440
            ||||||||||||||||||||||||||||||||||||||||||||||||||||||||||||
2   34 381  TTTCTGTCTCAAAAGGAGGTAGACGATCCCTACTGTACGGAGTGCGCCGAGACAACCGAG  34 440

1   34 441  ATCGTGTTGGTCGTAGTGTCATGCCAAATGGAACGCCGGACGTAGTCATATTTCCTGAAG  34 500
            ||||||||||||||||||||||||||||||||||||||||||||||||||||||||||||
2   34 441  ATCGTGTTGGTCGTAGTGTCATGCCAAATGGAACGCCGGACGTAGTCATATTTCCTGAAG  34 500

1   34 501  CAAAACCAGGTGCGGGCGTGACAAACAGATCTGCGTCTCCGGTCTCGCCGCTTAGATCGC  34 560
            ||||||||||||||||||||||||||||||||||||||||||||||||||||||||||||
2   34 501  CAAAACCAGGTGCGGGCGTGACAAACAGATCTGCGTCTCCGGTCTCGCCGCTTAGATCGC  34 560

1   34 561  TCTGTGTAGTAGTTGTAGTATATCCACTCTCTCAAAGCATCCAGGCGCCCCCTGGCTTCG  34 620
            ||||||||||||||||||||||||||||||||||||||||||||||||||||||||||||
2   34 561  TCTGTGTAGTAGTTGTAGTATATCCACTCTCTCAAAGCATCCAGGCGCCCCCTGGCTTCG  34 620

1   34 621  GGTTCTATGTAAACTCCTTCATGCGCCGCTGCCCTGATAACATCCACCACCGCAGAATAA  34 680
            ||||||||||||||||||||||||||||||||||||||||||||||||||||||||||||
2   34 621  GGTTCTATGTAAACTCCTTCATGCGCCGCTGCCCTGATAACATCCACCACCGCAGAATAA  34 680

1   34 681  GCCACACCCAGCCAACCTACACATTCGTTCTGCGAGTCACACACGGGAGGAGCGGGAAGA  34 740
            ||||||||||||||||||||||||||||||||||||||||||||||||||||||||||||
2   34 681  GCCACACCCAGCCAACCTACACATTCGTTCTGCGAGTCACACACGGGAGGAGCGGGAAGA  34 740
```

```
1   34 741  GCTGGAAGAACCATGTTTTTTTTTTTATTCCAAAAGATTATCCAAAACCTCAAAATGAAG  34 800
            ||||||||||||||||||||||||||||||||||||||||||||||||||||||||||||
2   34 741  GCTGGAAGAACCATGTTTTTTTTTTTATTCCAAAAGATTATCCAAAACCTCAAAATGAAG  34 800

1   34 801  ATCTATTAAGTGAACGCGCTCCCCTCCGGTGGCGTGGTCAAACTCTACAGCCAAAGAACA  34 860
            ||||||||||||||||||||||||||||||||||||||||||||||||||||||||||||
2   34 801  ATCTATTAAGTGAACGCGCTCCCCTCCGGTGGCGTGGTCAAACTCTACAGCCAAAGAACA  34 860

1   34 861  GATAATGGCATTTGTAAGATGTTGCACAATGGCTTCCAAAAGGCAAACGGCCCTCACGTC  34 920
            ||||||||||||||||||||||||||||||||||||||||||||||||||||||||||||
2   34 861  GATAATGGCATTTGTAAGATGTTGCACAATGGCTTCCAAAAGGCAAACGGCCCTCACGTC  34 920

1   34 921  CAAGTGGACGTAAAGGCTAAACCCTTCAGGGTGAATCTCCTCTATAAACATTCCAGCACC  34 980
            ||||||||||||||||||||||||||||||||||||||||||||||||||||||||||||
2   34 921  CAAGTGGACGTAAAGGCTAAACCCTTCAGGGTGAATCTCCTCTATAAACATTCCAGCACC  34 980

1   34 981  TTCAACCATGCCCAAATAATTCTCATCTCGCCACCTTCTCAATATATCTCTAAGCAAATC  35 040
            ||||||||||||||||||||||||||||||||||||||||||||||||||||||||||||
2   34 981  TTCAACCATGCCCAAATAATTCTCATCTCGCCACCTTCTCAATATATCTCTAAGCAAATC  35 040

1   35 041  CCGAATATTAAGTCCGGCCATTGTAAAAATCTGCTCCAGAGCGCCCTCCACCTTCAGCCT  35 100
            ||||||||||||||||||||||||||||||||||||||||||||||||||||||||||||
2   35 041  CCGAATATTAAGTCCGGCCATTGTAAAAATCTGCTCCAGAGCGCCCTCCACCTTCAGCCT  35 100

1   35 101  CAAGCAGCGAATCATGATTGCAAAAATTCAGGTTCCTCACAGACCTGTATAAGATTCAAA  35 160
            ||||||||||||||||||||||||||||||||||||||||||||||||||||||||||||
2   35 101  CAAGCAGCGAATCATGATTGCAAAAATTCAGGTTCCTCACAGACCTGTATAAGATTCAAA  35 160

1   35 161  AGCGGAACATTAACAAAAATACCGCGATCCCGTAGGTCCCTTCGCAGGGCCAGCTGAACA  35 220
            ||||||||||||||||||||||||||||||||||||||||||||||||||||||||||||
2   35 161  AGCGGAACATTAACAAAAATACCGCGATCCCGTAGGTCCCTTCGCAGGGCCAGCTGAACA  35 220

1   35 221  TAATCGTGCAGGTCTGCACGGACCAGCGCGGCCACTTCCCCGCCAGGAACCATGACAAAA  35 280
            ||||||||||||||||||||||||||||||||||||||||||||||||||||||||||||
2   35 221  TAATCGTGCAGGTCTGCACGGACCAGCGCGGCCACTTCCCCGCCAGGAACCATGACAAAA  35 280

1   35 281  GAACCCACACTGATTATGACACGCATACTCGGAGCTATGCTAACCAGCGTAGCCCCGATG  35 340
            ||||||||||||||||||||||||||||||||||||||||||||||||||||||||||||
2   35 281  GAACCCACACTGATTATGACACGCATACTCGGAGCTATGCTAACCAGCGTAGCCCCGATG  35 340
```

```
1   35 341  TAAGCTTGTTGCATGGGCGGCGATATAAAATGCAAGGTGCTGCTCAAAAAATCAGGCAAA  35 400
            ||||||||||||||||||||||||||||||||||||||||||||||||||||||||||||
2   35 341  TAAGCTTGTTGCATGGGCGGCGATATAAAATGCAAGGTGCTGCTCAAAAAATCAGGCAAA  35 400

1   35 401  GCCTCGCGCAAAAAAGAAAGCACATCGTAGTCATGCTCATGCAGATAAAGGCAGGTAAGC  35 460
            ||||||||||||||||||||||||||||||||||||||||||||||||||||||||||||
2   35 401  GCCTCGCGCAAAAAAGAAAGCACATCGTAGTCATGCTCATGCAGATAAAGGCAGGTAAGC  35 460

1   35 461  TCCGGAACCACCACAGAAAAAGACACCATTTTTCTCTCAAACATGTCTGCGGGTTTCTGC  35 520
            ||||||||||||||||||||||||||||||||||||||||||||||||||||||||||||
2   35 461  TCCGGAACCACCACAGAAAAAGACACCATTTTTCTCTCAAACATGTCTGCGGGTTTCTGC  35 520

1   35 521  ATAAACACAAAATAAAATAACAAAAAAACATTTAAACATTAGAAGCCTGTCTTACAACAG  35 580
            ||||||||||||||||||||||||||||||||||||||||||||||||||||||||||||
2   35 521  ATAAACACAAAATAAAATAACAAAAAAACATTTAAACATTAGAAGCCTGTCTTACAACAG  35 580

1   35 581  GAAAAACAACCCTTATAAGCATAAGACGGACTACGGCCATGCCGGCGTGACCGTAAAAAA  35 640
            ||||||||||||||||||||||||||||||||||||||||||||||||||||||||||||
2   35 581  GAAAAACAACCCTTATAAGCATAAGACGGACTACGGCCATGCCGGCGTGACCGTAAAAAA  35 640

1   35 641  ACTGGTCACCGTGATTAAAAAGCACCACCGACAGCTCCTCGGTCATGTCCGGAGTCATAA  35 700
            ||||||||||||||||||||||||||||||||||||||||||||||||||||||||||||
2   35 641  ACTGGTCACCGTGATTAAAAAGCACCACCGACAGCTCCTCGGTCATGTCCGGAGTCATAA  35 700

1   35 701  TGTAAGACTCGGTAAACACATCAGGTTGATTCACATCGGTCAGTGCTAAAAAGCGACCGA  35 760
            ||||||||||||||||||||||||||||||||||||||||||||||||||||||||||||
2   35 701  TGTAAGACTCGGTAAACACATCAGGTTGATTCACATCGGTCAGTGCTAAAAAGCGACCGA  35 760

1   35 761  AATAGCCCGGGGGAATACATACCCGCAGGCGTAGAGACAACATTACAGCCCCCATAGGAG  35 820
            ||||||||||||||||||||||||||||||||||||||||||||||||||||||||||||
2   35 761  AATAGCCCGGGGGAATACATACCCGCAGGCGTAGAGACAACATTACAGCCCCCATAGGAG  35 820

1   35 821  GTATAACAAAATTAATAGGAGAGAAAAACACATAAACACCTGAAAAACCCTCCTGCCTAG  35 880
            ||||||||||||||||||||||||||||||||||||||||||||||||||||||||||||
2   35 821  GTATAACAAAATTAATAGGAGAGAAAAACACATAAACACCTGAAAAACCCTCCTGCCTAG  35 880

1   35 881  GCAAAATAGCACCCTCCCGCTCCAGAACAACATACAGCGCTTCCACAGCGGCAGCCATAA  35 940
            ||||||||||||||||||||||||||||||||||||||||||||||||||||||||||||
2   35 881  GCAAAATAGCACCCTCCCGCTCCAGAACAACATACAGCGCTTCCACAGCGGCAGCCATAA  35 940
```

```
1   35 941  CAGTCAGCCTTACCAGTAAAAAAGAAAACCTATTAAAAAAACACCACTCGACACGGCACC  36 000
            ||||||||||||||||||||||||||||||||||||||||||||||||||||||||||||
2   35 941  CAGTCAGCCTTACCAGTAAAAAAGAAAACCTATTAAAAAAACACCACTCGACACGGCACC  36 000

1   36 001  AGCTCAATCAGTCACAGTGTAAAAAAGGGCCAAGTGCAGAGCGAGTATATATAGGACTAA  36 060
            ||||||||||||||||||||||||||||||||||||||||||||||||||||||||||||
2   36 001  AGCTCAATCAGTCACAGTGTAAAAAAGGGCCAAGTGCAGAGCGAGTATATATAGGACTAA  36 060

1   36 061  AAAATGACGTAACGGTTAAAGTCCACAAAAAACACCCAGAAAACCGCACGCGAACCTACG  36 120
            ||||||||||||||||||||||||||||||||||||||||||||||||||||||||||||
2   36 061  AAAATGACGTAACGGTTAAAGTCCACAAAAAACACCCAGAAAACCGCACGCGAACCTACG  36 120

1   36 121  CCCAGAAACGAAAGCCAAAAAACCCACAACTTCCTCAAATCGTCACTTCCGTTTTCCCAC  36 180
            ||||||||||||||||||||||||||||||||||||||||||||||||||||||||||||
2   36 121  CCCAGAAACGAAAGCCAAAAAACCCACAACTTCCTCAAATCGTCACTTCCGTTTTCCCAC  36 180

1   36 181  GTTACGTCACTTCCCATTTTAAGAAAACTACAATTCCCAACACATACAAGTTACTCCGCC  36 240
            ||||||||||||||||||||||||||||||||||||||||||||||||||||||||||||
2   36 181  GTTACGTCACTTCCCATTTTAAGAAAACTACAATTCCCAACACATACAAGTTACTCCGCC  36 240

1   36 241  CTAAAACCTACGTCACCCGCCCCGTTCCCACGCCCCGCGCCACGTCACAAACTCCACCCC  36 300
            ||||||||||||||||||||||||||||||||||||||||||||||||||||||||||||
2   36 241  CTAAAACCTACGTCACCCGCCCCGTTCCCACGCCCCGCGCCACGTCACAAACTCCACCCC  36 300

1   36 301  CTCATTATCATATTGGCTTCAATCCAAAATAAGGTATATTATTGATGATG  36 350
            ||||||||||||||||||||||||||||||||||||||||||||||||||
2   36 301  CTCATTATCATATTGGCTTCAATCCAAAATAAGGTATATTATTGATGATG  36 350
```

**Sequence Logo:** 50% GC base composition

**Consensus Threshold:** >50%

**Compare to:** the consensus

Bases that match the reference are marked with yellow highlighting.

**Created:** 22. tammik. 2021

**Last Modified:** 22. tammik. 2021
