# Supplementary material for: Characterization of a novel OX40 ligand and CD40 ligand-expressing oncolytic adenovirus used in the PeptiCRAd cancer vaccine platform
Source: Mol Ther Oncolytics. 2021 Feb 10;20:459–69. doi: 10.1016/j.omto.2021.02.006 (PMC7917457; doi:10.1016/j.omto.2021.02.006)
Supplement: Document S2. Article plus supplemental information [file mmc3.pdf]

Molecular Therapy
Oncolytics
Original Article

Characterization of a novel OX40 ligand and CD40 ligand-expressing oncolytic adenovirus used in the PeptiCRAd cancer vaccine platform

Erkko Ylösmäki,[1,2,8] Leena Ylösmäki,[3,8] Manlio Fusciello,[1,2] Beatriz Martins,[1,2] Petra Ahokas,[3] Hanne Cojoc,[3] Arttu Uoti,[1,2] Sara Feola,[1,2] Anna Kreutzman,[1,2] Tuuli Ranki,[3] Julia Karbach,[4] Tapani Viitala,[5] Petri Priha,[3] Elke Jäger,[4] Sari Pesonen,[3] and Vincenzo Cerullo[1,2,6,7]

[1]Laboratory of Immunovirotherapy, Drug Research Program, Faculty of Pharmacy, University of Helsinki, Helsinki, Finland; [2]TRIMM, Translational Immunology Research Program, University of Helsinki, Finland; [3]Valo Therapeutics Oy, Helsinki, Finland; [4]Department of Oncology and Hematology, Krankenhaus Nordwest, Frankfurt am Main, Germany; [5]Pharmaceutical Biophysics Research Group, Drug Research Program, Faculty of Pharmacy, University of Helsinki, Helsinki, Finland; [6]iCAN Digital Precision Cancer Medicine Flagship, University of Helsinki, Helsinki, Finland; [7]Department of Molecular Medicine and Medical Biotechnology and CEINGE, Naples University, 24 Federico II, 80131 Naples, Italy

Oncolytic viruses (OVs) have been shown to induce anti-cancer immunity and enhance cancer immunotherapies, such as immune checkpoint inhibitor therapies. OV therapies can be further improved by arming OVs with immunostimulatory molecules, including various cytokines or chemokines. Here, we have developed a novel adenovirus encoding two immunostimulatory molecules: cluster of differentiation 40 ligand (CD40L) and tumor necrosis factor receptor superfamily member 4 ligand (OX40L). This novel virus, designated VALO-D102, is designed to activate both innate and adaptive immune responses against tumors. CD40L affects the innate side by licensing antigen-presenting cells to drive $CD8^+$ T cell responses, and OX40L increases clonal expansion and survival of $CD8^+$ T cells and formation of a larger pool of memory T cells. VALO-D102 and its murine surrogate VALO-mD901, expressing murine OX40L and CD40L, were used in our previously developed PeptiCRAd cancer vaccine platform. Intratumoral administration of PeptiCRAd significantly increased tumor-specific T cell responses, reduced tumor growth, and induced systemic anti-cancer immunity in two mouse models of melanoma. In addition, PeptiCRAd therapy, in combination with anti-PD-1 immune checkpoint inhibitor therapy, significantly improved tumor growth control as compared to either monotherapy alone.

INTRODUCTION

Recently, cancer immunotherapies, including antibodies targeting immune checkpoint molecules, such as PD-1, PD-L1 and CTLA-4, have emerged as an unprecedented breakthrough for the treatment of cancer that can induce long-term tumor regression. Unfortunately, the use of immune checkpoint inhibitor (ICI) antibodies can create durable responses in only a small minority of cancer patients, the response rate being 10%–25% in a majority of cancers.[1] The common feature among patients responding to ICI therapy is that they have an existing antitumor immune response with immune cell infiltration in the tumor tissue prior to ICI therapy.[2,3] These immune cell-infiltrated tumors are called "hot" tumors. The remaining 75%–90% of patients are not responding due to a lack of anti-tumor immune responses or other immune-suppressive aspects of the tumor microenvironment (TME). These tumors that have not been recognized by the immune system or have not provoked a spontaneous immune response are collectively called "cold" tumors.

Oncolytic viruses (OVs) are naturally occurring or specifically designed viruses that infect and kill cancer cells, leaving healthy cells unharmed.[4] OVs can induce tumor cell killing through direct lysis of infected tumor cells or through induction of a strong antiviral immune response, leading to the elimination of OV-infected cells. In addition, virus-mediated lysis of tumor cells can release tumor-associated antigens and neoantigens that eventually initiate a tumor-specific T cell response against the released tumor antigens.[5] Importantly, OVs have an intrinsic capacity to turn immunologically cold tumors into hot ones. This feature has made them highly promising candidates for combination with ICI therapies for the treatment of nonresponsive or cold tumors.[6,7] A major disadvantage of most OVs currently used in the clinic is that, although they induce a strong antiviral immune response, the OV-induced anti-tumor immune response remains modest and thus reduces the therapeutic potential of these viruses.[8,9] In an attempt to overcome this disadvantage and to increase the anti-tumor immune response induced by OVs, we developed a conditionally replicating oncolytic adenovirus (OAd) armed with

Received 24 November 2020; accepted 4 February 2021;
https://doi.org/10.1016/j.omto.2021.02.006.

[8]These authors contributed equally

Correspondence: Vincenzo Cerullo, Laboratory of Immunovirotherapy, Drug Research Program, Faculty of Pharmacy, University of Helsinki, Viikinkaari 5E, 00790 Helsinki, Finland.
E-mail: vincenzo.cerullo@helsinki.fi

This is an open access article under the CC BY license (http://creativecommons.org/licenses/by/4.0/).

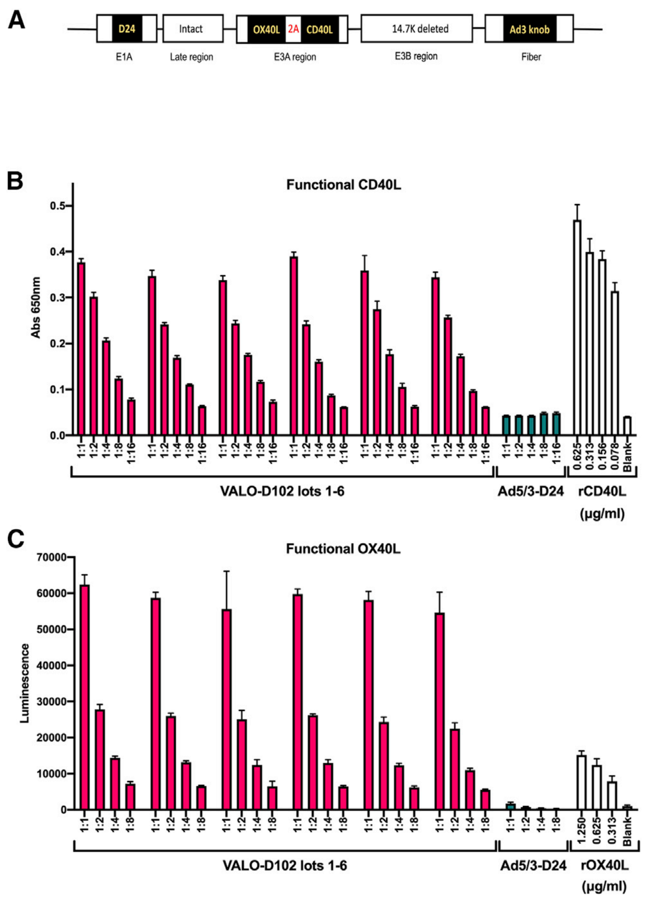

Figure 1. Novel VALO-D102 oncolytic adenovirus produces high levels of biologically active human CD40 ligand (CD40L) and OX40 ligand (OX40L)

(A) Schematic representation of genetic modifications in VALO-D102. The virus has a 24-base pair deletion in E1A; the CR1-alpha and gp19K genes in the E3A region have been replaced with human OX40L and CD40L genes; the 14.7K gene in the E3B region has been deleted; and finally, the adenovirus 5 knob domain has been replaced with the knob domain from adenovirus serotype 3. (B) A549 cells were infected with VALO-D102 at a MOI of 10. 72 h postinfection, supernatant was collected and added to a culture of Ramos-Blue reporter cells, and CD40 receptor activation by functional virus-produced CD40L was measured. (C) A549 cells were infected with VALO-D102 at a MOI of 10. 48 h postinfection, HEK293-OX40/NF-κB reporter cells were added to the infected A549 cells for 6 h, and OX40 activation by virus-expressed, functional membrane-bound OX40L was measured.

two immune-activating ligands: the ligand for cluster of differentiation 40 (CD40L) and the ligand for tumor necrosis factor receptor superfamily member 4 (OX40L). CD40L interaction with CD40 receptors on antigen-presenting cells (APCs) greatly increases their antigen presentation and costimulatory capacity and allows for efficient $CD8^+$ cytotoxic T lymphocyte (CTL) priming. OX40L interaction with OX40 receptors has an important role in the survival and homeostasis of effector and memory T cells, as well as controlling the function and differentiation of Foxp3+ regulatory T (Treg) cells.[10–12] To further enhance the anti-tumor immune responses elicited by this novel OX40L/CD40L-armed adenovirus, we used the virus with a recently developed cancer vaccine platform called PeptiCRAd.[13–15] Intratumoral administration of PeptiCRAd increased tumor-specific T cell responses, reduced tumor growth, and induced systemic anticancer immunity in mouse models of melanoma. In addition, PeptiCRAd therapy sensitized tumors to anti-PD-1 ICI therapy, increasing the number of mice responding to ICIs. The PeptiCRAd platform can induce strong tumor-specific T cell responses, directing the OV field toward personalized cancer immunotherapy. With this platform, OAds can be quickly coated with a patient's unique set of tumor-specific antigens, a prerequisite for personalized cancer immunotherapy. This novel OX40L/CD40L-armed adenovirus coated with human melanoma antigen A3 (MAGE-$A3_{(161-180)}$) and human cancer-testis antigen (NY-ESO-$1_{(91-110)}$), designated "PeptiCRAd-1," will be tested in combination with anti-PD-1 ICI in a phase I clinical trial in patients with triple-negative breast cancer (TNBC), melanoma, non-small cell lung cancer, and sarcoma.

RESULTS

Novel OAd VALO-D102 produces high levels of biologically active human CD40L and OX40L

VALO-D102 (Ad5/3-D24-OX40L-CD40L) is a serotype 5 oncolytic human adenovirus where the serotype 5 knob has been replaced by a serotype 3 knob for enhanced gene delivery into tumor tissue.[16] A 24-base pair deletion in the E1A gene in the virus genome prevents the virus from replicating in nonmalignant cells.[17,18] VALO-D102 codes for two human immunostimulatory proteins: OX40L and CD40L. A schematic representation of the VALO-D102 viral genome is shown in Figure 1A. First, we assessed the expression levels of biologically active CD40L using a human B cell-derived cell line (Ramos-Blue) stably expressing a nuclear factor κB (NF-κB)/AP-1-inducible secreted embryonic alkaline phosphatase (SEAP). The binding of CD40L to CD40 on the surface of Ramos-Blue cells triggers an intracellular signaling pathway that, via NF-κB activation, leads to the secretion of SEAP. A549 cells were infected with VALO-D102 or with the unarmed parental virus Ad5/3-D24 at a multiplicity of infection (MOI) of 10, and 72 h postinfection, the supernatants were collected, filtered, and added on Ramos-Blue cells. After 22 h incubation, CD40L-induced NF-κB activation was measured. In contrast to the unarmed Ad5/3-D24, VALO-D102 secreted large quantities of functional CD40L (approximately 150 ng/mL of the soluble form of CD40L) into the growth medium of infected A549 cells (Figure 1B) with minimal variation between multiple lots of large-scale virus preparations. In addition, high levels of functional CD40L activity produced by VALO-D102 suggest that the A2 self-cleaving peptide sequence between the OX40L and CD40L genes was able to efficiently uncouple the translation between the two transgenes (Data S1).

Next, we tested whether VALO-D102 was able to produce functional OX40L. As OX40L is mainly expressed on the cell surface,[19] we developed an activity assay using the OX40/NF-κB reporter-human embryonic kidney (HEK)293 cell line and allowed these cells to interact for 6 h with A549 cells infected 48 h earlier with VALO-D102 or with the unarmed Ad5/3-D24 at a MOI of 10. Binding of OX40L on the surface of the infected A549 cells to the OX40 receptor on the surface

of the reporter cells triggers an intracellular signaling pathway that via NF-κB activation, leads to the expression of the reporter gene. In contrast to the cells infected with the unarmed Ad5/3-D24, VALO-D102-infected cells were able to readily trigger NF-κB activation, leading to high expression of the reporter gene. Thus, VALO-D102 can produce large quantities of OX40L (approximately more than 2 μg/mL of membrane-bound OX40L), leading to high surface expression in infected cells with minimal variation between multiple lots of large-scale virus preparations (Figure 1C).

VALO-D102 can be stored at –20°C for an extended period of time and is genetically stable

Long-term storage stability of VALO-D102 in A195 buffer was assayed after storage in both −20°C and <−60°C for up to 9 months. All tested parameters, the analysis of viral particle (VP), and infectious unit (IU) titers, as well as nanoparticle tracking analysis (NTA), showed no signs of degradation, aggregation, or decrease in VP or IU titers, indicating that VALO-D102 in A195 can be stored at −20°C or <−60°C for an extended period of time (Figure 2A). For the assessment of genomic stability, VALO-D102 was put through 7 cycles of amplification, followed by deep sequencing of the viral genome. Sequencing confirmed the stability of the transgenes and other modifications, and no genetic rearrangements were seen (see Figure S5).

VALO-D102 as a component of the PeptiCRAd-1 cancer vaccine

We then tested VALO-D102 in our previously developed cancer vaccine platform, PeptiCRAd. PeptiCRAd is a cancer vaccine platform combining OAd with polylysine-modified tumor antigens.[13–15] PeptiCRAd-1 is a cancer vaccine that consists of VALO-D102 coated with human leukocyte antigen (HLA)-I epitopes from two known tumor antigens: the cancer-testis antigen NY-ESO-1 and human melanoma antigen MAGE-A3. The modified peptides used in PeptiCRAd-1 consist of a 6 lysine residue (6K)-NY-ESO-$1_{(91-110)}$ peptide containing amino acids 91 to 110 from NY-ESO-1 N-terminally fused to 6Ks and a 9 lysine residue (9K)-MAGE-$A3_{(161-180)}$ peptide containing amino acids 161 to 180 from MAGE-A3 N-terminally fused to 9Ks. First, we assessed whether the polylysine addition to the NY-ESO-1 and MAGE-A3 antigens had any effects on immunogenicity, such as antigen processing and presentation by APCs. To test this, we performed an interferon gamma (IFN-γ) enzyme-linked immune absorbent spot (ELISpot) using $CD8^+$ T cells isolated from melanoma patients NW-3568 and NW-1751. As expected, the addition of polylysine to the peptides did not affect antigen processing, presentation, or T cell activation, as both 6K-NY-ESO-$1_{(91-110)}$ and 9K-MAGE-$A3_{(161-180)}$ peptides activated T cells (as measured by IFN-γ secretion) with similar efficacy as peptides without the polylysine addition (Figure 2B). In order to assess if coating VALO-D102 with 6K-NY-ESO-$1_{(91-110)}$ or 9K-MAGE-$A3_{(161-180)}$ peptides had an effect on virus aggregation, we compared both mono-coated viruses and a mixture of the mono-coated viruses (PeptiCRAd-1) to naked VALO-D102 using NTA. As expected, the naked VALO-D102 showed insignificant amounts of aggregation with a sharp peak at 104 nanometers (nm). VALO-D102 mono-coated with 6K-NY-ESO-$1_{(91-110)}$ displayed an almost identical aggregation profile as the naked virus. VALO-D102 mono-coated with 9K-MAGE-$A3_{(161-180)}$ displayed minimal aggregation, presenting a small peak at 155 nm. PeptiCRAd-1, the mixture of both mono-coated viruses, displayed an aggregation profile almost identical to VALO-D102 mono-coated with 9K-MAGE-$A3_{(161-180)}$, indicating that the two mono-coated complexes could be mixed together with no additional aggregating effects (Figure 2C).

Oncolytic activity of VALO-D102 is not affected by the insertion of transgenes or coating of the virus with tumor antigens

To study the oncolytic potential of VALO-D102 and PeptiCRAd-1, a panel of cell lines consisting of lung cancer, melanoma, synovial sarcoma, and TNBC was infected with VALO-D102, Ad5/3-D24, and PeptiCRAd-1. The insertion of transgenes or coating VALO-D102 with 6K-NY-ESO-$1_{(91-110)}$ and 9K-MAGE-$A3_{(161-180)}$ did not have any adverse effects on the oncolytic potential in any of the cell lines tested, and the efficacy of VALO-D102 and PeptiCRAd-1 was found to be identical to the parental unarmed Ad5/3-D24 virus (Figures 3A−3E).

To assess the oncolytic potential of VALO-D102 and PeptiCRAd-1 in *in vivo* settings, groups of nude mice bearing established subcutaneous A549 lung cancer xenografts with initial volumes typically ranging between 30 mm3 and 60 mm3 were treated intratumorally, triweekly, starting on day 11 with 1×10^9 VP of VALO-D102, PeptiCRAd-1, Ad5/3Luc1 (a nonreplicating control adenovirus), or a corresponding volume of saline. Tumor growth was monitored for 36 days following the start of treatment. The groups of mice receiving saline or the nonreplicating Ad5/3Luc1 showed similar increases in tumor volumes, and by day 25 after the start of the treatment, the average volumes of the tumors had reached approximately 10× the initial volumes. The groups of mice receiving either VALO-D102 or PeptiCRAd-1 showed identical oncolytic potential and significantly reduced tumor growth as compared to the mock- or Ad5/3Luc1-treated group (Figures 3F and S1 for individual tumor growth curves). Based on these data, we conclude that the oncolytic potency of PeptiCRAd-1 was not reduced compared with the naked VALO-D102.

Virus-encoded OX40L and CD40L improve tumor growth control of the PeptiCRAd cancer vaccine and induce robust infiltration of tumor-specific $CD8^+$ effector T cells in a syngeneic mouse model of B16.OVA melanoma

In order to study the immunological effects of virus-encoded OX40L and CD40L in a PeptiCRAd cancer vaccine setting, we developed a murine surrogate version of VALO-D102 encoding murine OX40L and CD40L (VALO-mD901). As we have previously shown the superior anti-tumor efficacy of PeptiCRAd using an unarmed adenovirus within the platform as compared to the unarmed adenovirus alone,[13] here, we set up to test whether the combination of virus-encoded OX40L and CD40L has an effect on the efficacy of PeptiCRAd. To test this, we used a syngeneic mouse melanoma model B16 expressing chicken ovalbumin (OVA) as a model antigen.[20] The peptide used for coating the viruses was a polylysine-modified immunodominant

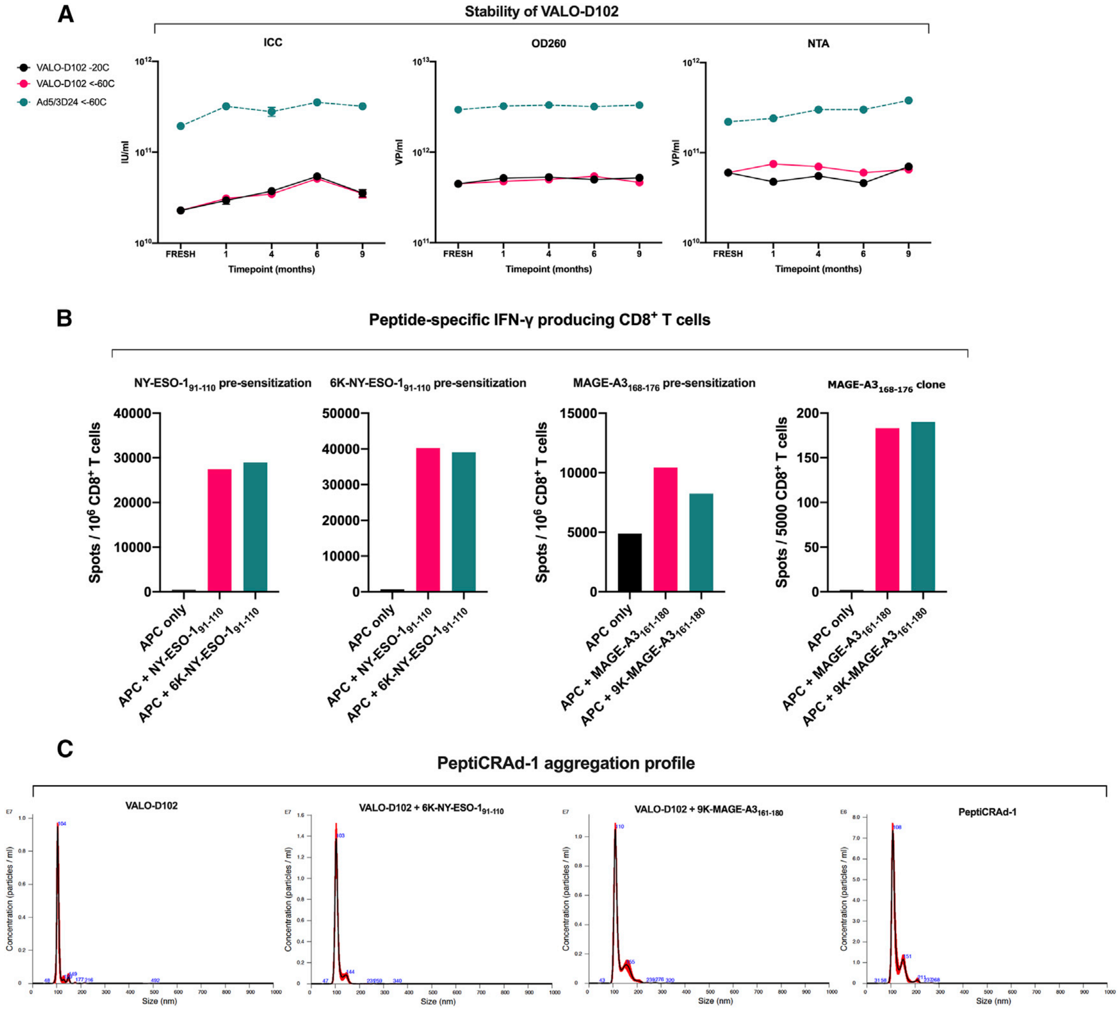

Figure 2. Characterization of PeptiCRAd-1 components

(A) VALO-D102 stability as measured by infectious titer assay (immunocytochemistry [ICC]), viral particle (VP) titer assay (OD260), and nanoparticle tracking analysis (NTA) of freshly produced virus and virus stored at −20°C and <−60°C at indicated time points. (B) Immunological characterization of the tumor antigen-containing polylysine-modified peptides used in the PeptiCRAd-1 cancer vaccine. Purified $CD8^+$ T cells from cancer patients were presensitized with peptide-pulsed (10 μg/mL), irradiated, autologous PBMCs depleted of $CD4^+$ and $CD8^+$ T cells. Presensitized $CD8^+$ T cells were tested on days 10–12 by IFN-γ ELISpot assay for recognition of peptide-pulsed (1 μg/mL), autologous antigen-presenting cells (APCs). For the NY-ESO-1 assay, $CD8^+$ T cells were presensitized with tumor antigen-containing peptides, with or without polylysine modification. APCs were pulsed with tumor antigen-containing peptides, with or without the polylysine modification. For the MAGE-A3 assay, $CD8^+$ T cells were presensitized with tumor antigen-containing peptide MAGE-A3$_{(168-176)}$. APCs were pulsed with tumor antigen-containing peptides, with or without the polylysine modification. A confirmatory ELISpot using a MAGE-A3$_{(168-176)}$-specific $CD8^+$ T cell clone combined was also performed for MAGE-A3 peptides. APCs were pulsed with tumor antigen-containing peptides, with or without the polylysine modification. Polylysine did not affect the processing or T cell recognition of the modified peptides. (C) Averaged finite track length adjustment (FTLA) concentration/size graphs from NTA of virus only (VALO-D102), VALO-D102 coated with modified NY-ESO-1 peptide, VALO-D102 coated with modified MAGE-A3 peptide, and VALO-D102 coated individually with both modified peptides followed by mixing the mono-coated complexes together (PeptiCRAd-1).

epitope (SIINFEKL) derived from the OVA antigen. Animals were treated 6, 8, and 20 days post-tumor implantation with PeptiCRAd Ad5/3-D24-OVA, PeptiCRAd VALO-mD901-OVA, or PBS as a mock-treated group. Compared to mock-treated animals, we observed clear tumor growth control in both PeptiCRAd Ad5/3-D24-OVA- and PeptiCRAd VALO-mD901-OVA-treated animals. However, PeptiCRAd VALO-mD901-OVA-treated animals showed the most efficient tumor growth control with markedly reduced tumor volumes (Figures 4A and S2 for individual tumor growth curves). Next, we assessed whether there were any differences in the infiltration of OVA-specific $CD8^+$ T cells into tumors among the treatment groups. We saw significantly higher numbers of tumor-infiltrating lymphocytes in VALO-mD901-OVA-treated tumors compared to other treatment groups. Also, the number of tumor-infiltrating

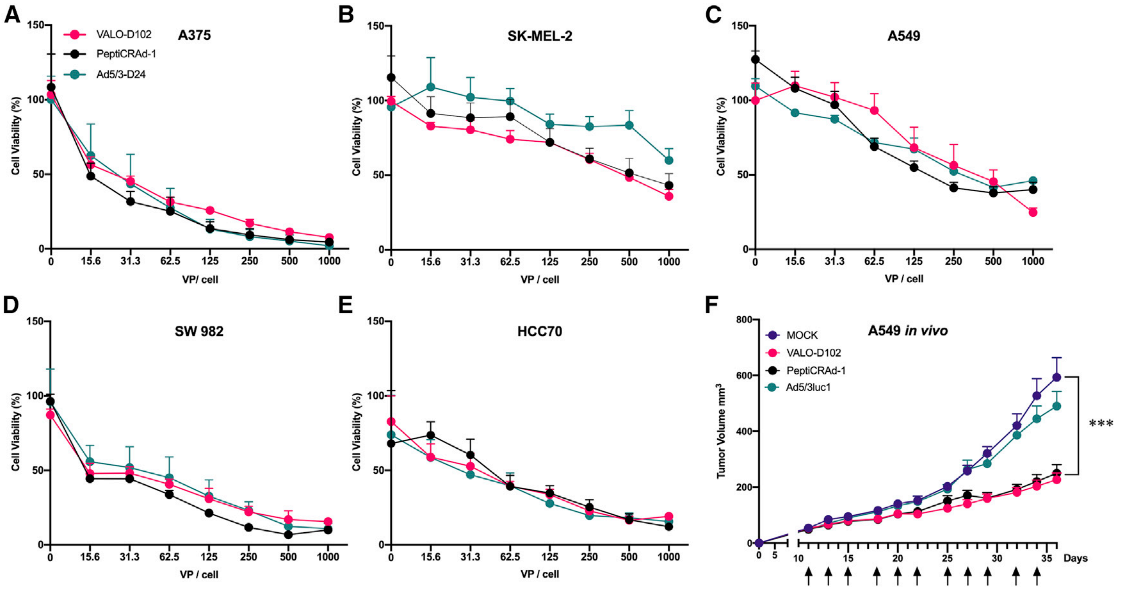

Figure 3. Oncolytic potency of VALO-D102 is identical to the parental strain and is not affected by coating the virus with tumor antigens

(A–E) Oncolytic potency of VALO-D102 and PeptiCRAd-1, a cancer vaccine platform consisting of VALO-D102 coated with two modified tumor antigens, was compared to the Ad5/3-D24 parental strain in a (A) human A375 melanoma cell line, (B) human SK-MEL-2 melanoma cell line, (C) human A549 lung carcinoma cell line, (D) human SW982 synovial sarcoma cell line, and (E) human HCC70 triple-negative breast cancer cell line. (F) Oncolytic potency of VALO-D102 and PeptiCRAd-1 was assessed *in vivo* in A549 tumor-bearing, immunodeficient female athymic nude-Foxn1-nu mice. 1×10^9 VP of each virus was given intratumorally triweekly starting at day 11. Replication-deficient Ad5/3luc1 was used as a control virus. The numbers of mice in each group were 11–12. Statistical analysis was performed with two-way ANOVA. ***$p < 0.001$.

cytotoxic $CD8^+$ T cells was significantly higher in VALO-mD901-OVA-treated tumors compared to mock- or PeptiCRAd Ad5/3-D24-OVA-treated tumors. Most importantly, we saw significantly higher numbers of tumor-infiltrating, OVA-specific cytotoxic $CD8^+$ T cells in PeptiCRAd VALO-mD901-OVA-treated tumors compared to other treatment groups. In addition, we observed noticeably higher numbers of OVA-specific $CD8^+$ T cells in pooled tumor-draining lymph node samples from PeptiCRAd VALO-mD901-OVA-treated mice compared to mock- or PeptiCRAd Ad5/3-D24-OVA-treated mice (Figure 4B).

Intratumoral PeptiCRAd treatment improves tumor control and induces systemic tumor-specific T cell responses in a syngeneic mouse model of B16.F10.9/K1 melanoma

Next, we tested the PeptiCRAd platform with VALO-mD901 in a syngeneic mouse model of B16.F10.9/K1 melanoma using a more relevant, tumor-associated antigen from tyrosinase-related protein 2 ($Trp2_{180-188}$). B16.F10.9/K1 melanoma is a derivative of a highly metastatic, low major histocompatibility complex (MHC) class I-expressing B16.F10.9 melanoma that was transfected with MHC class I H-2Kb genes to generate H-2Kb-expressing clone K1.[21] The B16.F10.9/K1 clone is considerably more responsive to cancer immunotherapies than the highly immunosuppressive parental strain B16.F10.9. Starting at 6 days post-tumor engraftment, mice were treated intratumorally with PeptiCRAd VALO-mD901-Trp2, VALO-mD901 virus alone, or saline as a mock-treated group. Although the VALO-mD901 virus alone did not show any tumor growth control, as expected since the human adenovirus 5 cannot productively replicate in murine cells, PeptiCRAd VALO-mD901-Trp2-treated mice showed significant tumor growth control as compared to mock-treated mice (Figures 5A and S3 for individual tumor growth curves). We went on to assess whether there were any differences in the Trp2-specific T cell responses among the treatment groups. The number of tumor-infiltrating, cytotoxic Trp2-specific $CD8^+$ T cells was higher (although not significantly) in PeptiCRAd VALO-mD901-Trp2-treated tumors compared to mock- or VALO-mD901-treated tumors. In addition, we observed slightly higher numbers of Trp2-specific $CD8^+$ T cells in pooled tumor-draining lymph node samples from PeptiCRAd VALO-mD901-Trp2-treated mice compared to mock- or VALO-mD901-treated tumors (Figure 5B). In striking contrast to VALO-mD901- and mock-treated mice, a very robust induction of systemic Trp2-specific T cell response was seen in PeptiCRAd VALO-mD901-Trp2-treated mice (Figure 5C).

PeptiCRAd treatment increases the number of responders to anti-PD-1 therapy and improves tumor control and survival in a syngeneic mouse model of B16.F10.9/K1 melanoma

Finally, we tested the PeptiCRAd platform with VALO-mD901 in a syngeneic mouse model of B16.F10.9/K1 melanoma in combination with anti-PD-1 ICI therapy. Starting at 9 days post-tumor engraftment, mice were treated intratumorally with PeptiCRAd VALO-

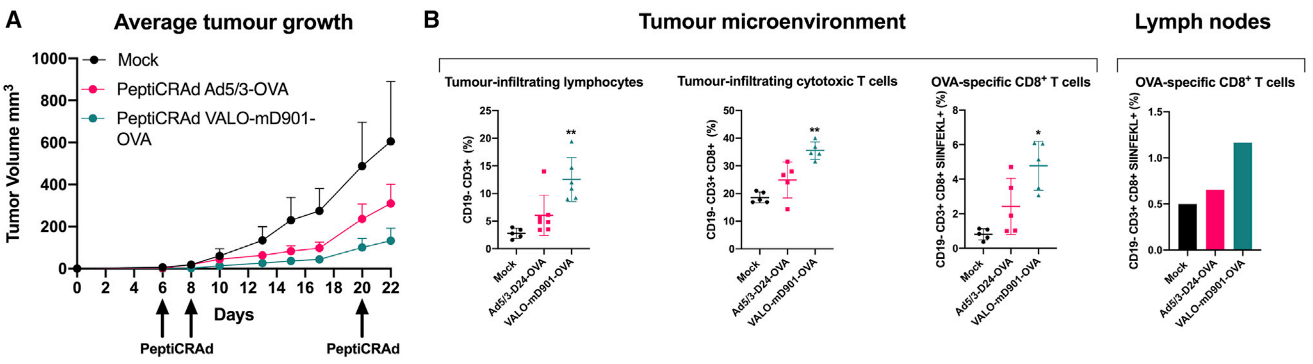

Figure 4. Virus-encoded OX40L and CD40L improve anti-tumor efficacy and induce robust infiltration of tumor-specific CD8$^+$ T cells into the tumor in a syngeneic mouse model of B16.OVA melanoma

(A) 1×10^9 VP of PeptiCRAd Ad5/3-D24-OVA or PeptiCRAd VALO-mD901-OVA was given intratumorally 6, 8, and 20 days post-tumor implantation. Average tumor growth curves for all treatment groups are shown. (B) Immunological analysis of tumors and tumor-draining lymph nodes of treated mice. Lymph nodes from all mice from each treatment group were pooled in order to get enough cells for the flow cytometric analysis. The number of mice in the mock group was 7 and in both PeptiCRAd groups, was 10. Statistical analysis was performed with one-way ANOVA. *$p < 0.05$, **$p < 0.01$.

mD901-Trp2, anti-PD-1 alone, PeptiCRAd VALO-mD901-Trp2 in combination with anti-PD-1, or saline as a mock-treated group. In contrast to mock-treated animals, PeptiCRAd VALO-mD901-Trp2, anti-PD-1 alone, and PeptiCRAd VALO-mD901-Trp2 in combination with anti-PD-1 ICI-treated groups showed significant tumor growth control. PeptiCRAd VALO-mD901-Trp2 and anti-PD-1 ICI-treated groups showed similar efficacy in controlling the tumor growth, whereas the combination therapy group showed the highest tumor control (Figures 6A and S4 for individual tumor growth curves), exhibiting the largest number of complete responders, as well as the highest survival benefit (Figure 6B).

To further evaluate the mechanism of the tumor growth control, complete responders from each group were rechallenged with 2× the original dose of the same tumor cells into the contralateral flank. All naive mice, used as engraftment control, were sacrificed by day 29 due to large tumor masses, whereas all rechallenged mice from each group showed no signs of tumors at day 29 post rechallenge, indicating an induction of systemic anti-cancer immunity by the different treatments (Figure 6C).

DISCUSSION

In this study, we have developed a double-transgene-armed adenovirus that is expressing biologically active OX40L and CD40L with levels comparable to other transgene-armed viruses reported earlier.[22–24] The oncolytic potency of VALO-D102 was shown to be identical to the parental strain Ad5/3-D24. In addition, coating VALO-D102 with modified peptide antigens from NY-ESO-1 and MAGE-A3, to obtain the clinical candidate PeptiCRAd-1, did not decrease the oncolytic potency of the vaccine, as assessed *in vitro* and *in vivo*. These results are in line with our previous studies reporting similar oncolytic potency of PeptiCRAd Ad5-D24-OVA as compared to uncoated Ad5-D24 in a human colorectal adenocarcinoma cell line and in two melanoma cell lines.[13] The genomic stability of VALO-D102 was assessed after several passages in great detail by deep sequencing of the viral genome, and no rearrangements were seen. This was expected, since the replacement of CR1-alpha and gp19K genes with the two transgenes and concomitant deletion of the 14.7K gene ensured that the net genome size of VALO-D102 was only 1.08% larger than the wild-type (WT) adenovirus 5 genome. This is well below the maximum net genome size (3%–5% larger than WT adenovirus 5 genome) in which the Ad5 virion has the ability to package efficiently.[25]

To assess the immunological effects of virally encoded OX40L and CD40L, we developed a mouse surrogate virus for VALO-D102 expressing murine OX40L and CD40L under the control of the cytomegalovirus (CMV) promoter. PeptiCRAd VALO-mD901-OVA was shown to induce superior tumor growth control compared to PeptiCRAd-OVA with the parental unarmed adenovirus (PeptiCRAd Ad5/3-D24-OVA). In addition, immunological characterization of PeptiCRAd-treated tumors revealed significant differences in all key parameters analyzed; PeptiCRAd VALO-mD901-OVA elicited a general increase in tumor infiltration by lymphocytes, as well as increased the ratio of tumor-infiltrating cytotoxic T cells compared to PeptiCRAd Ad5/3-D24-OVA. Furthermore, the expression of murine OX40L and CD40L from VALO-mD901 significantly increased the number of OVA antigen-specific T cells in tumors, as well as increased the number of OVA antigen-specific T cells in tumor-draining lymph nodes.

After establishing the enhanced immune-activating properties of virally encoded OX40L and CD40L, we moved on to test the effects of the PeptiCRAd VALO-mD901 platform on tumor growth and tumor-specific T cell induction in a syngeneic mouse model of B16.F10.9/K1 melanoma, using a more relevant tumor epitope from endogenous tumor-associated antigen $Trp2_{(180–188)}$. PeptiCRAd VALO-mD901-Trp2 was superior to the uncoated virus in

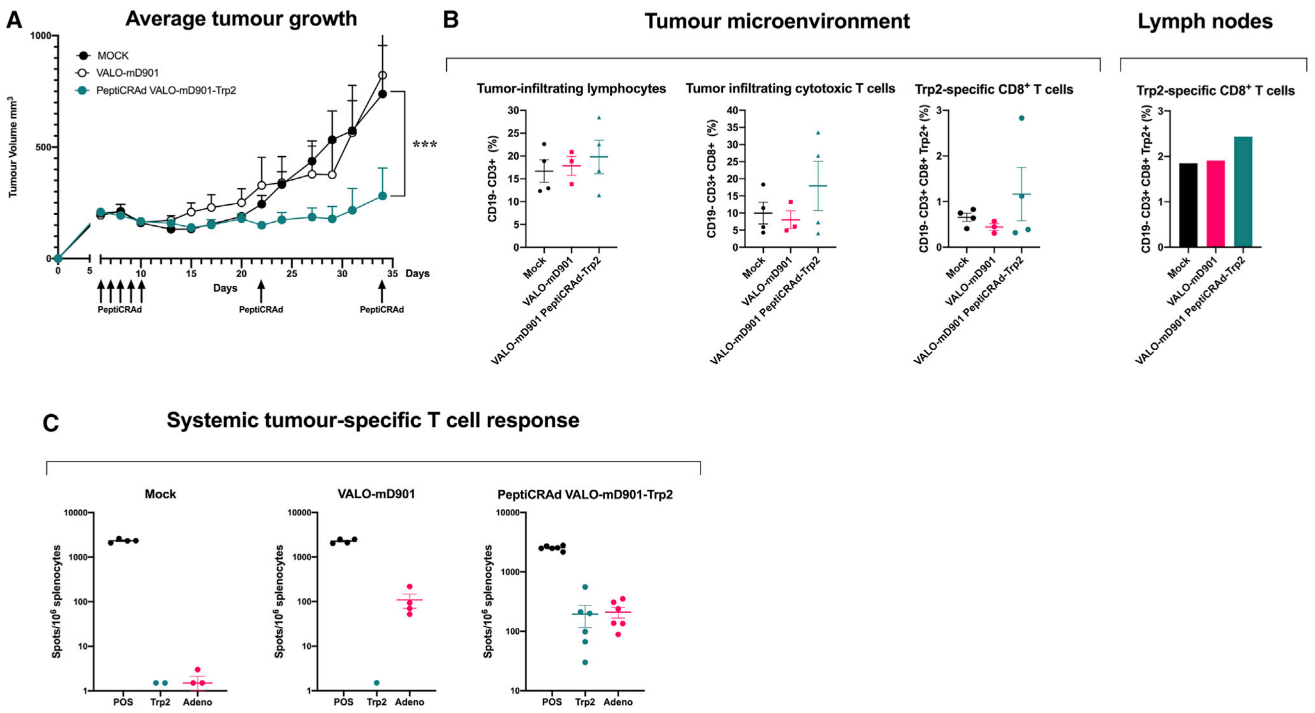

Figure 5. PeptiCRAd improves tumor growth control and induces systemic tumor-specific T cell responses in a syngeneic mouse model of B16.F10.9/K1
(A) 1×10^9 VP of VALO-mD901 or PeptiCRAd VALO-mD901-Trp2 was given intratumorally 6, 7, 8, 9, 10, 22, and 34 days post-tumor implantation. Average tumor growth curves for all treatment groups are shown. (B) Immunological analysis of tumors and tumor-draining lymph nodes of treated mice. Lymph nodes from all mice from each treatment group were pooled in order to get enough cells for the flow cytometric analysis. (C) Systemic tumor-specific T cell responses were analyzed with the ELISpot assay from the spleens of treated mice. The numbers of mice in each group were 8–10. Statistical analysis was performed with two-way ANOVA. ***$p < 0.001$.

controlling tumor growth, as well as in inducing systemic tumor-specific T cell responses. Remarkably, the intratumoral administration of PeptiCRAd VALO-mD901-Trp2 induced systemic, Trp2-specific T cell responses of similar magnitude than the T cell responses induced against the virus itself.

As we and others have recently reported enhanced effects on anti-tumor activity through the combination of OVs with ICIs[6,7,26,27], we set up to test the effect of PeptiCRAd VALO-mD901-Trp2 in combination with anti-PD-1 ICI on anti-tumor activity. Although both monotherapies were efficient at controlling tumor growth, the combination therapy of PeptiCRAd VALO-mD901-Trp2 and anti-PD-1 showed a significantly enhanced effect on tumor growth control with increased survival. By the time all mock-treated mice had died or been sacrificed due to the tumors reaching the maximum volume allowed in the PeptiCRAd VALO-mD901-Trp2-treated group and anti-PD-1-treated group, 3/9 (33.3%) and 5/8 (62.5%) mice were still alive, respectively. However, in the combination therapy group, 8/9 (88.9%) mice were still alive at that time point. Complete responders from each group were rechallenged with another injection of the same tumor cells into their contralateral flanks to assess if these mice had acquired treatment-induced systemic anti-tumor T cell immunity against the cancer cells. All naive mice presented uncontrollable tumor growth and were sacrificed at day 29 post-tumor implantation, whereas none of the complete responders from any group showed signs of tumor growth at this time point, indicating an effective systemic immunity against the injected tumor. It is important to note that human adenovirus 5 is not able to productively replicate in murine cells; thus, the effect that is seen in these murine models of melanoma is mostly due to immunological activation induced by the treatments. The effect of human adenovirus 5 replication-induced lysis of tumor cells and the release of tumor-associated antigens and neoantigens cannot be studied in these murine models. As tumor cell infection and productive replication by OVs in cancer patients lead to an inflammatory response and cytokine production, followed by infiltration of immune cells and subsequent TME repolarization toward a less immunosuppressive phenotype, these results in mice may be an underestimate of the effect of PeptiCRAd therapy in a human setting. In addition, due to the inability of human adenovirus 5 to productively replicate in murine cells and thus efficiently express viral genes or transgenes under the control of endogenous viral promoters, we placed the murine OX40L and CD40L transgenes in VALO-mD901 under the control of a CMV promoter to allow transgene expression in infected murine cells. A CMV promoter drives transgene expression in all murine cells infected, whereas VALO-D102 expresses the transgenes exclusively in cancer cells. This discrepancy in transgene expression patterns between the two viruses may induce some differences in immune activation by these viruses.

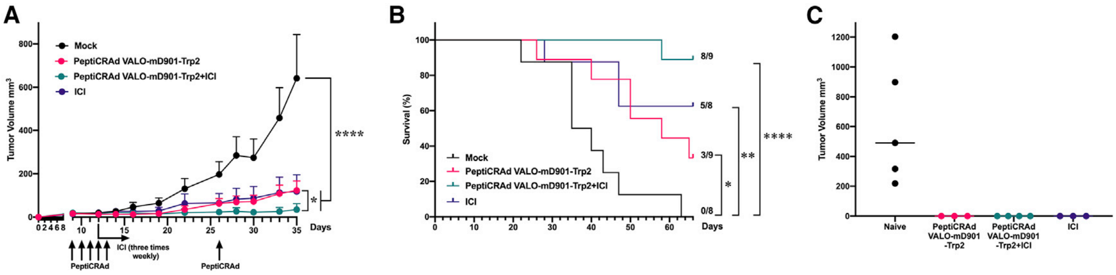

Figure 6. PeptiCRAd, in combination with anti-PD1, improves tumor growth control and survival compared to either monotherapy and triggers a systemic anti-tumor memory response in a syngeneic mouse model of B16.F10.9/K1

(A) Anti-PD-1 immune checkpoint inhibitor alone (200 μg/dose given intraperitoneally), 1×10^9 VP of PeptiCRAd VALO-mD901-Trp2 alone, or in combination with anti-PD-1 immune checkpoint inhibitor were given intratumorally 9, 10, 11, 12, 13, and 26 days post-tumor implantation. Average tumor growth curves for all treatment groups are shown. (B) Kaplan-Meier survival curve for all treatment groups. (C) Individual tumor volumes for rechallenged mice at day 29 after secondary tumor engraftment. The numbers of mice in each group were 8–9 in (A) and (B) and 3–5 in (C). Statistical analysis was performed with two-way ANOVA for (A) and with log-rank test for (B). *$p < 0.05$, **$p < 0.01$, ****$p < 0.0001$.

To summarize, the novel double-transgene-armed adenovirus VALO-D102 expresses high amounts of biologically active OX40L and CD40L, was shown to be genetically stable, and can be stored at −20°C for several months without decreases in viral VP or IU titers. PeptiCRAd-1 is a clinical oncolytic vaccine candidate comprised of VALO-D102 and two peptides containing clinically proven epitope sequences from NY-ESO-1 cancer-testis antigen[28] and MAGE-A3 melanoma-associated antigen[29] fused to polylysine linkers acting as positively charged moiety for electrostatic attachment of the peptides onto the viral capsid. PeptiCRAd-1 was shown to have similar oncolytic potency to naked VALO-D102, and a murine surrogate virus VALO-mD901 showed potent immunostimulatory properties and tumor growth inhibition in two mouse models of melanoma. In addition, enhanced anti-tumor activities were observed in animals treated with PeptiCRAd VALO-mD901-Trp2 and when combined with anti-PD-1 ICI. These findings warrant the clinical testing of PeptiCRAd-1 in combination with ICIs against the PD-1/PD-L1 pathway for the treatment of NY-ESO-1- and MAGE-A3-expressing cancers, such as TNBC, melanoma, non-small cell lung cancer, and sarcoma.

MATERIALS AND METHODS

Cell lines

Human lung carcinoma A549 cell line was purchased from NIH and was cultured in Optipro, supplemented with 10% fetal bovine serum (FBS) (Life Technologies), 1% L-glutamine, and 1% penicillin/streptomycin. Human melanoma cell line A375 was cultured in DMEM high glucose, supplemented with 10%, 1% L-glutamine, and 1% penicillin and streptomycin, and human melanoma cell line SK-MEL-2 was cultured in Eagle's minimal essential medium (EMEM), supplemented with 10% FBS, 1% L-glutamine, and 1% penicillin/streptomycin. Human TNBC HCC70 was cultured in RPMI-1640 high glucose with 10% FBS, 1% L-glutamine, and 1% penicillin/streptomycin. HEK293 cell line was cultured in MEM, supplemented with 10% FBS, 1% L-glutamine, 1% penicillin/streptomycin, 1% sodium pyruvate (Gibco), and 1% MEM nonessential amino acid (NEAA; Gibco). The human synovial sarcoma SW982 cell line was cultured in Leibowitz's L-15 media with 10% FBS, 1% L-glutamine, and 1% penicillin/streptomycin. A375, SK-MEL-2, HCC70, SW982, and HEK293 cell lines were purchased from ATCC. B16-OVA, a mouse melanoma cell line expressing chicken OVA, was kindly provided by Professor Richard Vile (Mayo Clinic, Rochester, MN, USA). B16.OVA cells were cultured in DMEM with 10% FBS, 1% L-glutamine, 1% penicillin/streptomycin, and 5 mg/mL Geneticin (Life Technologies). The B16F10.9/K1 cell line was kindly provided by Ludovic Martinet (Inserm, France) and was cultured in DMEM high glucose, supplemented with 10% FBS, 1% L-glutamine, and 1% penicillin/streptomycin. Ramos-Blue B-lymphocyte cell line (InvivoGen) was cultured in Iscove's modified Dulbecco's medium (IMDM), supplemented with 10% FBS, 1% L-glutamine, 1% penicillin/streptomycin, 100 μg/mL Normocin (InvivoGen), and 100 μg/mL Zeocin (InvivoGen) as a selective antibiotic. The reporter cell line HEK293-OX40/NF-κB (BPS Bioscience) was cultured in MEM, supplemented with 10% FBS, 1% L-glutamine, 1% penicillin/streptomycin, 400 μg/mL Geneticin, and 100 μg/mL Hygromycin B (Life Technologies). All media were purchased from Gibco, except Leibowitz's L-15, which was from ATCC. The SW982 cell line was cultivated at 37°C in 100% air. All other cell lines were cultivated at 37°C in 5% CO_2 in a humidified atmosphere. All cell lines were routinely tested for mycoplasma contamination.

Adenoviruses

The VALO-D102 virus was generated using a novel *in vitro* recombination method (submitted elsewhere). Briefly, a plasmid containing the adenovirus 5 genome with an adenovirus 3 fiber knob modification[16] and 24-base pair deletion of the gene E1A[17] (pAd5/3-D24) was used as a backbone for the replacement of the CR1-alpha and gp19K genes in the E3A region with the human OX40L gene, an A2 self-cleaving peptide sequence, and CD40L gene, as well as for the deletion of the 14.7K gene of the E3B region from the final genome. A plasmid with the modified E3 region containing the human OX40L gene, 2A self-cleaving peptide sequence, and CD40L gene, as well as the 14.7K deletion (pOX40L/CD40Ldelta14.7K), was synthesized by a

commercial vendor (Thermo Fisher Scientific). For the assembly of the full-length E3-modified genome, the OX40L, 2A sequence, CD40L, and Δ14.7K-containing linear DNA fragment from pOX40L/CD40Ldelta14.7K was obtained using a polymerase chain reaction (PCR), and the viral backbone plasmid pAd5/3-D24 was digested with SrfI and BarI restriction enzymes to release the nonmodified E3 region. For the insertion of the linear DNA fragment into the SrfI- and BarI-digested pAd5/3-D24 genome, an isothermal single-reaction method for *in vitro* homologous recombination was used for obtaining the modified circular pVALO-D102 full-length genome. All phases of the cloning were confirmed with PCR, restriction digestion analysis, and sequencing. To rescue the VALO-D102 virus, the circular plasmid pVALO-D102 was PacI digested to release the left and right inverted terminal repeats (ITRs) from the bacterial backbone, resulting in a linear viral genome. The linearized VALO-D102 viral genome was transfected into A549 cells using the Effectene transfection reagent (QIAGEN), resulting in the production of viable VPs.

The VALO-mD901 virus, a murine surrogate version of VALO-D102, was generated similarly to VALO-D102. Briefly, a part of the E3B region of pAd5/3-D24 was replaced with the human CMV promoter region, murine OX40L, 2A self-cleaving peptide sequence, murine CD40L gene, and rabbit β-globin polyadenylation signal. Ad5/3-D24 has been previously described[30]. Ad5/3luc1 is a serotype 3 receptor-targeted adenovirus containing a firefly luciferase transgene in place of the E1 region and was used as a replication-deficient control.[31] All viruses were amplified in A549 or HEK293 cells and purified on double-cesium chloride gradients and stored below −60°C in an A195 adenoviral storage buffer.[32] The VP concentration was measured at 260 nm, and IUs were determined by immunocytochemistry (ICC) by staining the hexon protein on A549 or HEK293-infected cells.

Peptides

The following peptides were used in this study: VFGIELMEVDPIGHLYIFAT (human MAGE-A3$_{(161-180)}$), KKKKKKKKK-VFGIELMEVDPIGHLYIFAT (9K-MAGE-A3$_{(161-180)}$), YLAMPFATPMEAELARRSLA (human cancer-testis antigen, NY-ESO-1$_{(91-110)}$), KKKKKK-YLAMPFATPMEAELARRSLA (6K-NY-ESO-1$_{(91-110)}$), KKKKKK-SVYDFFVWL and SVYDFFVWL (an MHC class I-restricted epitope from 6K-Trp2$_{(180-188)}$), and KKKKKK-SIINFEKL (an MHC class I-restricted epitope from chicken OVA, 6K-OVA$_{(257-264)}$). All peptides were purchased from Pepscan (Lelystad, the Netherlands).

IFN-γ ELISpot with patient-derived CD8$^+$ T cells

CD8$^+$ T cells derived from cancer patients with known NY-ESO-1/MAGE-A3 tumor antigen expression were purified with a MACS cell separation column (Miltenyi Biotec, Lund, Sweden) and presensitized with peptide-pulsed (10 μg/mL), irradiated, autologous peripheral blood mononuclear cells (PBMCs) depleted of CD4$^+$ and CD8$^+$ T cells. Presensitized CD8$^+$ T cells were tested on days 10–12 by the IFN-γ ELISpot assay for recognition of peptide-pulsed (1 μg/mL), autologous APC dendritic cells (DCs; or Epstein-Barr virus [EBV]-transformed B cells). The number of cytokine-producing, antigen-specific T cells was evaluated using the AID ELISpot Reader Classic ELR 07 (Autoimmun Diagnostika, Strassberg, Germany). The number of presensitized effector cells was 2.5×10^4 cells per well (0.5×10^4 for a T cell clone), and ELISpot incubation time was 16 h.

PeptiCRAd complex formation

PeptiCRAd complexes were prepared by mixing VALO-D102, VALO-mD901, or Ad5/3-D24 adenoviruses (in A195 storage buffer) and polyK-extended epitopes (in 0.9% saline) at a ratio of 1.8×10^5 peptides per one VP. The mixture was then incubated at room temperature for 15 min. For animal injections, the complexes were diluted further with 0.9% saline to administration volume.

CD40L functional assay

A549 cells were infected with the VALO-D102 virus at a MOI of 10. Growth medium was collected and filtrated through a 0.22-μm filter, 72 h postinfection. The filtrated supernatant was added to a culture of Ramos-Blue cells. The binding of virus produced CD40L to CD40, constitutively expressed by Ramos-Blue cells, and triggers an intracellular signaling pathway leading to the SEAP enzyme. The substrate in the Quanti-BLUE (InvivoGen) system turns purple/blue in the presence of SEAP. The concentration of SEAP was measured using a multi-well plate reader (Varioskan Flash; Thermo Labsystems) to determine the relative concentration of functional CD40L. Recombinant human CD40L (InvivoGen) was used as a standard.

OX40L functional assay

A549 cells were infected with the VALO-D102 virus at a MOI of 10. HEK293-OX40/NF-κB reporter cells were added to infected A549 cells, 2 days postinfection. The binding of OX40L to OX40 triggers an intracellular signaling pathway in HEK293-OX40/NF-κB cells that via NK-κB activation, leads to the expression of the firefly luciferase reporter gene. After 6 h incubation at 37°C, the cells were lysed with Glo Lysis Buffer (Promega), and Luciferase Assay Reagent (Promega) was added. The luciferase activity was determined with Varioskan Flash (Thermo Labsystems). Recombinant human OX40L (BPS Bioscience) was used as a standard.

Virus stability assays and aggregation profiling

The VP concentration and IUs were determined from a freshly made batch of VALO-D102 and after 1, 4, 6, and 9 months of storage at −20°C or <−60°C. NTA was performed using a NanoSight NS300 (Malvern) at indicated time points with samples diluted to 1/500−1/2,000 with 0.9% sodium chloride. The effect of inserted transgenes and other modifications on the genomic stability of VALO-D102 was assessed after 7 rounds of amplification by deep sequencing using the Illumina MiSeq System (Eurofins Genomics, Germany). NTA was also used to assess the aggregation of VALO-D102 and PeptiCRAd-1 particles. NTA was performed using NanoSight NS300 with samples diluted to 1/200−1/500 with 0.9% sodium chloride.

Cell viability assays

Human melanoma A375 and SK-MEL-2 cells, human lung carcinoma A549 cells, human synovial sarcoma SW982 cells, and human TNBC HCC70 cells were infected with various amounts of VALO-D102 and PeptiCRAd-1 or left uninfected. Cells were analyzed for their viability 5 days postinfection with the CellTiter 96 AQueous One Solution MTS Reagent (Promega), according to the manufacturer's instructions, and a multi-well plate reader (Varioskan Flash; Thermo Labsystems) was used to determine the absorbance of the samples.

Animal experiments

All animal experiments were reviewed and approved by the Experimental Animal Committee of the University of Helsinki and the Provincial Government of Southern Finland (license number ESAVI/6678/2019). Animals were kept in individually ventilated cages under standard conditions (12-h light:dark, temperature- and humidity-controlled conditions) and received *ad libitum* access to water and food. Animals were monitored daily for symptoms related to distress and pain, including hunched posture, overall activity/ability to move, and roughness of the hair coat. Tumor dimensions were measured by caliper (vertical and lateral dimensions) every second day, starting on the day tumors were first treated. All injections and tumor measurements were performed under isoflurane anesthesia.

For *in vivo* assessment of the oncolytic potency of VALO-D102 and PeptiCRAd-1, 6-week-old immunodeficient female athymic nude-Foxn1-nu mice were injected in the right flank with 5×10^6 A549 cells and were treated triweekly starting at day 11 post-tumor implantation with 1×10^9 VPs of VALO-D102, PeptiCRAd-1, Ad5/3luc1, or saline as a mock-treated group. For the B16-OVA melanoma experiment, 8- to 9-week-old immune-competent female C57BL/6JOlaHsd mice were injected in the right flank with 350,000 B16.OVA melanoma cells and were treated 6, 8, and 20 days post-tumor implantation with 1×10^9 VP of PeptiCRAd Ad5/3-D24-OVA, PeptiCRAd VALO-mD901-OVA, or PBS as a mock-treated group. Animals were sacrificed at day 24, and tumors and tumor-draining lymph nodes were harvested for immunological analysis. For the first B16F10.9/K1 melanoma experiment, 5- to 7-week-old immune-competent female C57BL/6JOlaHsd mice were injected in the right flank with 600,000 B16F10.9/K1 cells together with a 1:1 ratio of the Matrigel Basement Membrane Matrix High Concentration (Corning, USA) and were treated 6, 7, 8, 9, 10, 22, and 34 days post-tumor implantation with 1×10^9 VP of VALO-mD901, PeptiCRAd VALO-mD901-Trp2, or saline as a mock-treated group. Animals were sacrificed at day 41, and spleens and tumors were harvested for immunological analysis. For the B16F10.9/K1 melanoma experiment combining anti-PD-1, 5- to 7-week-old immune-competent female C57BL/6JOlaHsd mice were injected in the right flank with 300,000 B16F10.9/K1 cells and were treated 9, 10, 11, 12, 13, and 26 days post-tumor implantation with 1×10^9 VP of VALO-mD901, PeptiCRAd VALO-mD901-Trp2, or saline as a mock-treated group. Groups receiving anti-PD-1 (InVivoMab, USA; clone RMP1-14) were injected intraperitoneally triweekly with 200 μg/doses starting at day 12 until day 40 post-tumor implantation. For rechallenge, 600,000 B16F10.9/K1 cells were injected 70 days after the primary tumor engraftment in the contralateral flank. All mice strains were obtained from Envigo (Venray, the Netherlands).

ELISpot assays from murine splenocytes

The amount of SVYDFFVWL ($TRP2_{(180-188)}$) and adenovirus-specific, activated, IFN-γ-secreting T cells was measured by ELISpot assay (Cellular Technology, OH, USA), according to the manufacturer's instructions. Briefly, 2 μg of SVYDFFVWL peptide was used to stimulate the APCs (NB; this peptide contained only the MHC class I epitope in order to be able to rule out any unspecific stimulation, which could derive from the polylysine sequence used in the PeptiCRAd platform). After 3 days of stimulation, plates were stained and sent to Cellular Technology-Europe for counting of the spots.

Flow cytometry

The following antibodies were used in the experiments: TruStain Fc block anti-mouse and anti-human CD16/32 (101320; BioLegend), fluorescein isothiocyanate (FITC) anti-mouse CD8 (A502-3B-E; ProImmune), phycoerythrin (PE) anti-mouse CD3e (550353; BD Pharmingen), and peridinin-chlorophyll protein (PerCP) anti-mouse CD19 (115531; BioLegend). SIINFEKL epitope-specific T cells were studied using an APC-labeled H-2Kb/SIINFEKL pentamer (F093-84B-E; ProImmune). Flow cytometric analyses were performed using a BD Accuri 6C Plus Flow Cytometer (BD Biosciences). FlowJo software v.10 (FlowJo) was used for the data analysis.

Statistical analysis

Statistical analysis was performed using GraphPad Prism 8.0 software (GraphPad Software, USA). For data analysis, one-way ANOVA was used. All results are expressed as the mean ± SEM.

SUPPLEMENTAL INFORMATION

Supplemental Information can be found online at https://doi.org/10.1016/j.omto.2021.02.006.

ACKNOWLEDGMENTS

The authors thank Dr. Matthew Vaughan for his excellent editorial assistance. E.Y. acknowledges the Academy of Finland (project no. 1317206). V.C. acknowledges the European Research Council under the Horizon 2020 framework (https://erc.europa.eu); ERC-Consolidator Grant (agreement no. 681219); Jane and Aatos Erkko Foundation (project no. 4705796); HiLIFE Fellow (project no. 797011004); Cancer Finnish Foundation (project no. 4706116); Magnus Ehrnrooth Foundation (project no. 4706235); Academy of Finland; and Digital Precision Cancer Medicine Flagship iCAN.

AUTHOR CONTRIBUTIONS

E.Y., L.Y., T.R., J.K., P.P., E.J., S.P., and V.C. conceived and planned the experiments. E.Y., L.Y., M.F., B.M., P.A., H.C., A.U., S.F., A.K., J.K., and T.V. carried out the experiments. E.Y., L.Y., M.F., B.M., S.F., A.K., J.K., P.P., E.J., S.P., and V.C. contributed to the interpretation of the results. E.Y. took the lead in writing the manuscript. All

authors provided critical feedback and helped shape the research, analysis, and manuscript.

DECLARATION OF INTERESTS

V.C. is a cofounder and shareholder at Valo Therapeutics Oy. The other authors declare no competing interests.

REFERENCES

1. Schoenfeld, A.J., and Hellmann, M.D. (2020). Acquired Resistance to Immune Checkpoint Inhibitors. Cancer Cell *37*, 443–455.
2. Ku, G.Y., Yuan, J., Page, D.B., Schroeder, S.E., Panageas, K.S., Carvajal, R.D., Chapman, P.B., Schwartz, G.K., Allison, J.P., and Wolchok, J.D. (2010). Single-institution experience with ipilimumab in advanced melanoma patients in the compassionate use setting: lymphocyte count after 2 doses correlates with survival. Cancer *116*, 1767–1775.
3. Yuan, J., Adamow, M., Ginsberg, B.A., Rasalan, T.S., Ritter, E., Gallardo, H.F., Xu, Y., Pogoriler, E., Terzulli, S.L., Kuk, D., et al. (2011). Integrated NY-ESO-1 antibody and CD8+ T-cell responses correlate with clinical benefit in advanced melanoma patients treated with ipilimumab. Proc. Natl. Acad. Sci. USA *108*, 16723–16728.
4. Kaufman, H.L., Kohlhapp, F.J., and Zloza, A. (2015). Oncolytic viruses: a new class of immunotherapy drugs. Nat. Rev. Drug Discov. *14*, 642–662.
5. Breitbach, C.J., Lichty, B.D., and Bell, J.C. (2016). Oncolytic Viruses: Therapeutics With an Identity Crisis. EBioMedicine *9*, 31–36.
6. Puzanov, I., Milhem, M.M., Minor, D., Hamid, O., Li, A., Chen, L., Chastain, M., Gorski, K.S., Anderson, A., Chou, J., et al. (2016). Talimogene Laherparepvec in Combination With Ipilimumab in Previously Untreated, Unresectable Stage IIIB-IV Melanoma. J. Clin. Oncol. *34*, 2619–2626.
7. Ribas, A., Dummer, R., Puzanov, I., VanderWalde, A., Andtbacka, R.H.I., Michielin, O., Olszanski, A.J., Malvehy, J., Cebon, J., Fernandez, E., et al. (2017). Oncolytic Virotherapy Promotes Intratumoral T Cell Infiltration and Improves Anti-PD-1 Immunotherapy. Cell *170*, 1109–1119.e10.
8. Ranki, T., Pesonen, S., Hemminki, A., Partanen, K., Kairemo, K., Alanko, T., Lundin, J., Linder, N., Turkki, R., Ristimäki, A., et al. (2016). Phase I study with ONCOS-102 for the treatment of solid tumors - an evaluation of clinical response and exploratory analyses of immune markers. J. Immunother. Cancer *4*, 17.
9. Zamarin, D., and Pesonen, S. (2015). Replication-Competent Viruses as Cancer Immunotherapeutics: Emerging Clinical Data. Hum. Gene Ther. *26*, 538–549.
10. Bennett, S.R., Carbone, F.R., Karamalis, F., Flavell, R.A., Miller, J.F., and Heath, W.R. (1998). Help for cytotoxic-T-cell responses is mediated by CD40 signalling. Nature *393*, 478–480.
11. Ishii, N., Takahashi, T., Soroosh, P., and Sugamura, K. (2010). OX40-OX40 ligand interaction in T-cell-mediated immunity and immunopathology. Adv. Immunol. *105*, 63–98.
12. Schoenberger, S.P., Toes, R.E., van der Voort, E.I., Offringa, R., and Melief, C.J. (1998). T-cell help for cytotoxic T lymphocytes is mediated by CD40-CD40L interactions. Nature *393*, 480–483.
13. Capasso, C., Hirvinen, M., Garofalo, M., Romaniuk, D., Kuryk, L., Sarvela, T., Vitale, A., Antopolsky, M., Magarkar, A., Viitala, T., et al. (2015). Oncolytic adenoviruses coated with MHC-I tumor epitopes increase the antitumor immunity and efficacy against melanoma. OncoImmunology *5*, e1105429.
14. Feola, S., Capasso, C., Fusciello, M., Martins, B., Tähtinen, S., Medeot, M., Carpi, S., Frascaro, F., Ylosmäki, E., Peltonen, K., et al. (2018). Oncolytic vaccines increase the response to PD-L1 blockade in immunogenic and poorly immunogenic tumors. Oncoimmunology *7*, e1457596.
15. Tähtinen, S., Feola, S., Capasso, C., Laustio, N., Groeneveldt, C., Ylösmäki, E.O., Ylösmäki, L., Martins, B., Fusciello, M., Medeot, M., et al. (2020). Exploiting Preexisting Immunity to Enhance Oncolytic Cancer Immunotherapy. Cancer Res. *80*, 2575–2585.
16. Krasnykh, V.N., Mikheeva, G.V., Douglas, J.T., and Curiel, D.T. (1996). Generation of recombinant adenovirus vectors with modified fibers for altering viral tropism. J. Virol. *70*, 6839–6846.
17. Fueyo, J., Gomez-Manzano, C., Alemany, R., Lee, P.S., McDonnell, T.J., Mitlianga, P., Shi, Y.X., Levin, V.A., Yung, W.K., and Kyritsis, A.P. (2000). A mutant oncolytic adenovirus targeting the Rb pathway produces anti-glioma effect in vivo. Oncogene *19*, 2–12.
18. Heise, C., Hermiston, T., Johnson, L., Brooks, G., Sampson-Johannes, A., Williams, A., Hawkins, L., and Kirn, D. (2000). An adenovirus E1A mutant that demonstrates potent and selective systemic anti-tumoral efficacy. Nat. Med. *6*, 1134–1139.
19. Miura, S., Ohtani, K., Numata, N., Niki, M., Ohbo, K., Ina, Y., Gojobori, T., Tanaka, Y., Tozawa, H., Nakamura, M., et al. (1991). Molecular cloning and characterization of a novel glycoprotein, gp34, that is specifically induced by the human T-cell leukemia virus type I transactivator p40tax. Mol. Cell. Biol. *11*, 1313–1325.
20. Moore, M.W., Carbone, F.R., and Bevan, M.J. (1988). Introduction of soluble protein into the class I pathway of antigen processing and presentation. Cell *54*, 777–785.
21. Porgador, A., Feldman, M., and Eisenbach, L. (1989). H-2Kb transfection of B16 melanoma cells results in reduced tumourigenicity and metastatic competence. J. Immunogenet. *16*, 291–303.
22. Diaconu, I., Cerullo, V., Hirvinen, M.L., Escutenaire, S., Ugolini, M., Pesonen, S.K., Bramante, S., Parviainen, S., Kanerva, A., Loskog, A.S., et al. (2012). Immune response is an important aspect of the antitumor effect produced by a CD40L-encoding oncolytic adenovirus. Cancer Res. *72*, 2327–2338.
23. Koski, A., Kangasniemi, L., Escutenaire, S., Pesonen, S., Cerullo, V., Diaconu, I., Nokisalmi, P., Raki, M., Rajecki, M., Guse, K., et al. (2010). Treatment of cancer patients with a serotype 5/3 chimeric oncolytic adenovirus expressing GMCSF. Mol. Ther. *18*, 1874–1884.
24. Liu, B.L., Robinson, M., Han, Z.Q., Branston, R.H., English, C., Reay, P., McGrath, Y., Thomas, S.K., Thornton, M., Bullock, P., et al. (2003). ICP34.5 deleted herpes simplex virus with enhanced oncolytic, immune stimulating, and anti-tumour properties. Gene Ther. *10*, 292–303.
25. Bett, A.J., Prevec, L., and Graham, F.L. (1993). Packaging capacity and stability of human adenovirus type 5 vectors. J. Virol. *67*, 5911–5921.
26. Feola, S., Capasso, C., Fusciello, M., Martins, B., Tähtinen, S., Medeot, M., Carpi, S., Frascaro, F., Ylosmäki, E., Peltonen, K., et al. (2018). Oncolytic vaccines increase the response to PD-L1 blockade in immunogenic and poorly immunogenic tumors. OncoImmunology *7*, e1457596.
27. Kuryk, L., Møller, A.W., and Jaderberg, M. (2018). Combination of immunogenic oncolytic adenovirus ONCOS-102 with anti-PD-1 pembrolizumab exhibits synergistic antitumor effect in humanized A2058 melanoma huNOG mouse model. OncoImmunology *8*, e1532763.
28. Eikawa, S., Kakimi, K., Isobe, M., Kuzushima, K., Luescher, I., Ohue, Y., Ikeuchi, K., Uenaka, A., Nishikawa, H., Udono, H., et al. (2013). Induction of CD8 T-cell responses restricted to multiple HLA class I alleles in a cancer patient by immunization with a 20-mer NY-ESO-1f (NY-ESO-1 91-110) peptide. Int. J. Cancer *132*, 345–354.
29. Marchand, M., van Baren, N., Weynants, P., Brichard, V., Dréno, B., Tessier, M.H., Rankin, E., Parmiani, G., Arienti, F., Humblet, Y., et al. (1999). Tumor regressions observed in patients with metastatic melanoma treated with an antigenic peptide encoded by gene MAGE-3 and presented by HLA-A1. Int. J. Cancer *80*, 219–230.
30. Kanerva, A., Zinn, K.R., Chaudhuri, T.R., Lam, J.T., Suzuki, K., Uil, T.G., Hakkarainen, T., Bauerschmitz, G.J., Wang, M., Liu, B., et al. (2003). Enhanced therapeutic efficacy for ovarian cancer with a serotype 3 receptor-targeted oncolytic adenovirus. Mol. Ther. *8*, 449–458.
31. Kanerva, A., Mikheeva, G.V., Krasnykh, V., Coolidge, C.J., Lam, J.T., Mahasreshti, P.J., Barker, S.D., Straughn, M., Barnes, M.N., Alvarez, R.D., et al. (2002). Targeting adenovirus to the serotype 3 receptor increases gene transfer efficiency to ovarian cancer cells. Clin. Cancer Res. *8*, 275–280.
32. Evans, R.K., Nawrocki, D.K., Isopi, L.A., Williams, D.M., Casimiro, D.R., Chin, S., Chen, M., Zhu, D.M., Shiver, J.W., and Volkin, D.B. (2004). Development of stable liquid formulations for adenovirus-based vaccines. J. Pharm. Sci. *93*, 2458–2475.

OMTO, Volume *20*

Supplemental information

Characterization of a novel OX40 ligand and CD40 ligand-expressing oncolytic adenovirus used in the PeptiCRAd cancer vaccine platform

Erkko Ylösmäki, Leena Ylösmäki, Manlio Fusciello, Beatriz Martins, Petra Ahokas, Hanne Cojoc, Arttu Uoti, Sara Feola, Anna Kreutzman, Tuuli Ranki, Julia Karbach, Tapani Viitala, Petri Priha, Elke Jäger, Sari Pesonen, and Vincenzo Cerullo

Supplemental Information

Fig. S1. Individual tumour growth curves of oncolytic potency of VALO-D102 presented in Figure 3 panel F.

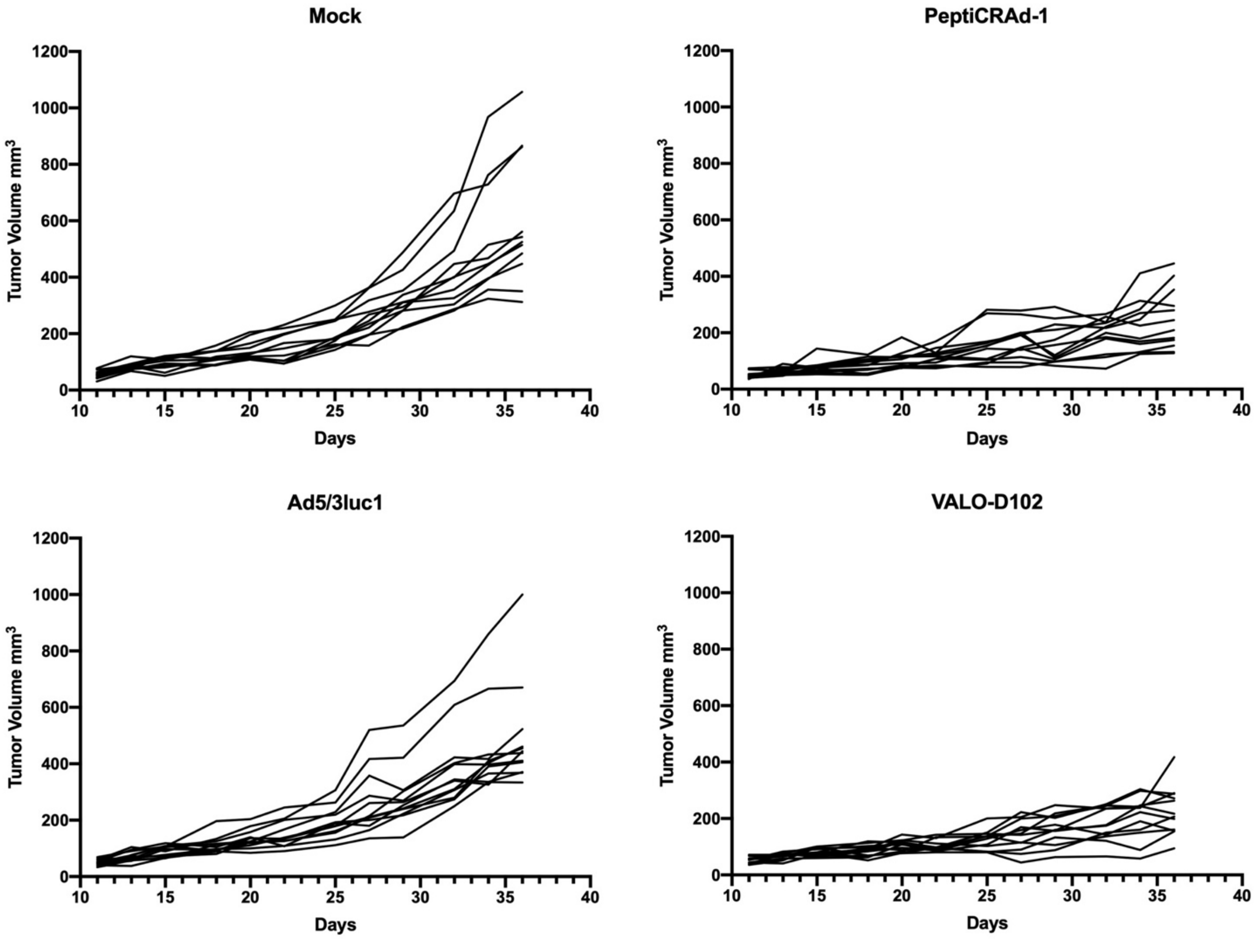

Fig. S2. Individual tumour growth curves for the experiment presented in Figure 4.

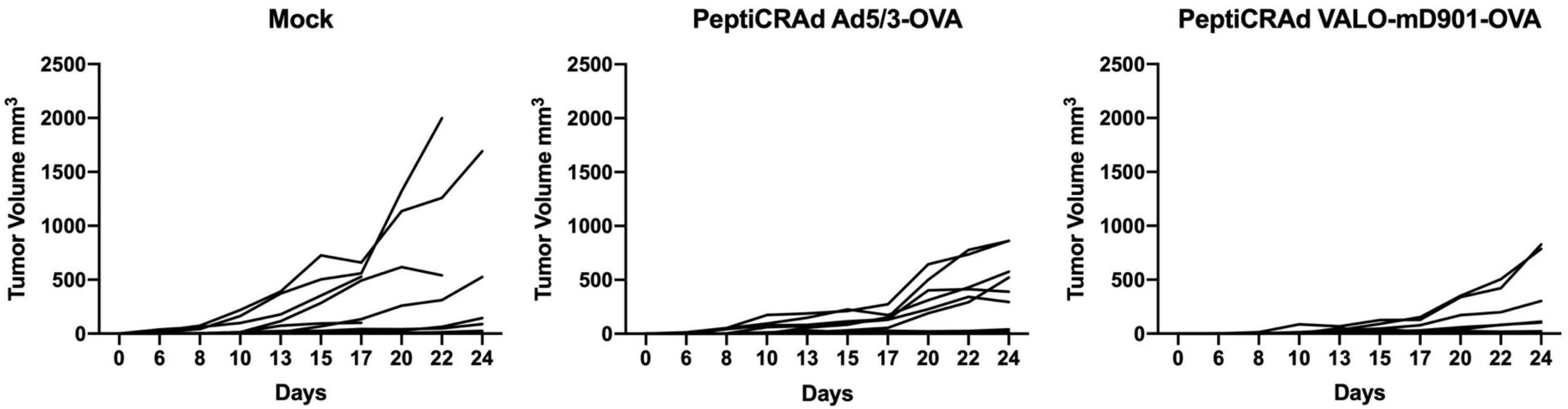

Fig. S3. Individual tumour growth curves for the experiment presented in Figure 5.

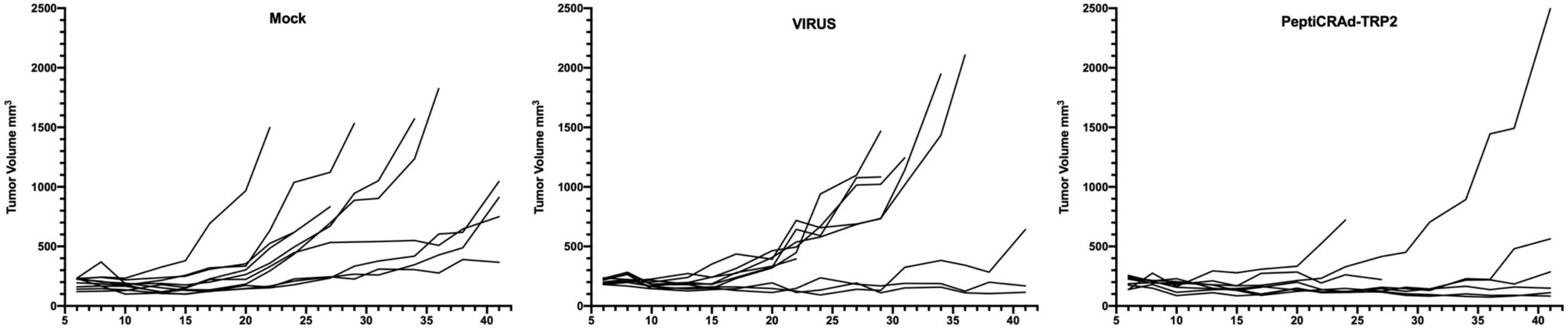

Fig. S4. Individual tumour growth curves for the experiment presented in Figure 6.

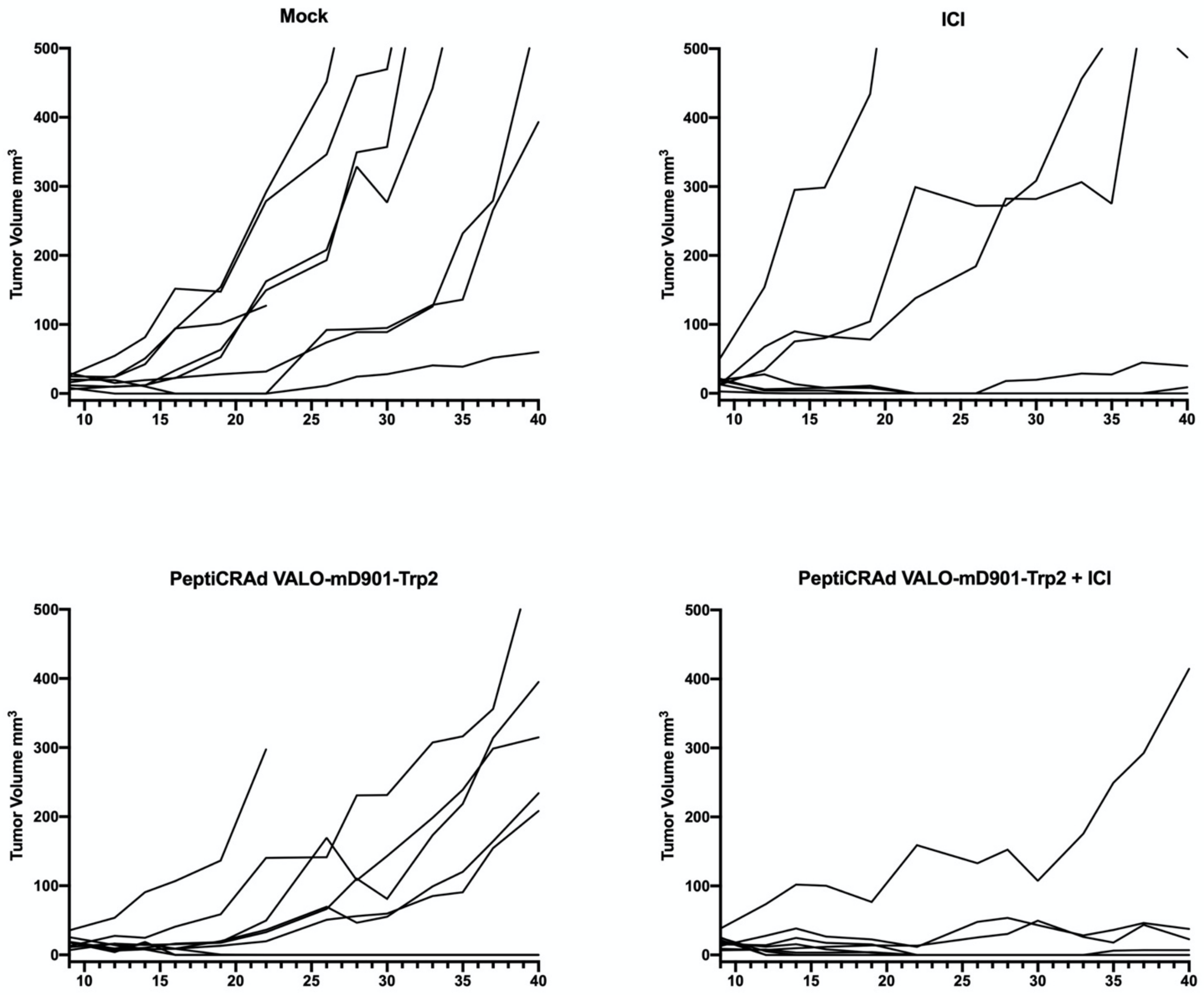

Fig. S5. Alignment of sequence analysis from directly after plaque purification and after seven rounds of amplification.
